# Oligosaccharide synthesis on soluble high-molecular weight pHEMA using a photo-cleavable linker†

**DOI:** 10.1039/c8ra08252a

**Published:** 2018-12-12

**Authors:** Abhishek Vartak, Sandeep Thanna, Kyle Meyer, Miranda Dermanelian, Steven J. Sucheck

**Affiliations:** Department of Chemistry and Biochemistry, University of Toledo 2801 West Bancroft Street Toledo Ohio 43606 USA steve.sucheck@utoledo.edu

## Abstract

Oligosaccharide synthesis on organic solvent soluble, high molecular weight poly(2-hydroxyethylmethylacrylate) (pHEMA) is described. The pHEMA-bound oligosaccharide could be recovered after each reaction in 90–95% yield using a precipitation method. The methodology was used to synthesize a model tri-galactoside in 48% overall yield and a trisaccharide from the outer core domain of *Pseudomonas aeruginosa* lipopolysacchride (LPS) in 39% yield. The use of a photo-cleavable linker is also demonstrated to produce reducing-end protected oligosaccharides.

## Abstract 2

Oligosaccharide synthesis on organic solvent soluble, high molecular weight poly(2-hydroxyethylmethylacrylate) (pHEMA) is described.

## Graphical abstract

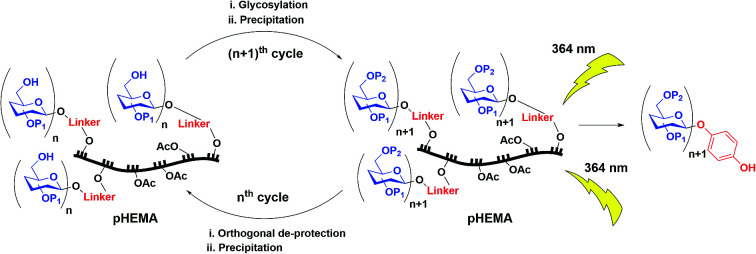

## Introduction

Oligosaccharides have been increasingly appreciated for their involvement in many biological processes; for example, cellular recognition and signalling,^1,2^ and as diagnostics for diseases such as cancer, and for their critical role as targets for immunotherapeutics.^3^ The introduction of the first polysaccharide vaccine, Pneumovax, by Merck is one such example of a successful oligosaccharide-based therapeutic. This has sparked an increased need to access oligosaccharides. However, it is a difficult task to isolate oligosaccharides from natural sources with high purity and scale. As a result, access to oligosaccharides is often achieved by synthetic methodologies. Conventional organic synthesis of oligosaccharides^4,5^ is effort intensive, in part, because of many chromatography steps. Solid-phase oligosaccharide synthesis has provided a way out of the cumbersome work-up procedures and purifications.^6–11^ However, problems such as the use of a large excess of expensive reagents and slow reaction kinetics in solid-supported systems are limitations of the methodology.

Several platforms have been developed in an attempt to accelerate oligosaccharide synthesis;^12–16^ of which, soluble polymer-bound synthesis has gained significant attention. The solubility of these polymers in organic solvents helps to reproduce polymer-free solution-based chemistry and reaction kinetics. Also, the macromolecular hydrophobic nature of soluble polymers facilitates purification using anti-solvents such as methanol or ether. Poly(ethylene glycol)methyl ether (mPEG) has been the most widely used as a soluble polymer for oligosaccharide synthesis.^17–21^ In recent years, other soluble polymers such as polystyrene,^22^ polyvinyl alcohol (PVA),^23^ and hyper-branched polyethylene glycol (PEG)^24^ have been utilized for oligosaccharide synthesis.

**Fig. 1:**
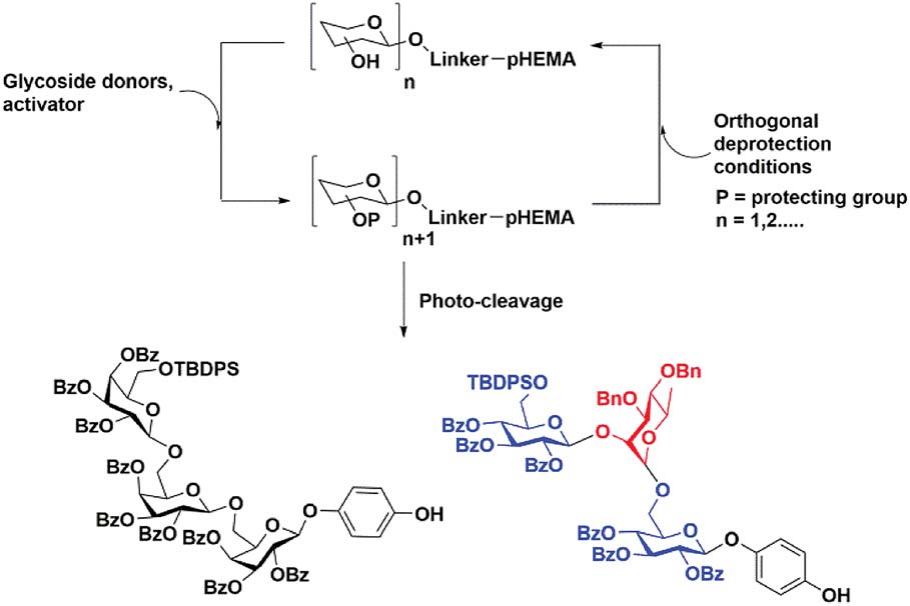
Oligosaccharide synthesis on pHEMA and photo-cleavage.

One challenge for soluble polymer platforms has been inconsistent polymer recovery using precipitation. The physicochemical properties of the selected polymer can be expected to play a crucial role in consistent precipitation recovery. In this study, we demonstrated the use of poly(2-hydroxyethylmethylacrylate) (pHEMA) of average molecular weight of 200 kDa for oligosaccharide synthesis with highly reproducible recoveries (Fig. 1).

A second challenge is the selection of a linker connecting the monosaccharide unit to polymer for oligosaccharide synthesis.^25–27^ The linker determines the scope of functional group transformations throughout the synthesis. A linker must be stable throughout multiple cycles of deprotection and glycosylation. The cleavage conditions for the linker need to be orthogonal to other saccharide protecting groups. The purification of cleaved glycan followed by global deprotection affords the desired oligosaccharide. The position of the linker on the monosaccharide unit can be on any carbon, but the most common method is the attachment at the reducing end with few exceptions.^27^ Herein, we explore our hypothesis that the *O*-nitrobenzyl motif can be selectively cleaved from pHEMA support using photo-activation^28–30^ to obtain fully protected 4-hydroxyphenyl glycosides as single anomers which can be readily purified.

An additional advantage of soluble polymer-supported syntheses is the relative ease of translation of solution phase methods to the polymer support. For example, a variety of donors such as trichloroacetimidates,^31,32^ sulfoxides,^33^ glycals^34,35^ and pentenyl glycosides^36^ have been successfully employed in soluble polymer-supported oligosaccharide syntheses. In this study, we used thioglycoside donors^24^ because of their long shelf life and ease of preparation.

For our initial studies we employed *t*-butyldiphenylsilyl (TBDPS)-protected thioglycosides. Aside from their role as protecting groups, specific protecting groups on glycosyl donors can be useful for monitoring the course of reactions. Some examples include the use of the *p*-nitrobenzoyl protecting group which can be monitored by IR spectroscopy,^37^ while 9-fluorenylmethoxycarbonyl (Fmoc) protecting group^38^ and nitrophthalimidobutyric (NPB) ester protecting group^39^ can be quantified by UV/Vis spectroscopy. Herein, we used the TBDPS group which can be monitored on pHEMA by ^1^H NMR.

## Results and discussion

**Scheme 1:**
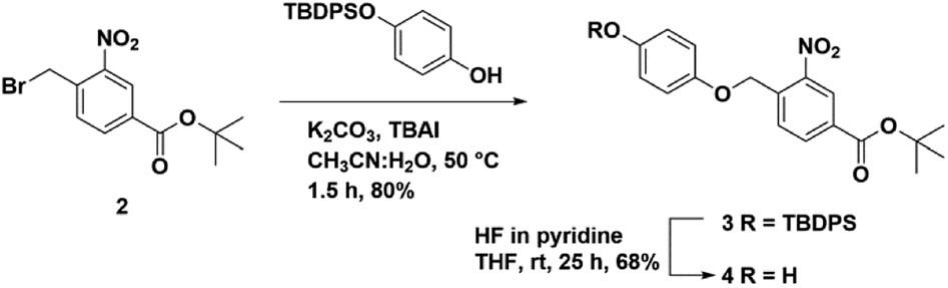
Synthesis of a hydroquinone-linked photo-cleavable linker 4.

We synthesized a novel photo-cleavable linker-bound monosaccharide building block 6 that was loaded on pHEMA using an ester linkage. The *t*-butyl 4-(bromomethyl)-3-nitrobenzoate 2 was subjected to a substitution reaction with TBDPS-protected hydroquinone (TPH)^40^ in the presence of potassium carbonate and catalytic tetrabutylammonium iodide (TBAI) in 80% yield (Scheme 1). The TBDPS group was removed using HF in pyridine to afford *t*-butyl 4-((4-hydroxyphenoxy)methyl)-3-nitrobenzoate 3 in 68% yield. We have previously shown that the TPH motif at the reducing end of the oligosaccharide is compatible with routine glycosylation condition and many functional group transformations.^40^

**Fig. 2:**
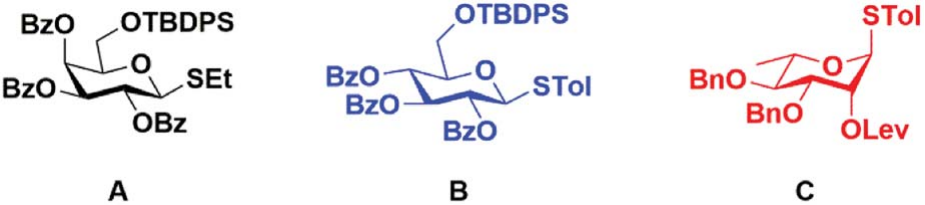
Donor building blocks A–C.

**Scheme 2:**
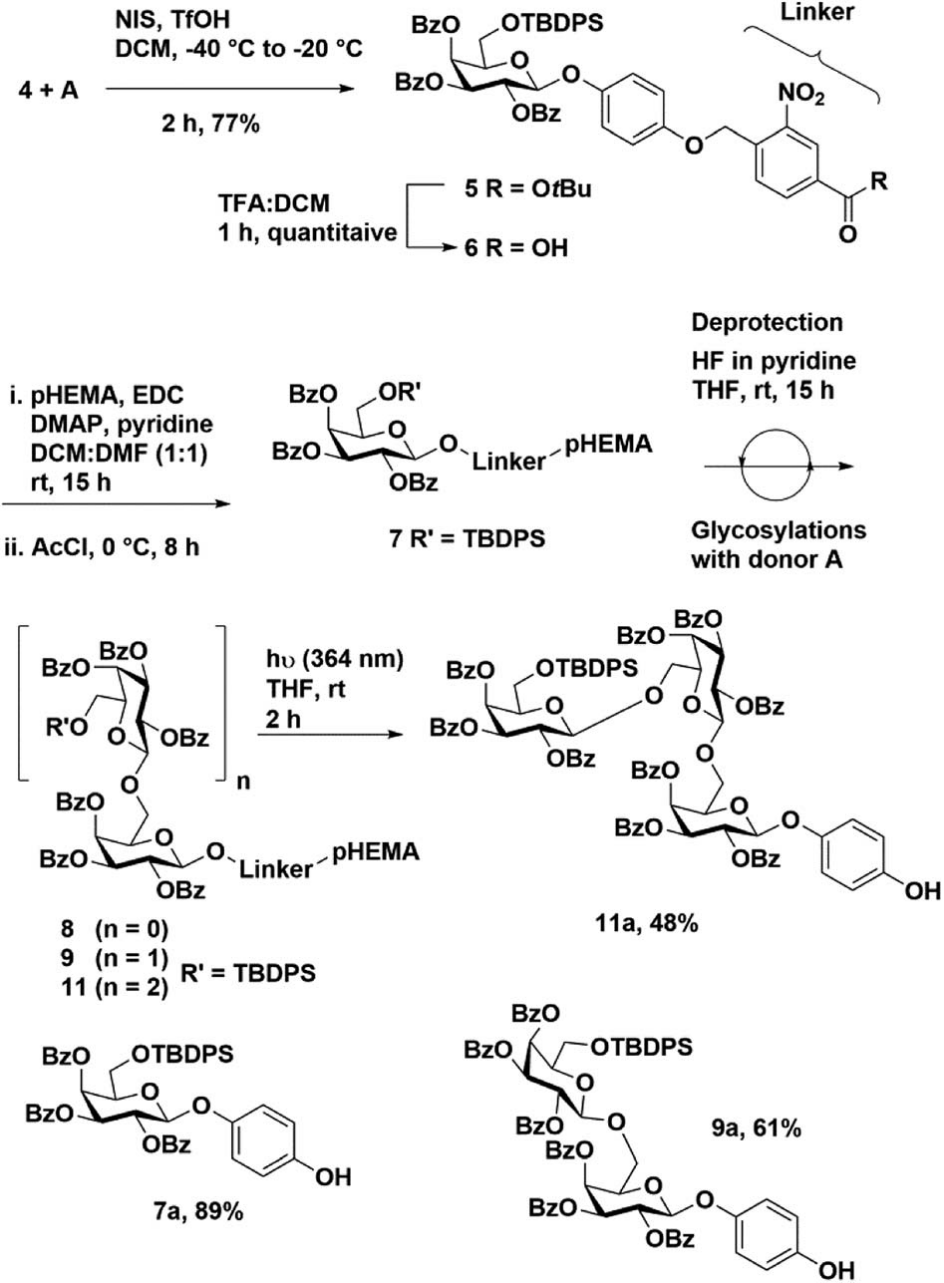
Synthesis of model mono-, di-, and tri-galactosides using a photo-cleavable linker.

To test our hypothesis, we synthesized a model trisaccharide 11a using ethyl-2,3,4-tri-*O*-benzoyl-6-*O-t*-butyldiphenylsilyl 1-thio-β-d-galactopyranoside A as a donor. The ethyl-1-thio-β-d-galactopyranoside was selectively protected using TBDPS-Cl followed by benzoylation to afford donor A in 74% yield over two steps (Fig. 2). The linker-bound monosaccharide 5 was accessed in 77% yield *via* NIS–TfOH promoted glycosylation of 4 with donor A (Scheme 2) at −40 °C to −20 °C, these conditions prevented *t*-butyl group de-protection. The *t*-butyl group on compound 5 was removed using DCM : TFA in quantitative yield to afford 4-((4-carboxylate-2-nitrobenzyl)oxy)phenyl-2,3,4-tri-*O*-benzoyl-6-*O-t*-butyldiphenylsilyl β-d-galactopyranoside 6.

Loading of carboxylate 6 on pHEMA (10 equiv.) was achieved using EDC (1.5 equiv.), DMAP (catalytic) and pyridine (25 equiv.) in a mixture of DCM : DMF at room temperature (Scheme 2). After 15 h, the temperature was lowered to 0 °C and acetyl chloride (15 equiv.) was added drop-wise. The reaction was stirred at room temperature for 8 h to cap the remaining free hydroxyl groups on the polymer. The loaded resin was extracted with ethyl acetate (EtOAc) and washed successively with 1 N HCl, aq. NaHCO_3_ and brine. After evaporating organic solvent, polymer-bound monosaccharide 7 was precipitated from EtOAc using cold methanol (20-fold dilution). The pHEMA precipitate was dried and the percentage loading was determined by ^1^H NMR to be 0.25 mmol g^−1^ using tetramethylsilane (TMS) as an internal standard.

The de-protection of the TBDPS group with a 1 M solution of TBAF in THF was unsuccessful. Therefore, excess HF in pyridine (60 equivalents) was used to obtain a monosaccharide acceptor bound to pHEMA 8. The de-protection of TBDPS was conveniently monitored on pHEMA by ^1^H NMR (Fig. S2†).

Thiogalactoside donor A and 8 were coupled using a NIS–TfOH glycosylation promoter system. Reactions were run at −20 °C for 3.5 h and only 1.5 equivalents of donor was used for each coupling. The reaction mixture was diluted with DCM and washed with aq. sodium thiosulfate, aq. sodium bicarbonate and brine. The organic layer was collected and polymer-bound disaccharide was precipitated out of the solution by diluting 20-fold with cold methanol to afford product 9 (90% polymer recovery). A second cycle of de-protection and glycosyaltion was performed to access trisaccharide bound pHEMA 10 and 11 in 92% and 89% polymer recovery, respectively (Table 1).

**Table 1:** Percentage recovery of pHEMA-bound oligosaccharide

Polymer bound product	Amount in grams		Recovery (%)
	Starting material weight (g)	Product weight (g)	
Loading and capping 7	0.66	1.08	80
1^st^ de-protection 8	0.80	0.71	94
Glycosylation 9	0.70	0.74	90
2^nd^ de-protection 10	0.54	0.46	92
Glycosylation 11	0.46	0.48	89

A high and consistent percentage recovery was observed with pHEMA compared to other polymers such as Boltron H40 which has been previously used for oligosaccharide synthesis (recovery percentage comparison is provided in Table S1†).^41^ This consistent recovery of pHEMA can be attributed to its high molecular weight and non-polar nature, once all the hydroxyl groups are capped. Isolation and recovery of oligosaccharide-bound pHEMA during synthesis using ultrafiltration with a 5 kDa molecular weight cut-off membrane was studied and found comparable to that of the precipitation.

The trisaccharide-bound pHEMA 11 was dissolved in dry THF and exposed to UV light (364 nm) for 2 h to cleave the linker and access trisaccharide 11a in 48% overall yield. The intermediate reaction steps were monitored by taking 7 and 9 (150 mg each) and subjecting the samples to photolytic cleavage. The respective 4-hydroxyphenyl glycosides 7a and 9a were obtained in 89% and 61% yield, respectively.

**Scheme 3:**
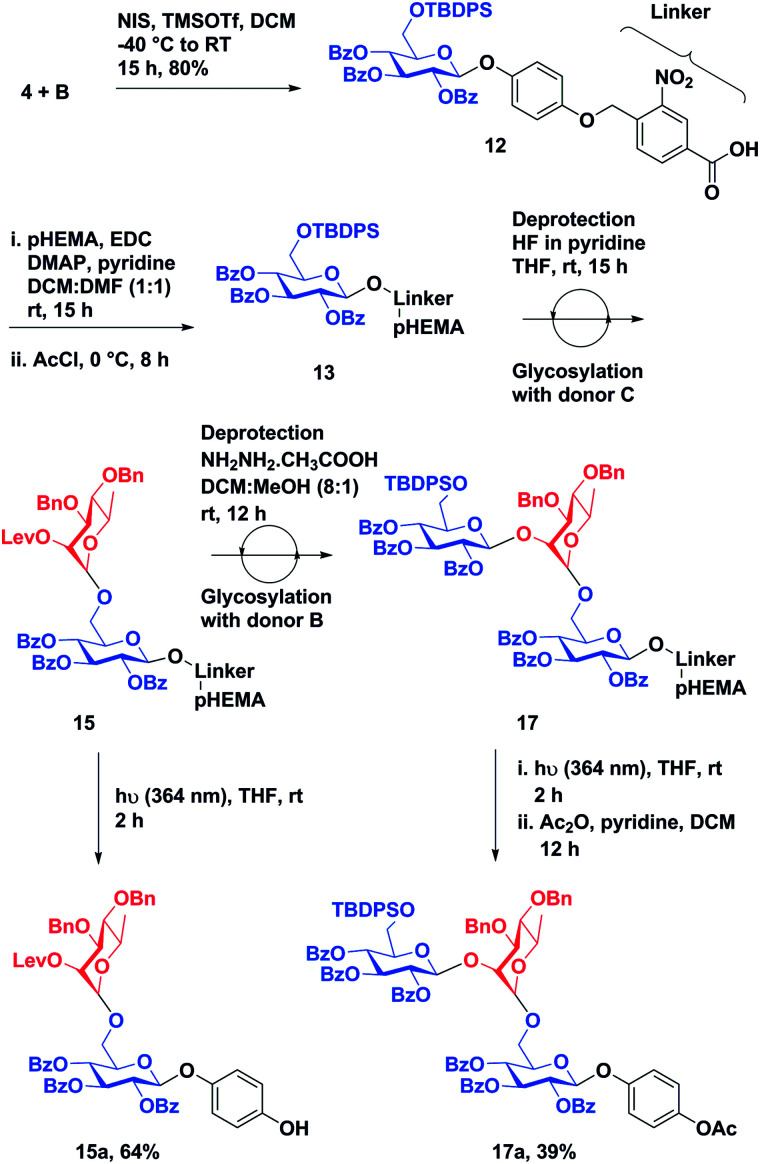
Synthesis of an outer core-trisaccharide component of *P. aeruginosa* by solution phase synthesis on pHEMA.

With a successful synthesis of a model trisaccharide 11a, a similar strategy was applied to the synthesis of a trisaccharide domain from the outer core of *P. aeruginosa* R-type lipopolysaccharide (Scheme 3). The donor building blocks B and C were synthesized according to reported literature (Fig. 2).^40^ The building block B was coupled with the photo-cleavable linker using the NIS–TMSOTf promoter system in 80% yield to access 12. The *t*-butyl group on the linker was cleaved upon warming to room temperature and standing over 12 h. The linker-bound monosaccharide 12 was loaded on pHEMA as described earlier and 0.20 mmol g^−1^ loading of monosaccharide 13 was determined by ^1^H NMR.

The TBDPS group of 13 was de-protected followed by glycosylation with donor C using the previously described protocol to obtain disaccharide-bound pHEMA 15. Disaccharide-bound pHEMA 15 (120 mg) was dissolved in dry THF and exposed to UV light (364 nm) to afford a conjugable outer core disaccharide domain of *P. aeruginosa* lipopolysaccharide 15a in 64%. The levulinate ester was deprotected using hydrazine acetate in a DCM : methanol (8 : 1) to access a pHEMA-bound disaccharide acceptor.^21^ The final glycosylation was achieved using the NIS : TfOH promoter system and donor B in 3.5 h. Trisaccharide 17a was accessed in 39% yield by subjecting compound 17 to UV (364 nm) exposure followed by acetylation. The percentage recovery of polymer-bound oligosaccharide after each reaction step is reported in Table 2.

**Table 2:** Percentage recovery of polymer bound oligosaccharide

Polymer bound product	Amount in grams		Recovery (%)
	Starting material weight (g)	Product weight (g)	
Loading and capping 13	0.16	0.30	86
TBDPS de-protection 14	0.30	0.26	92
Glycosylation 15	0.26	0.27	94
Levulinate de-protection 16	0.15	0.13	94
Glycosylation 17	0.11	0.12	90

## Conclusions

In conclusion, we have demonstrated oligosaccharide synthesis on commercially available, low cost, high molecular weight pHEMA using a very accessible photo-cleavable linker. This is a first report of pHEMA as a soluble platform for oligosaccharide synthesis. High polymer recoveries were easily achieved by precipitation of pHEMA-bound intermediates. The soluble support afforded the synthesis of two trisaccharides 11a and 17a in 48% and 39% overall yield, respectively. The polymer-bound intermediates were found to be compatible with a glycosylation and common de-protection conditions. A photo-cleavable linker was used to access 4-hydroxyphenyl glycosides which have a locked anomeric configuration to facilitate purification. The hydroquinone at the reducing end should be useful for late-stage conjugation with peptides or lipids to assemble desired glycoconjugates. Alternatively, the reducing end group can be removed by oxidative condition to produce free reducing sugars.

## Experimental

### General methods

All fine chemicals and solvents were obtained from Acros Organics, Fisher Scientific, Alfa-Aesar and Sigma-Aldrich. The solvents were purified using a PureSolv MD5 Solvent Purification System (SPS). Reactions were monitored using thin-layer chromatography (silica gel 60, f254) and spots were observed by UV light or by charring (5% H_2_SO_4_ in MeOH). Flash column chromatography was performed on silica gel (230–400 mesh) obtained from Sorbent Technologies using solvents as received. ^1^H-NMR were carried out using a Bruker Avance III 600 MHz spectrometer using residual CHCl_3_ or MeOD as internal references. ^13^C NMR were recorded at 150 MHz using residual CHCl_3_ or MeOD as internal references. High resolution mass spectroscopy (HRMS) was performed on a Micromass Q-TOF 2 instrument.

### Ethyl-2,3,4-tri-*O*-benzoyl-6-*O-t*-butyldiphenylsilyl 1-thio-β-d-galactopyranoside (A)

Imidazole (1.6 g, 23 mmol) and TBDPS-Cl (5.3 mL, 20 mmol) were added to a solution of ethyl-1-thio-β-d-galactopyranoside (3.5 g, 15 mmol) in dry DMF (10 mL) and stirred under N_2_ at room temperature (RT). The reaction was monitored by TLC and appeared complete after 2.5 h. The reaction mixture was poured over ice-cold water and extracted with EtOAc (2 × 25 mL). The organic layer was separated and dried over anhydrous Na_2_SO_4_. The solvent was evaporated and dried. The colorless oil was used in the next reaction without further purification. The colorless oil was dissolved in anhydrous pyridine (10 mL, 137 mmol) followed by addition of DMAP (0.30 g, catalytic). The reaction was cooled down to 0 °C and BzCl (10 mL, 76 mmol) was added. The reaction was stirred overnight under N_2_ at RT. The reaction mixture was diluted with DCM (200 mL), washed with 1 N HCl (3 × 100 mL) and brine (3 × 100 mL). The organic layer was collected, dried over anhydrous Na_2_SO_4_ and evaporated. The residue was subjected to flash column chromatography on silica gel (Hex : EtOAc, 8 : 2) to afford ethyl-2,3,4-tri-*O*-benzoyl-6-*O-t*-butyldiphenylsilyl 1-thio-β-d-galactopyranoside A as colorless foamy solid (8.7 g, 74% over 2 steps). *R*_f_: 0.83 (20% EtOAc/hexane); ^1^H NMR (600 MHz, CDCl_3_): *δ* 7.12–8.14 (m, 25H, Ar–H), 6.10 (dd, *J* = 3.3 Hz, 1H, H-4), 5.76 (t, *J* = 9.9 Hz, 1H, H-2), 5.66 (dd, *J* = 9.96 Hz, 1H, H-3), 4.78 (d, *J* = 9.9 Hz, 1H, H-1), 4.1 (m, 1H, H-5), 3.85 (dd, *J* = 10.2 Hz, 1H, H-6a), 3.78 (dd, *J* = 10.2 Hz, 1H, H-6b), 2.79 (q, *J* = 25 Hz, 2H, –SCH_2_), 1.29 (t, *J* = 11.7 Hz, 3H, 
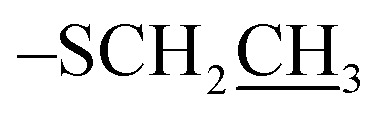
), 1.02 (s, 9H, *t*-Bu of TBDPS); ^13^C NMR (150 MHz, CDCl_3_): *δ* 165.6, 127.8–138.1 (Ar–C), 84.1, 77.8, 73.2, 68.6, 68.3, 61.5, 26.8, 24.2, 19.2, 15.1; HRMS data: [M + H] *m*/*z*: calcd for C_45_H_46_O_8_SSi, 775.2761; found, 775.2734.

### *t*-Butyl 4-((4-((*t*-butyldiphenylsilyl)oxy)phenoxy)methyl)-3-nitrobenzoate (3)

*t*-Butyl 4-(bromomethyl)-3-nitrobenzoate 2 (1.2 g, 3.8 mmol) was dissolved in a mixture of CH_3_CN : H_2_O (10 : 1, 22 mL) followed by addition of K_2_CO_3_ (1.1 g, 7.6 mmol) and tetrabutylammonium iodide (0.35 g, 0.95 mmol) (TBAI) under N_2_ at RT. The reaction mixture was stirred for 15–20 minutes followed by addition of 4-((*t*-butyldiphenylsilyl)oxy)phenol (1.5 g, 4.2 mmol). The reaction was stirred under N_2_ at 50 °C and monitored by TLC. Complete disappearance of starting material was observed after 2 h. The reaction solution was concentrated to dryness under reduced pressure and taken up in DCM (40 mL). The organic layer was then washed with 1 N HCl (2 × 100 mL) and brine. The organic layer was collected, dried over anhydrous Na_2_SO_4_, and evaporated. The residue was subjected to flash column chromatography on silica gel (Hex : EtOAc, 8 : 2) to afford compound 3 as yellow liquid (1.7 g, 80%). *R*_f_: 0.88 (20% EtOAc/hexane); ^1^H NMR (600 MHz, CDCl_3_) *δ* = 1.13 (s, 9H, *t*-butyl), 1.65 (s, 9H, *t*-butyl), 5.41 (s, 2H, –CH_2_), 6.7–8.69 (17H, aromatic); ^13^C NMR (150 MHz, CDCl_3_) *δ* = 19.6, 22.8, 26.6, 28.3, 31.7, 34.8, 67.4, 82.7, 115.7, 120.5, 125.9, 127.9, 128.7, 130.0, 132.5, 133.1, 134.4, 135.7, 138.5, 146.8, 150.4, 152.2, 163.6; HRMS data: [M + Na] *m*/*z*: calcd for C_34_H_37_NO_6_SiNa, 606.2288; found, 606.2302.

### *t*-Butyl 4-((4-hydroxyphenoxy)methyl)-3-nitrobenzoate (4)

*t*-Butyl 4-((4-((*t*-butyldiphenylsilyl)oxy)phenoxy)methyl)-3-nitrobenzoate 3 (1.7 g, 2.9 mmol) was dissolved in a Falcon tube with dry THF (20 mL) followed by addition of excess of HF in pyridine (70% HF, 2 mL, 30 equiv.). The reaction was stirred overnight and complete consumption of starting material was observed on TLC. The solvent was concentrated under reduced pressure and residue was dissolved in DCM (50 mL). The organic layer was then washed with 1 N HCl (3 × 20 mL) followed by aq. sodium bicarbonate (2 × 20 mL). The organic layer was collected and subjected to flash column chromatography on silica gel (Hex : EtOAc, 8 : 2) to afford compound 4 as pale yellow solid (0.68 g, 68%). *R*_f_: 0.35 (20% EtOAc/hexane); ^1^H NMR (600 MHz, CDCl_3_): *δ* = 1.63 (s, 9H, *t*-butyl), 5.46 (s, 2H, –CH_2_), 6.78–8.71 (7H, aromatic); ^13^C NMR (150 MHz, CDCl_3_): *δ* = 26.7, 28.4, 67.5, 82.8, 115.8, 120.6, 126.2, 127.9, 128.9, 130.1, 132.8, 133.2, 134.6, 135.8, 138.6, 146.8, 150.6, 152.4, 163.8; HRMS data: [M + H] *m*/*z*: calcd for C_18_H_20_NO_6_, 346.1291; found, 346.1302.

### 4-((4-(*t*-Butoxycarbonyl)-2-nitrobenzyl)oxy)phenyl-2,3,4-tri-*O*-benzoyl-6-*O-t*-butyldiphenylsilyl β-d-galactopyranoside (5)

Acceptor 4 (0.60 g, 1.7 mmol) and thiglycoside donor A (1.5 g, 1.9 mmol) were dissolved in dry DCM (20 mL) followed by addition of 4 Å molecular sieves and stirred for 30 minutes under N_2_. NIS (0.57 g, 2.5 mmol) and TfOH (0.20 mL, 0.34 mmol) were added to the reaction at −40 °C. The solution was then allowed to warm up to −20 °C. The reaction was observed to be complete after 2 h. The reaction was diluted with DCM (30 mL) and filtered. The filtrate was washed with aq. sodium thiosulfate (2 × 30 mL), aq. sodium bicarbonate (2 × 30 mL) and brine (2 × 30 mL). Organic layer was collected, dried over anhydrous Na_2_SO_4_ and subjected to flash column chromatography (Hex : EtOAc, 8 : 2) on silica gel to afford compound 5 as yellow foamy solid (1.4 g, 77%). *R*_f_: 0.75 (20% EtOAc/hexane); ^1^H NMR (600 MHz, CDCl_3_): *δ* 7.16–8.05 (Ar–H), 6.99 (d, *J* = 8.9 Hz, 2H, hydroquinone *para*-H), 6.81 (d, *J* = 8.9 Hz, 2H, hydroquinone *ortho*-H), *δ* 6.04 (m, 1H, H-4), 5.96 (dd, *J* = 10.4, 8.0 Hz, 1H, H-2), 5.65 (dd, *J* = 10.4, 3.4 Hz, 1H, H-3), 5.45 (s, 2H, 
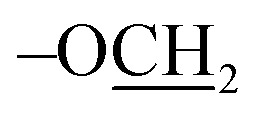
), 5.22 (d, *J* = 8.0 Hz, 1H, H-1), 4.17 (t, *J* = 6.7 Hz, 1H, H-5), 3.89 (m, 2H, H-6,6′), 1.63 (s, 9H, *t*-Bu), 1.02 (s, 9H, *t*-Bu of TBDPS); ^13^C NMR (150 MHz, CDCl_3_): *δ* = 165.81, 165.61, 165.45, 163.54, 115–155 (aromatic-C), 101.25, 82.80, 74.60, 72.08, 69.98, 68.04, 67.39, 61.90, 28.31, 26.85, 19.22; ESI-MS data: [M + Na] *m*/*z*: calcd for C_61_H_59_NO_14_SiNa, 1080.3; found, 1080.3.

### 4-((4-Carboxylate-2-nitrobenzyl)oxy)phenyl-2,3,4-tri-*O*-benzoyl-6-*O-t*-butyldiphenylsilyl β-d-galactopyranoside (6)

The compound 5 (1.2 g, 1.1 mmol) was dissolved in a mixture of DCM : TFA (1 : 1, 6 mL) and stirred at RT under N_2_. The reaction appeared complete after 1.5 h on TLC. The reaction mixture was evaporated. The residual TFA was evaporated using DCM–hexane azeotrope to afford compound 6 as colorless oil (1.1 g, quantitative) which was used for next reaction without further purification. *R*_f_: 0.15 (40% EtOAc/hexane); ^1^H NMR (600 MHz, CDCl_3_): 7.16–8.05 (Ar–H), 7.01 (d, *J* = 8.9 Hz, 2H, hydroquinone *para*-H), 6.82 (d, *J* = 8.9 Hz, 2H, hydroquinone *ortho*-H), 6.03 (d, *J* = 2.8 Hz, 1H, H-4), 5.96 (dd, *J* = 10.8, 7.6 Hz, 1H, H-2), 5.68 (m, 1H, H-3), 5.47 (s, 2H, –OCH_2_), 5.24 (d, *J* = 7.6 Hz, 1H, H-1), 4.18 (t, *J* = 6.7 Hz, 1H, H-5), 3.88 (m, 2H, H-6,6′), 1.01 (1.02 (s, 9H, *t*-Bu of TBDPS)); ^13^C NMR (150 MHz, CDCl_3_): *δ* = 168.86, 165.96, 165.75, 165.58, 115–155 (aromatic-C), 101.21, 74.60, 72.14, 70.04, 68.09, 67.40, 61.89, 26.86, 19.22; HRMS data: [M + H] *m*/*z*: calcd for C_57_H_51_NO_14_Si, 1001.3000; found, 1001.2750.

### General procedure for linker-bound monosaccharide loading on pHEMA

The linker-bound monosaccharide 6 (0.50 g, 0.50 mmol) was dissolved in a mixture of dry DCM : DMF (1 : 1, 30 mL) followed by addition of pHEMA (0.66 g, 4.9 mmol), EDC (0.15 g, 0.74 mmol), DMAP (15 mg, cat.), and pyridine (0.80 mL, 12 mmol). The reaction mixture was stirred overnight (15 h) under N_2_ at RT. The reaction was cooled to 0 °C and AcCl (0.50 mL, 7.3 mmol) was added to cap the remaining free hydroxyls on polymer. The reaction was stirred for additional 8 h at RT. The reaction mixture was evaporated under reduced pressure and then poured over ice-cold water. The aqueous phase was extracted with EtOAc (2 × 50 mL) and the organic layer washed with 1 N HCl (2 × 30 mL), aq. NaHCO_3_ (2 × 30 mL), and brine (2 × 50 mL). The organic layer was collected and dried over Na_2_SO_4_. The solvent was concentrated down to 8–10 mL and polymer was precipitated out of the solution using cold methanol (200 mL, ×20 dilution). The polymer bound monosaccharide 7 was then dried on high-vacuum (1.08 g, 80% polymer recovery). The percentage loading of monosaccharide was determined by ^1^H NMR to be 0.25 mmol g^−1^ using tetramethylsilane (TMS) as an internal standard (calculation in ESI†).

### General procedure for TBDPS deprotection on oligosaccharide bound pHEMA

pHEMA bound TBDPS protected monosaccharide 7 was dissolved in a Falcon tube with dry THF (50 mL) followed by addition of HF in pyridine (70% HF, 0.8 mL, 60 equiv.). The reaction mixture was stirred for 12 h at RT and solvent was evaporated under reduced pressure. The residue was dissolved in EtOAc and washed with 1 N HCl (3 × 30 mL), aq. NaHCO_3_ (3 × 30 mL), and brine (3 × 30 mL). The organic layer was collected, dried over Na_2_SO_4_, and concentrated to 8–10 mL of solution. The de-protected oligosaccharide-bound polymer 8 was precipitated out of the solution using ice-cold methanol (0.71 g, 94% polymer recovery). The complete de-protection was confirmed using ^1^H NMR of oligosaccharide bound pHEMA as shown in Fig. S2.†

### General procedure for NIS–TfOH glycosylation on oligosaccharide-bound pHEMA

The polymer-bound acceptor 8 (0.70 g, 0.18 mmol) and thioglycoside donor A (0.20 g, 0.26 mmol) were dissolved in dry DCM (15 mL) followed by addition of activated 4 Å molecular sieves and stirred for 30 min under N_2_ at RT. The reaction was brought to −20 °C and NIS (60 mg, 0.26 mmol)–TfOH (30 μL) was added. The reaction mixture was stirred for 3.5 h and allowed to warm up to RT. The solution was filtered to remove molecular sieves and the organic layer was washed with aq. sodium thiosulfate (3 × 30 mL), aq. sodium bicarbonate (3 × 30 mL) and brine (3 × 30 mL). The organic layer was collected, dried over anhydrous Na_2_SO_4_ and concentrated down to 8–10 mL. The disaccharide-bound polymer 9 was precipitated out of the solution using cold methanol (0.74 g, 90% polymer recovery).

A second cycle of de-protection and glycosylation was performed using the same reaction condition described earlier to afford trisaccharide-bound polymer 11 (0.48 g, 89% polymer recovery).

### General procedure for photo-activated cleavage

The polymer bound oligosaccharides (7, 9, 11 and 15) were dissolved in dry THF and purged with N_2_. The reaction mixture was exposed to a light of 364 nm wavelength and stirred at RT in a photochemical reactor for 2 h. The complete cleavage of oligosaccharide was monitored by TLC (Hex : EtOAc, 7 : 3). The reaction mixture was evaporated and dissolved in EtOAc (20 mL). The free polymer was precipitated out of the solution using cold methanol (100 mL) and filtered off. The filtrate was then subjected to flash column chromatography (Hex : EtOAc) on silica gel to afford completely protected 4-hydroxyphenyl oligosaccharide (7a, 9a, 11a and 15a).

### 4-Hydroxyphenyl-2,3,4-tri-*O*-benzoyl-6-*O-t*-butyldiphenylsilyl-β-d-galactopyranoside (7a)

Compound 7 (150 mg) was dissolved in dry THF (10 mL) and procedure mentioned above was followed. Product 7a: white solid (27 mg, 89%). *R*_f_: 0.35 (40% EtOAc/hexane) ^1^H NMR (600 MHz, CDCl_3_): *δ* 7.16–7.65 (Ar–H), 6.92 (d, *J* = 8.9 Hz, 2H, hydroquinone *para*-H), 6.67 (d, *J* = 8.9 Hz, 2H, hydroquinone *ortho*-H), 6.02 (dd, *J* = 2.82, 3.42 Hz, 1H, H-4), 5.95 (dd, *J* = 7.98, 7.27 Hz, 1H, H-2), 5.64 (dd, *J* = 7.27, 3.42 Hz, 1H, H-3), 5.18 (d, *J* = 7.98 Hz, 1H, H-1), 4.15 (m, 1H, H-5), 3.87 (m, 2H, H-6,6′), 1.01 (s, 9H, *t*-Bu H); ^13^C NMR (150 MHz, CDCl_3_): *δ* = 165.8, 165.6, 165.4, 151.6, 116–136 (Ar–C), 101.5, 74.5, 72.1, 70.0, 68.0, 61.8, 26.86, 19.21. ESI-MS data: [M + H] *m*/*z*: calcd for C_49_H_46_SiNaO_10_, 845.3; found, 845.5.

### 4-Hydroxyphenyl-2,3,4-tri-*O*-benzoyl-6-*O-t*-butyldiphenylsilyl-β-d-galactopyranosyl-(1→6)-2,3,4-tri-*O*-benzyl-β-d-galactopyranoside (9a)

Compound 9 (150 mg) was dissolved in dry THF (10 mL) and procedure mentioned above was followed. Product 9a: white solid (29 mg, 61%). *R*_f_: 0.28 (40% EtOAc/hexane); ^1^H NMR (600 MHz, CDCl_3_): *δ* 7.16–7.65 (Ar–H), 6.98 (d, *J* = 8.9 Hz, 2H, hydroquinone *para*-H), 6.82 (d, *J* = 8.9 Hz, 2H, hydroquinone *ortho*-H), 5.94 (m, 2H, H-4, H′-2), 5.82 (dd, *J* = 0.67, 3.48 Hz, 1H, H′-4), 5.63 (t, *J* = 2.52 Hz, 1H, H-2), 5.50 (m, 2H, H-3, H′-3), 5.14 (d, *J* = 7.98 Hz, 1H, H′-1), 4.88 (d, *J* = 7.98 Hz, 1H, H-1), 4.10 (m, 1H, H′-5), 3.98 (m, 2H, H′-6,6′), 3.83 (m, 1H, H-5), 3.67 (m, 2H, H-6,6′), 0.95 (s, 9H, *t*-Bu H); ^13^C NMR (150 MHz, CDCl_3_): *δ* = 166.3, 165.8, 165.6, 165.4, 152.4, 150.8, 116–136 (Ar–C), 101.1, 100.6, 75.3, 73.9, 72.2, 71.7, 70.1, 69.7, 69.0, 67.9, 61.06, 26.7, 19.09; HRMS data: [M + Na] *m*/*z*: calcd for C_76_H_68_O_18_SiNa, 1319.4072; found, 1319.4023.

### 4-Hydroxyphenyl-2,3,4-tri-*O*-benzoyl-6-*O-t*-butyldiphenylsilyl-β-d-galactopyranosyl-(1→6)-2,3,4-tri-*O*-benzyl-β-d-galactopyranosyl-(1→6)-2,3,4-tri-*O*-benzyl-β-d-galactopyranoside (11a)

Compound 11 (450 mg) was dissolved in dry THF (10 mL) and procedure mentioned above was followed. Product 11a: white solid (85 mg, 48%). *R*_f_: 0.12 (40% EtOAc/hexane); ^1^H NMR (600 MHz, CDCl_3_): 7.00–8.15 (Ar–H), 6.95 (d, *J* = 8.9 Hz, 2H, hydroquinone *para*-H), 6.78 (d, *J* = 8.9 Hz, 2H, hydroquinone *ortho*-H), 5.98 (dd, *J* = 6.8, 4.4 Hz, 1H, H′-4), 5.93 (dd, *J* = 10.4, 7.9 Hz, 1H, H′′-2), 5.84 (dd, *J* = 9.0, 3.6 Hz, 2H, H-4, H′′-4), 5.63–5.53 (m, 3H, H-2, H′-2, H′-3), 5.50 (dd, *J* = 10.4, 3.5 Hz, 1H, H′′-3), 5.42 (dd, *J* = 10.4, 3.5 Hz, 1H, H-3), 5.13 (d, *J* = 8.0 Hz, 1H, H′′-1), 4.81 (d, *J* = 8.0 Hz, 1H, H′-2), 4.56 (d, *J* = 7.4 Hz, 1H, H-1), 4.07 (q, *J* = 6.1 Hz, 1H, H-5), 3.96–3.91 (m, 2H, H-6,6′), 3.90–3.87 (m, 2H, H′′-5, H′′-6), 3.83 (dd, *J* = 8.6, 6.4 Hz, 1H, H′-5), 3.60–3.52 (m, 1H, H′′-6′), 3.33–3.26 (m, 2H, H′-6, 6′), 0.95 (s, 9H, *t*-Bu H); ^13^C NMR (150 MHz, CDCl_3_): 165.6, 152.6, 150.6, 116–136 (Ar–C), 101.3, 100.6, 100.4, 74.9, 73.5, 72.3, 72.0, 71.9, 71.7, 70.3, 69.7, 68.9, 67.7, 67.5, 60.0, 26.7, 18.9; HRMS data: [M + Na] *m*/*z*: calcd for C_103_H_90_O_26_SiNa, 1793.5387; found, 1793.5365.

### 4-((4-Carboxylate-2-nitrobenzyl)oxy)phenyl-2,3,4-tri-*O*-benzoyl-6-*O-t*-butyldiphenylsilyl β-d-glucopyranoside (12)

The acceptor 4 (0.12 g, 0.35 mmol) and thiglycoside donor B (0.25 g, 0.32 mmol) were dissolved in dry DCM (10 mL) followed by addition of 4 Å molecular sieves and stirred for 30 minutes under N_2_. NIS (0.11 g, 0.48 mmol) and TfOH (50 μL) were added to the reaction at −40 °C. The solution was then allowed to warm up to room temperature. The *t*-butyl group on linker was cleaved upon warming to room temperature and standing over 12 h under these acidic conditions. The reaction was diluted with DCM (30 mL) and filtered. The filtrate was washed with aq. sodium thiosulfate (2 × 30 mL), aq. sodium bicarbonate (2 × 30 mL) and brine (2 × 30 mL). Organic layer was collected, dried over anhydrous Na_2_SO_4_ and subjected to flash column chromatography (CHCl_3_ : EtOH; 9 : 1) on silica gel to afford compound 12 as yellow glassy liquid. *R*_f_: 0.42 (10% EtOH/CHCl_3_); ^1^H NMR (600 MHz, CDCl_3_): *δ* 7.1–7.5 (aromatic-H), 7.07 (d, *J* = 9.1 Hz, 2H, hydroquinone *para*-H), 6.85 (d, *J* = 9.1 Hz, 2H, hydroquinone *ortho*-H), 5.95 (t, *J* = 9.6 Hz, 1H, H-3), 5.78 (dd, *J* = 9.7, 7.9 Hz, 1H, H-2), 5.68 (t, *J* = 9.8 Hz, 1H, H-4), 5.31 (d, *J* = 7.8 Hz, 1H, H-1), 4.03 (ddd, *J* = 9.9, 5.6, 2.2 Hz, 1H, H-5), 3.95–3.86 (m, 2H, H-6,6′), 1.05 (s, 9H, - *t*-Bu H); ^13^C NMR (150 MHz, CDCl_3_): *δ* 166.3, 165.5, 165.3, 153.9, 152.2, 126–146 (Ar–C), 115.8, 119.2, 100.7, 75.8, 73.4, 72.1, 69.3, 67.4, 62.8, 26.7, 19.3; HRMS data: [M + Na] *m*/*z*: calcd for C_57_H_51_NO_14_SiNa, 1024.2971; found, 1024.2978.

### Polymer loading and glycosyaltions

The monosaccharide building block 12 (125 mg, 0.12 mmol) was loaded on to the polymer pHEMA (165 mg, 1.20 mmol) using EDC-DMAP-mediated coupling as described earlier. The loading capacity was calculated to be 0.2 mmol g^−1^ in this case using TMS as an internal standard. The de-protection and glycosyaltion cycles were followed as mentioned before and glycosylation yields were calculated by cleaving small amount of polymer intermediates using the photo-activation procedure.

### 4-Hydroxyphenyl-2-*O*-levulinate-3,4-di-*O*-benzyl-α-l-rhamnopyranosyl-(1→6)-2,3,4-tri-*O*-benzyl-β-d-glucopyranoside (15a)

Disaccharide bound pHEMA 15 (125 mg) was dissolved in dry THF and the procedure mentioned for photo-activated cleavage was followed. Product 15a: colourless oil (16 mg, 64%). *R*_f_: 0.30 (40% EtOAc/hexane); ^1^H NMR (600 MHz, CDCl_3_): *δ* 7.00–8.15 (Ar–H), 6.85 (d, *J* = 9.0 Hz, 2H, hydroquinone *para*-H), 6.67 (d, *J* = 9.0 Hz, 2H, hydroquinone *ortho*-H), 5.92 (t, *J* = 9.5 Hz, 1H, H-3), 5.71 (dd, *J* = 9.6, 7.4 Hz, 1H, H-2), 5.48 (t, *J* = 9.8 Hz, 1H, H-4), 5.44 (t, *J* = 10.8 Hz, 1H, H′-2), 5.25 (d, *J* = 7.4 Hz, 1H, H-1), 4.87 (d, *J* = 3.9 Hz, 1H, H′-1), 4.65 (d, *J* = 11.0 Hz, 1H, Bn-H), 4.58 (d, *J* = 10.8 Hz, 1H, Bn-H), 4.42 (d, *J* = 11.0 Hz, 1H, Bn-H), 4.09 (ddd, *J* = 9.9, 7.9, 2.0 Hz, 1H, H-5), 3.86 (ddd, *J* = 12.7, 10.8, 5.7 Hz, 2H, H-6, H′-3), 3.70 (dd, *J* = 12.2, 2.1 Hz, 2H, H-6′, H′-5), 3.34 (dd, *J* = 19.6, 10.2 Hz, 1H, H′-4), 2.90–2.76 (m, 2H, Lev-CH_2_), 2.62–2.52 (m, 2H, Lev-CH_2_), 2.25 (s, 3H, Lev-CH_3_), 1.28 (d, *J* = 6.2 Hz, 3H, Rha-CH_3_); ^13^C NMR (150 MHz, CDCl_3_): *δ* 209.1, 172.1, 165.9, 165.3, 115–155 (Ar–C), 100.2, 98.2, 79.9, 78.4, 75.5, 75.4, 72.9, 72.2, 71.8, 69.6, 68.9, 68.2, 65.7, 37.9, 30.5, 28.3, 18.2; HRMS data: [M + Na] *m*/*z*: calcd for C_58_H_56_O_16_Na, 1031.3466; found, 1.31.3442.

### 4-Hydroxyphenyl-2,3,4-tri-*O*-benzoyl-6-*O-t*-butyldiphenylsilyl-β-d-glucopyranosyl-(1→2)-3,4-di-*O*-benzyl-α-l-rhamnopyranosyl-(1→6)-2,3,4-tri-*O*-benzyl-β-d-glucopyranoside (17a)

Trisaccharide-bound pHEMA 17 (120 mg) was dissolved in dry THF (10 mL) and purged with N_2_. The reaction mixture was exposed to a light of 364 nm wavelength and stirred at RT in a photochemical reactor for 2 h. The complete cleavage of oligosaccharide was monitored by TLC (HEX : EtOAc, 7 : 3). The reaction mixture was evaporated and dissolved in EtOAc (20 mL). The free polymer was then precipitated out of the solution using cold methanol (100 mL) and filtered off. The filtrate was evaporated and dried under high-vacuum. The filtrate was dissolved in dry DCM (5 mL) followed by addition of acetic anhydride (1 mL) and pyridine (1 mL). The reaction mixture was stirred overnight and diluted with DCM (20 mL). The reaction mixture was washed with 1 N HCl (3 × 30 mL). The organic layer was collected, evaporated and subjected to column chromatography (Hex : EtOAc, 7 : 3) to afford final compound 17a as a white solid (14 mg, 39%). *R*_f_: 0.48 (40% EtOAc/hexane); ^1^H NMR (600 MHz, CDCl_3_) *δ* 7.00–8.15 (Ar–H), 6.97 (d, *J* = 9.1 Hz, 2H, hydroquinone *para*-H), 6.89 (d, *J* = 9.1 Hz, 2H, hydroquinone *ortho*-H), 5.92 (t, *J* = 9.6 Hz, 1H, H-3), 5.72 (dd, *J* = 9.7, 7.8 Hz, 1H, H-2), 5.57 (t, *J* = 9.7 Hz, 1H, H-4), 5.42 (s, 2H, Bn H), 5.39 (d, *J* = 3.3 Hz, 1H, H′-2), 5.25 (d, *J* = 7.8 Hz, 1H, H-1), 4.88 (d, *J* = 10.9 Hz, 1H, H′′-1), 4.74 (d, *J* = 3.5 Hz, 1H, H′-1), 4.59 (dd, *J* = 16.4, 10.9 Hz, 2H, H′′-2, H′′-4), 4.35 (d, *J* = 10.9 Hz, 1H, H′′-3), 4.05 (m, 2H), 3.89–3.81 (m, 2H, H′-3, H′-5), 3.78–3.72 (m, 3H, H-5, H-6,6′), 3.38 (t, *J* = 9.4 Hz, 1H, H′-4), 2.11 (s, 3H, Ac–H), 1.26 (s, 9H, *t*-Bu H), 1.24 (d, *J* = 6.2 Hz, 3H, Rha-CH_3_); ^13^C NMR (150 MHz, CDCl_3_): *δ* 167.2, 165.9, 165.3, 165.1, 152.8, 149.9, 115–136 (Ar–C), 99.9, 99.8, 99.6, 82.8, 79.9, 75.5, 75.2, 73.8, 72.8, 71.94, 69.7, 69.4, 67.8, 67.4, 65.8, 29.9, 28.3, 26.8, 19.4, 18.0; ESI-MS data: [M + Na] *m*/*z*: calcd for C_98_H_92_O_23_SiNa, 1687.6; found, 1687.7.

## Conflicts of interest

There are no conflicts to declare.

## Supplementary Material

RA-008-C8RA08252A-s001
