# Supplementary material for: Oligosaccharide synthesis on soluble high-molecular weight pHEMA using a photo-cleavable linker
Source: RSC Adv. 2018 Dec 12;8(72):41612–9. doi: 10.1039/c8ra08252a (PMC6602538; doi:10.1039/c8ra08252a)

Electronic Supplementary Material (ESI) for RSC Advances.
This journal is © The Royal Society of Chemistry 2018

Oligosaccharide Synthesis on Soluble High-Molecular Weight Polymer pHEMA Using a Photo-Cleavable Linker

Abhishek Vartak, Sandeep Thanna, Kyle Meyer, Miranda Dermanelian, Steven J. Sucheck*

Department of Chemistry and Biochemistry, University of Toledo. 2801 W. Bancroft Street, Toledo, Ohio 43606, USA.

Supplementary Information

Table of contents

1. ^{1}H NMR of pHEMA bound monosaccharide and their comparison 1

2. Polymer percentage recovery comparison between pHEMA and Boltron H40 2

3. MS and NMR data of novel compounds 3-47

Percentage loading calculations:

Polymer bound monosaccharide **7** (20 mg) was dissolved in 0.8 mL of $CDCl_3$ containing 1% TMS V/V. The TMS peak was integrated as 1 proton at chemical shift value 0.0 and remaining saccharide peaks were integrated accordingly.

The ratio between analyte and internal standard:

$r_{A/IS}$ = (0.007/1) / (1/12) = 0.084

The sample contains 5.2 mg of TMS:

n_{IS} = 5.2 / 88 = 0.059 mmol.

$n_A = n_{IS} * r_{A/IS}$ = 0.005 mmol / 20 mg of polymer = 0.25 mmol/g of polymer.

Figure S1. ^{1}H NMR of pHEMA bound monosaccharide

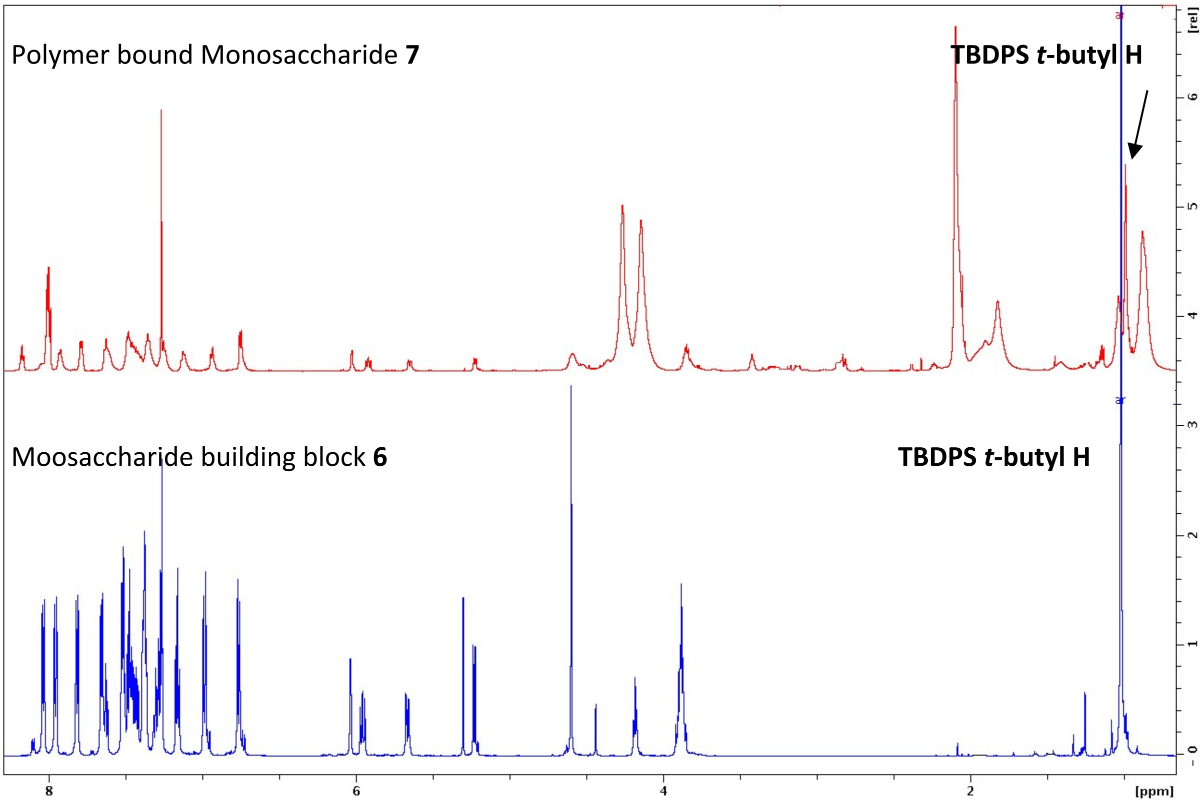

Figure S2. ^{1}H NMR of pHEMA bound oligosaccharide **7** and **8** (TBDPS de-protection)

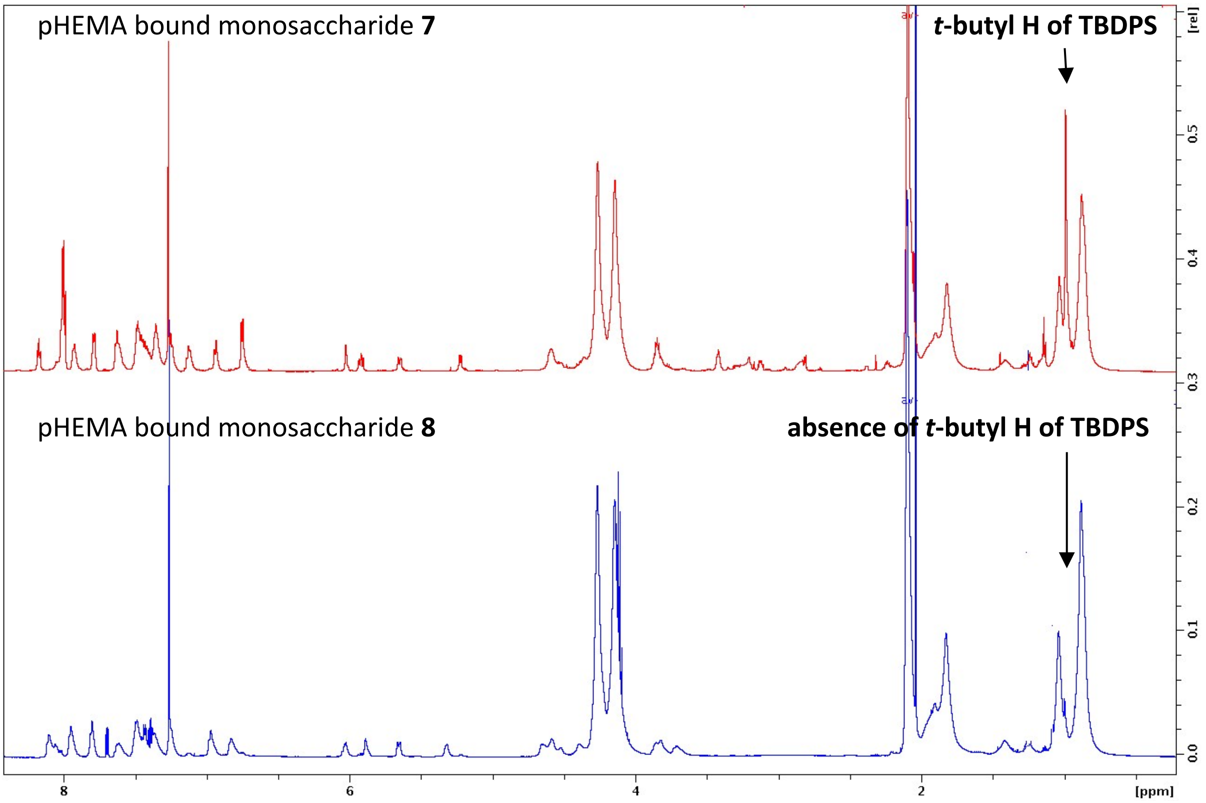

Polymer percentage recovery comparison between pHEMA and Boltron H40

To compare pHEMA recovery with other polymer support, we decided to use hyper branched Boltron H40 polymer for model tri-galactoside synthesis. Both polymers were recovered using precipitation method. The average recovery of pHEMA over 2 cycles of de-protections and glycosylations was 91% compared to 64% of Boltron H40.

Polymer bound product	pHEMA Recovery (%)	Boltron H40 Recovery (%)
Loading and Capping **7**	80	58
1st De-protection **8**	94	71
Glycosylation **9**	90	55
2nd De-protection **10**	92	64
Glycosylation **11**	89	65

Table S1. Percentage recovery comparison between pHEMA and Boltron H40 using Precipitation method.

HRMS data of ethyl-2,3,4-tri-*O*-benzoyl-6-*O*-*t*-butyldiphenylsilyl 1-thio-*β*-D-galactopyranoside (A)

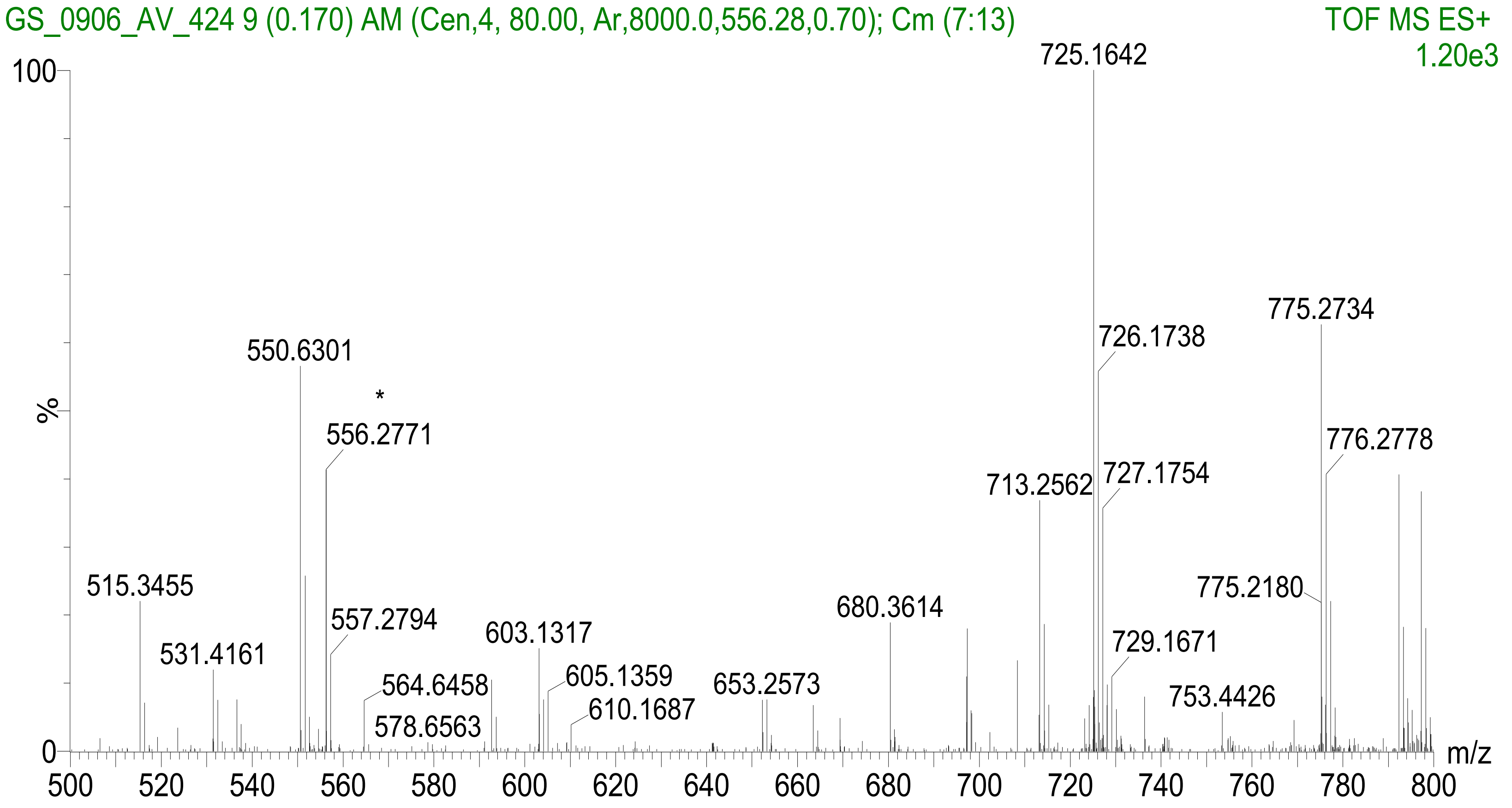

Mass accuracy = ((775.2761-775.2734)/ 775.2761)* 106 = 3.5 ppm

^{1}H NMR of ethyl-2,3,4-tri-*O*-benzoyl-6-*O*-*t*-butyldiphenylsilyl 1-thio-*β*-D-galactopyranoside (A)

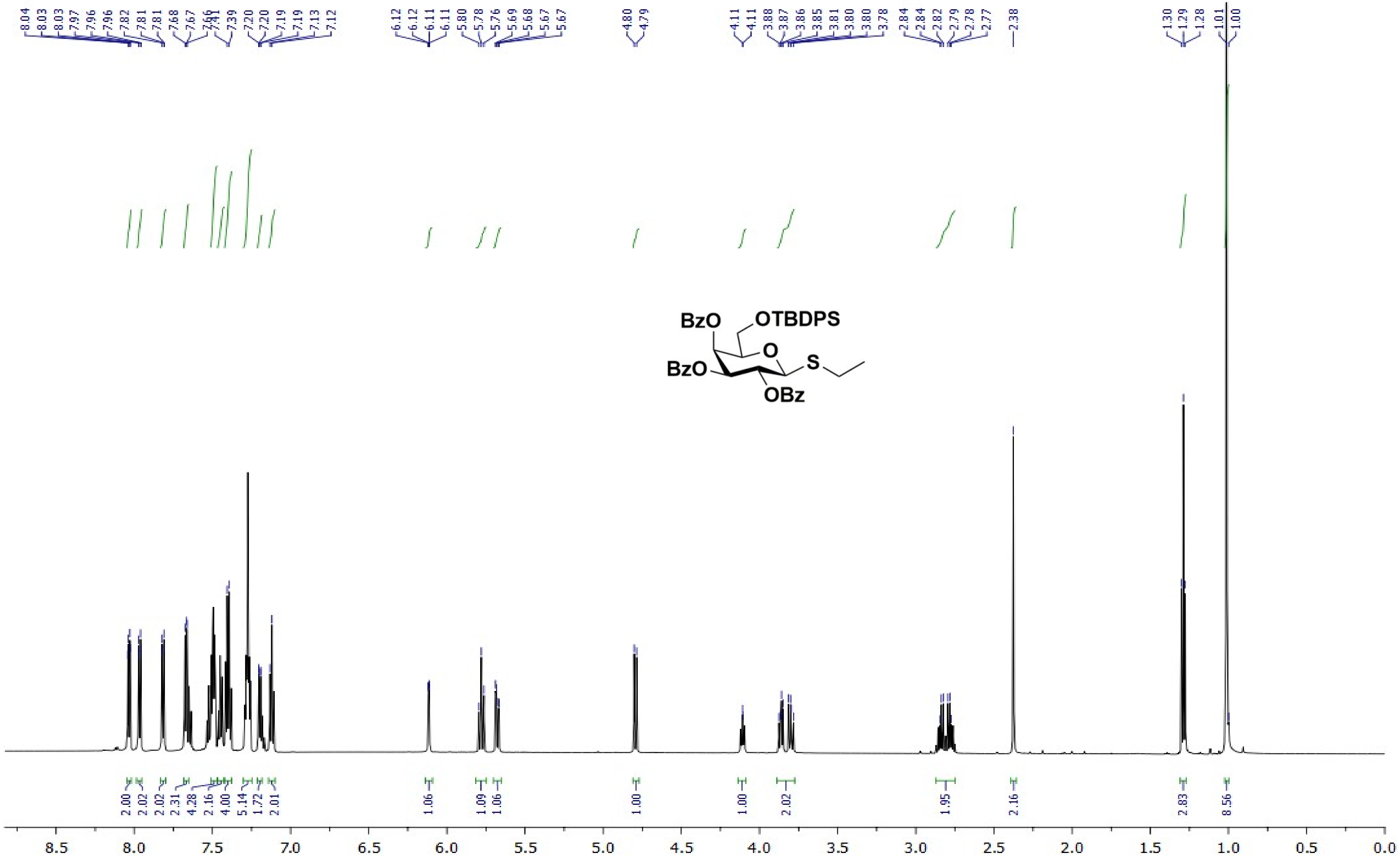

^{13}C NMR of Ethyl-2,3,4-tri-*O*-benzoyl-6-*O*-*t*-butyldiphenylsilyl 1-thio-*β*-D-galactopyranoside (A)

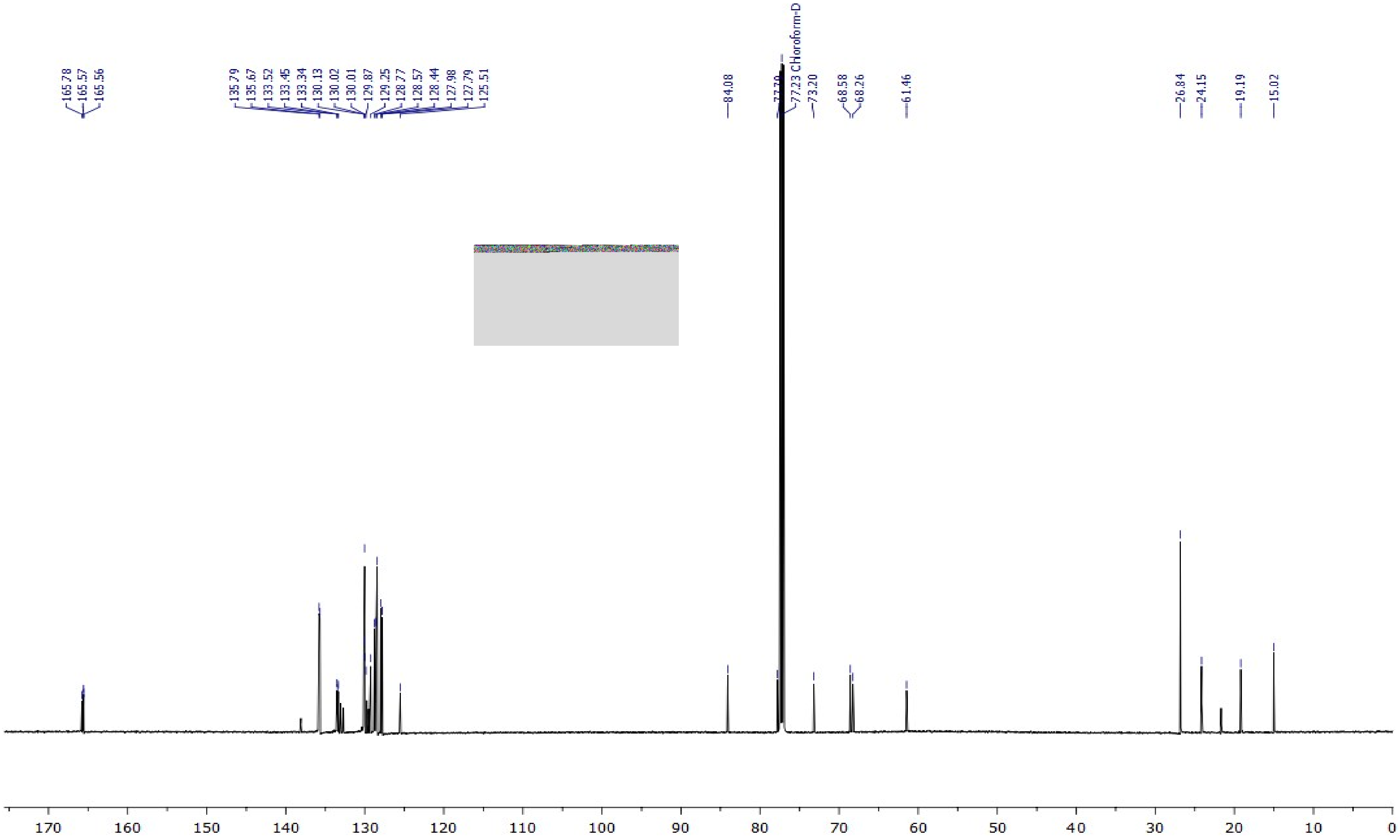

HRMS data of *t*-butyl 4-((4-((*t*-butyldiphenylsilyl)oxy)phenoxy)methyl)-3-nitrobenzoate (3)

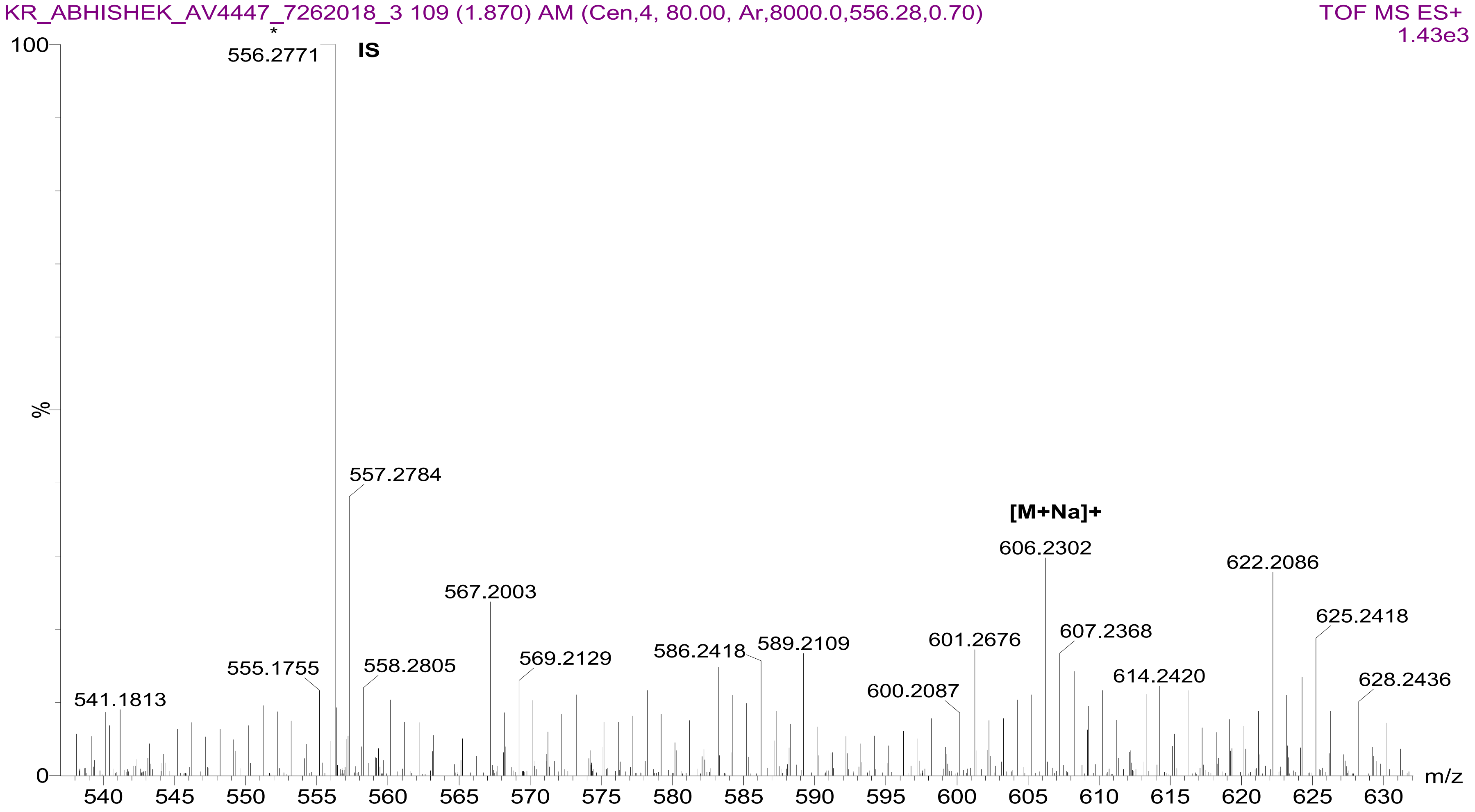

Mass accuracy = ((606.2288-606.2302)/606.2288)* 106 = 2.3 ppm

^{1}H NMR of *t*-butyl 4-((4-((*t*-butyldiphenylsilyl)oxy)phenoxy)methyl)-3-nitrobenzoate (3)

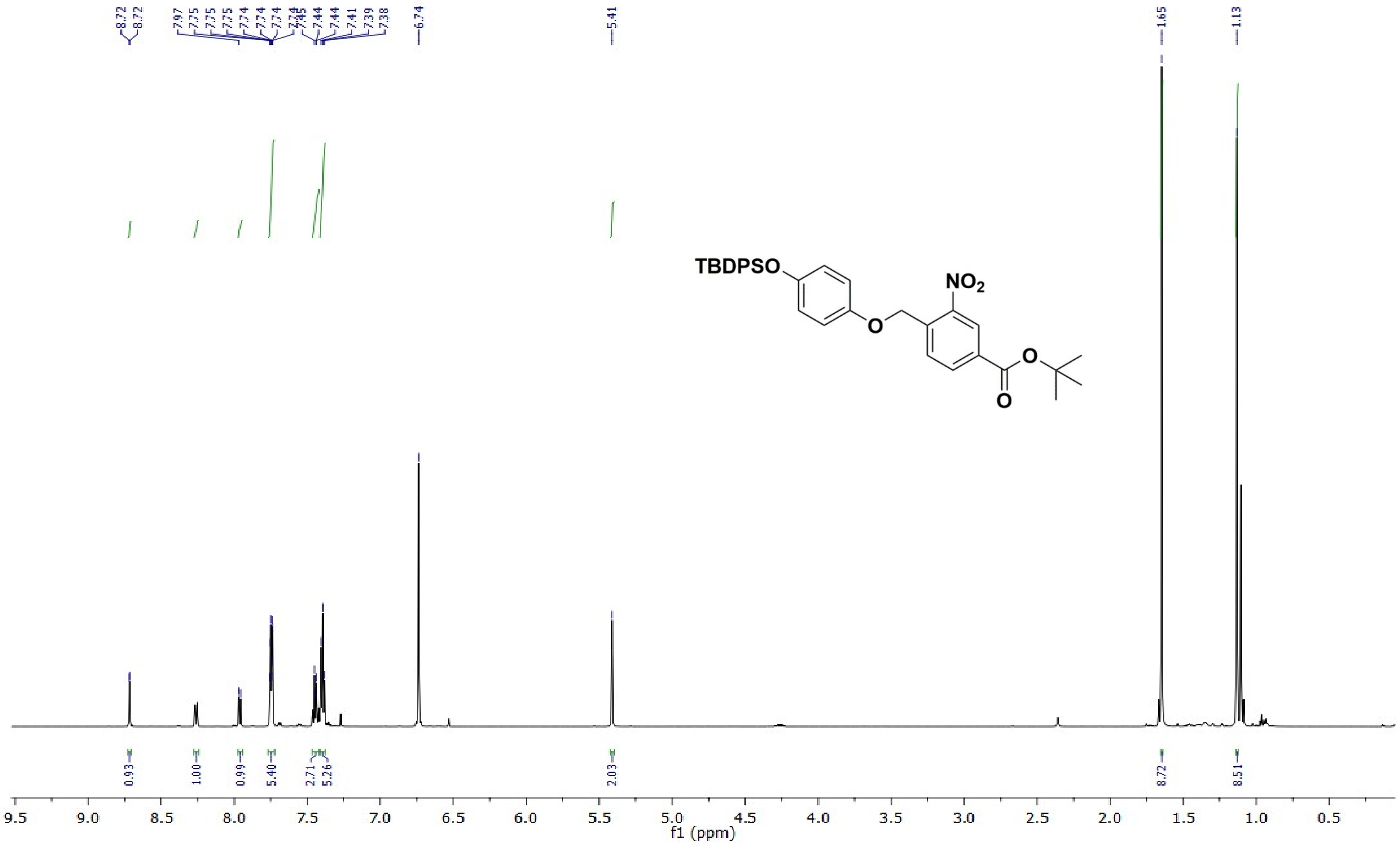

^{13}C NMR of *t*-butyl 4-((4-((*t*-butyldiphenylsilyl)oxy)phenoxy)methyl)-3-nitrobenzoate (3)

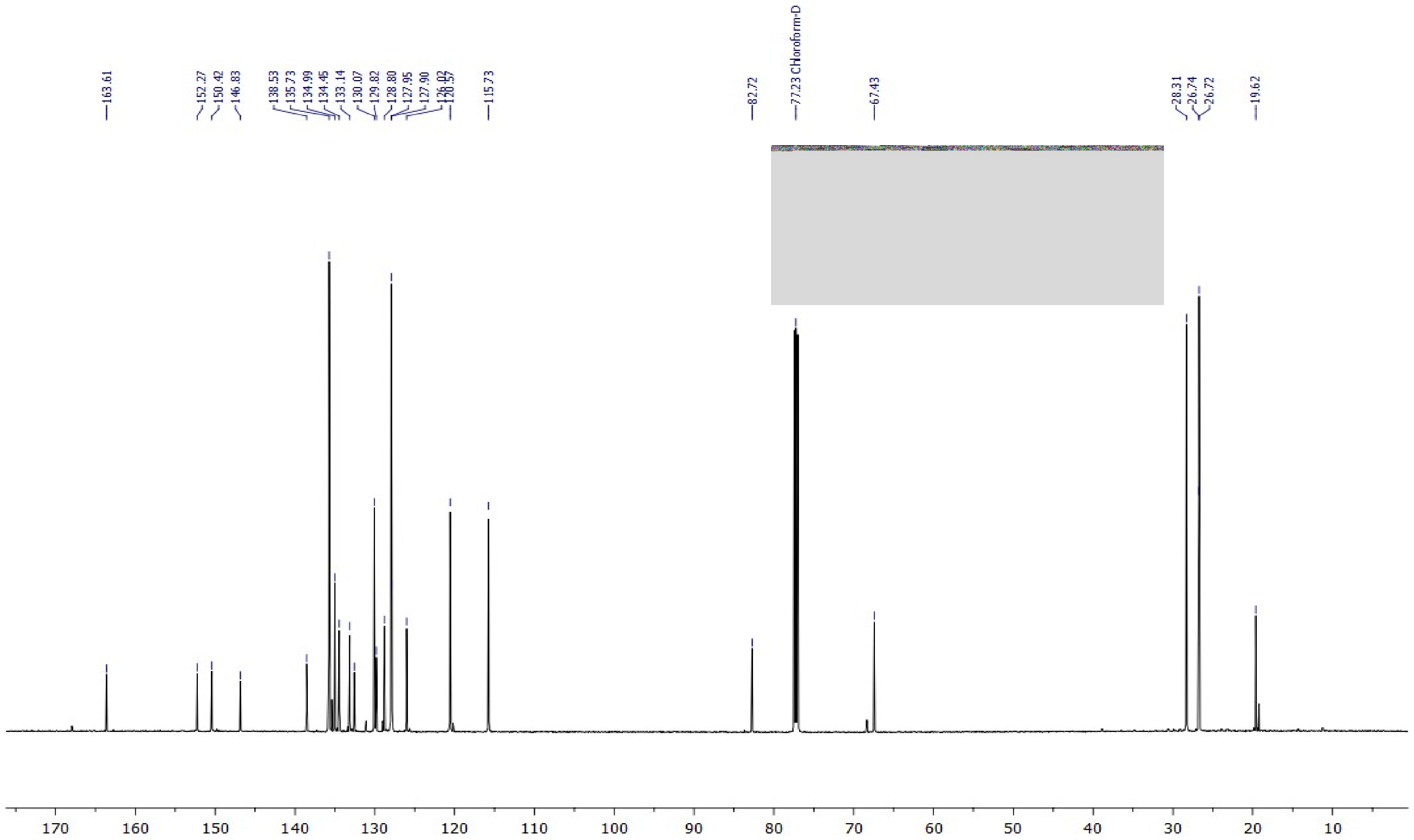

HRMS data of *t*-butyl 4-((4-hydroxyphenoxy)methyl)-3-nitrobenzoate (4)

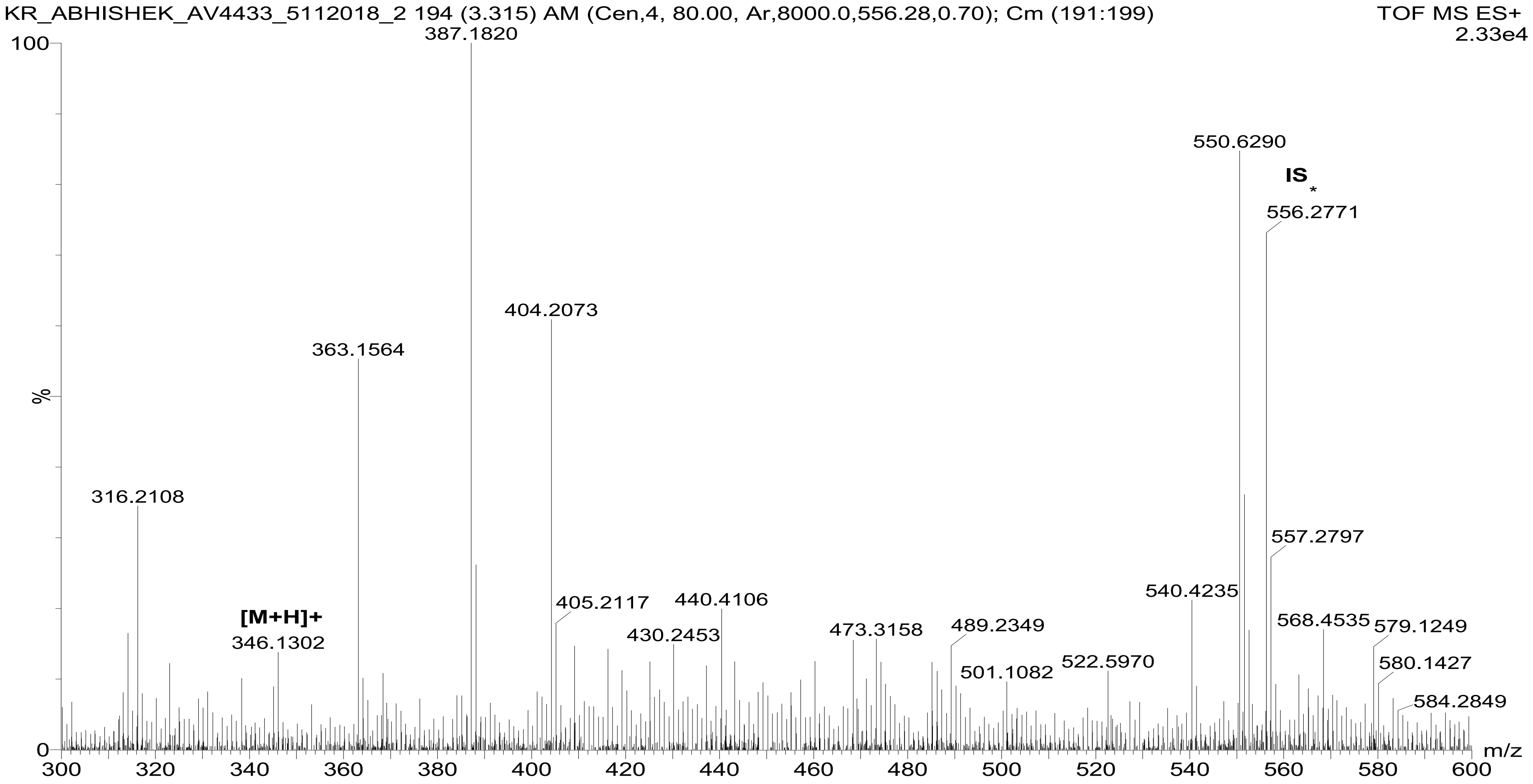

Mass accuracy = ((346.1291-346.1302)/346.1291)* 106 = 3.2 ppm

^{1}H NMR of *t*-butyl 4-((4-hydroxyphenoxy)methyl)-3-nitrobenzoate (4)

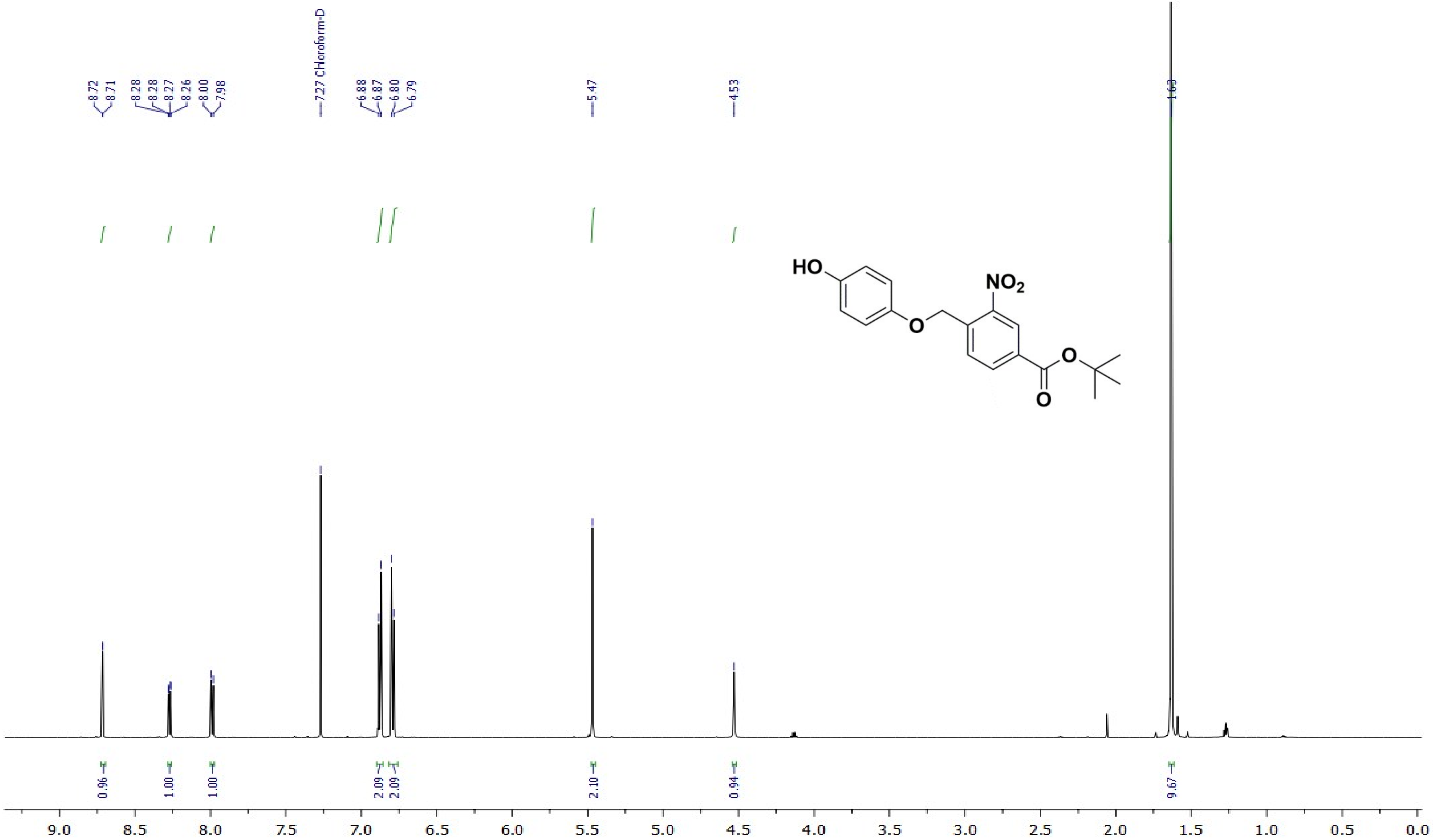

^{13}C NMR of *t*-butyl 4-((4-hydroxyphenoxy)methyl)-3-nitrobenzoate (4)

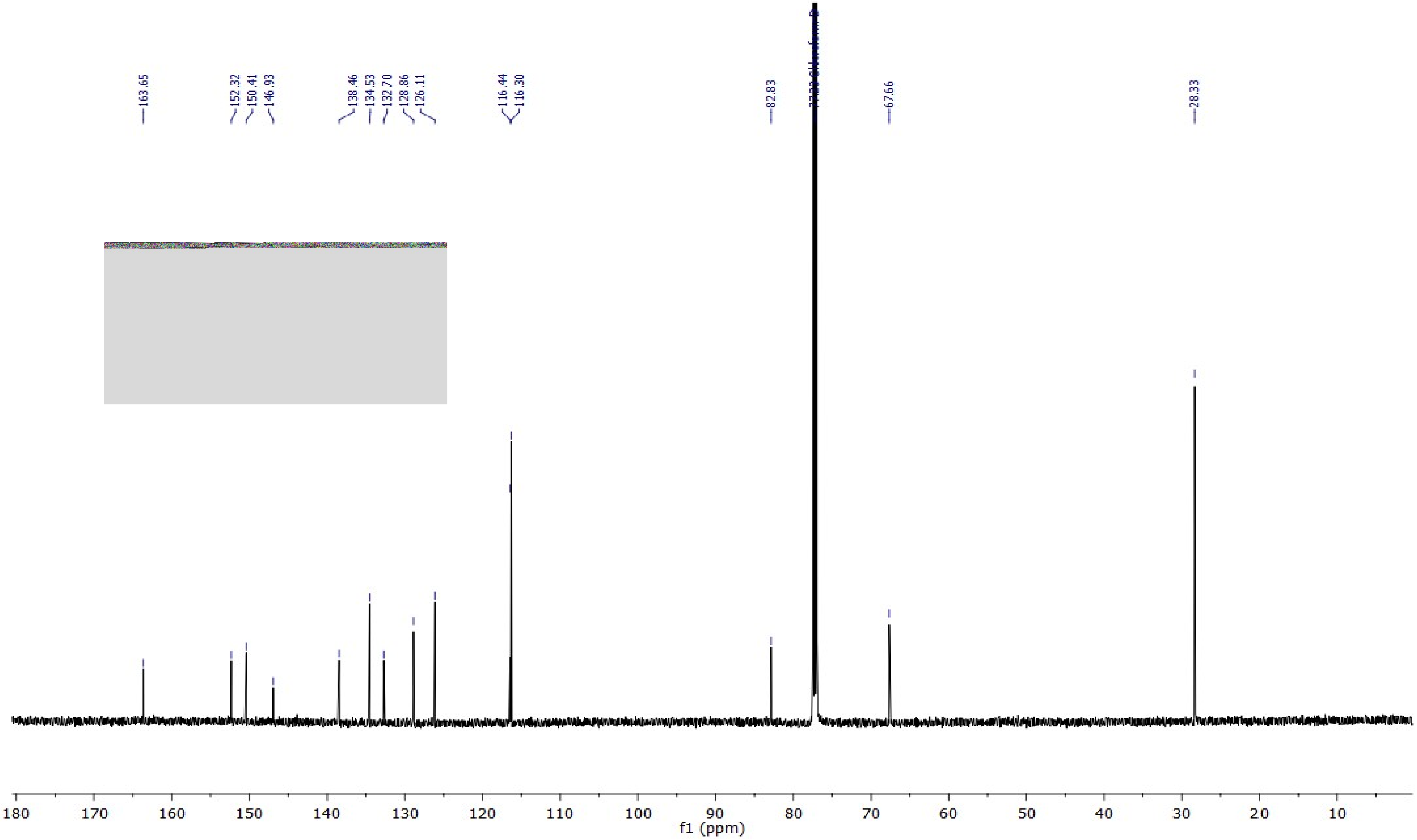

ESI-MS data of **4-((4-(*t*-butoxycarbonyl)-2-nitrobenzyl)oxy)phenyl-2,3,4-tri-*O*-benzoyl-6-*O*-*t*-butyldiphenylsilyl *β*-D-galactopyranoside (5)**

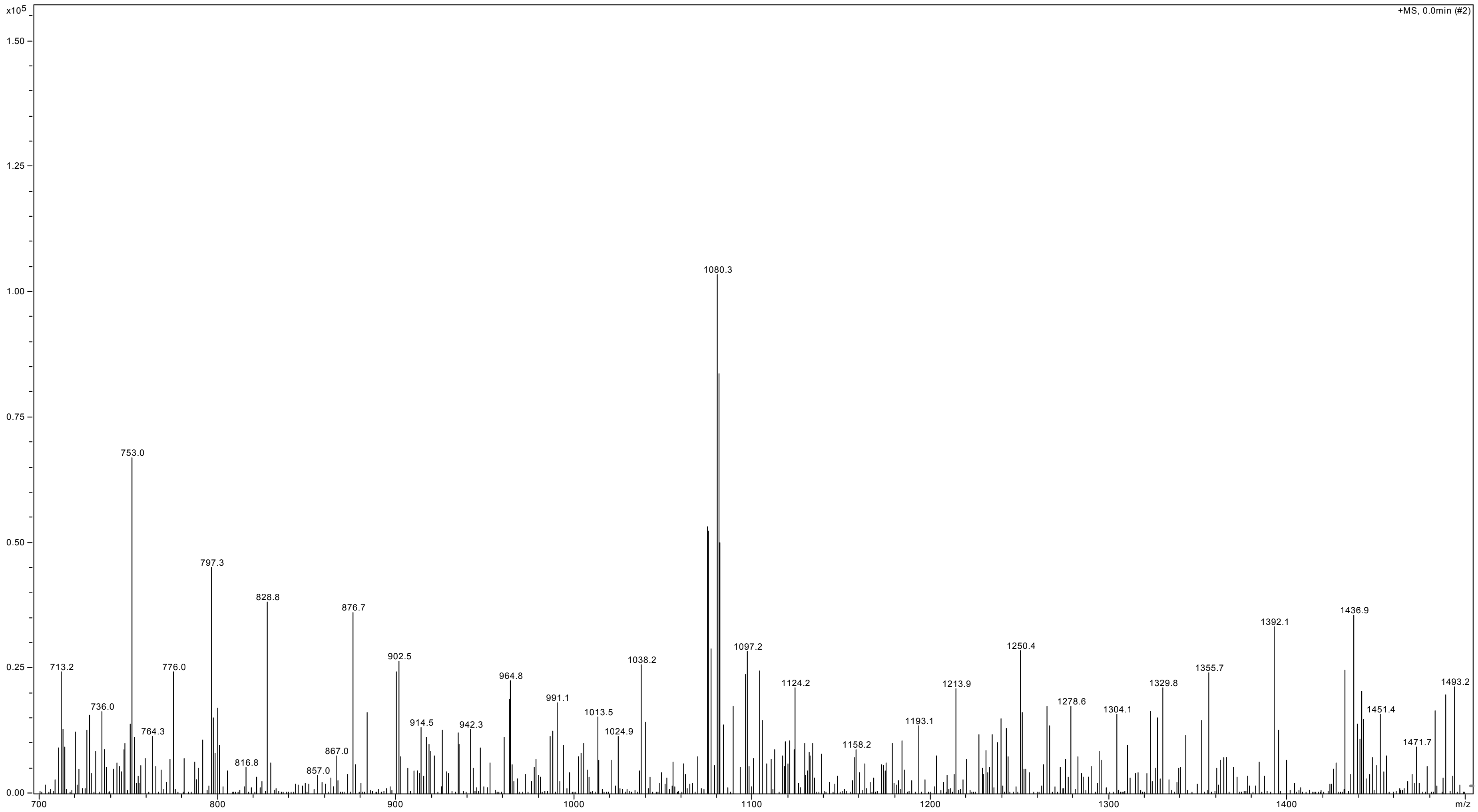

^{1}H NMR of 4-((4-(*t*-butoxycarbonyl)-2-nitrobenzyl)oxy)phenyl-2,3,4-tri-*O*-benzoyl-6-*O*-*t*-butyldiphenylsilyl *β*-D-galactopyranoside (5)

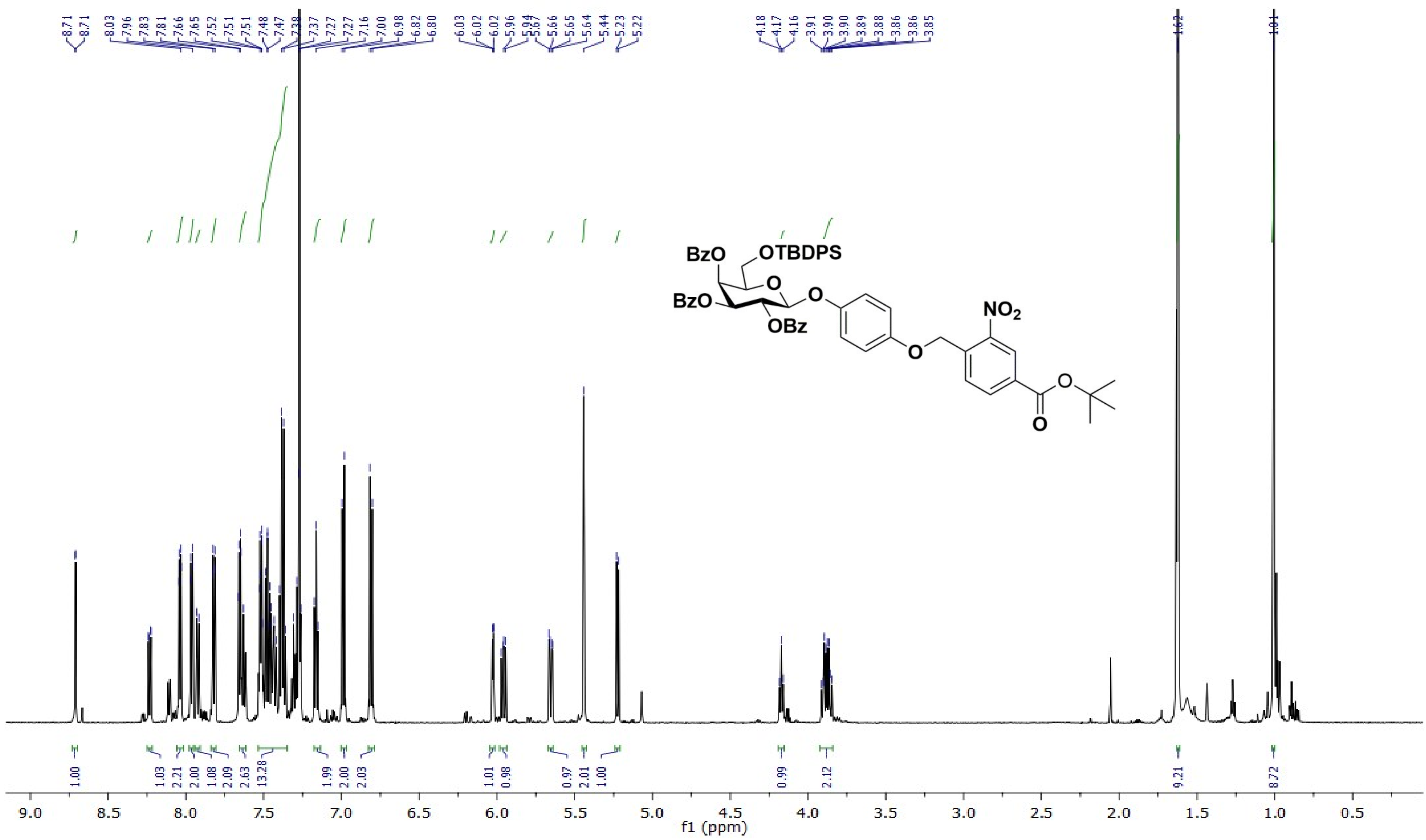

^{13}C NMR of 4-((4-(*t*-butoxycarbonyl)-2-nitrobenzyl)oxy)phenyl-2,3,4-tri-*O*-benzoyl-6-*O*-*t*-butyldiphenylsilyl *β*-D-galactopyranoside (5)

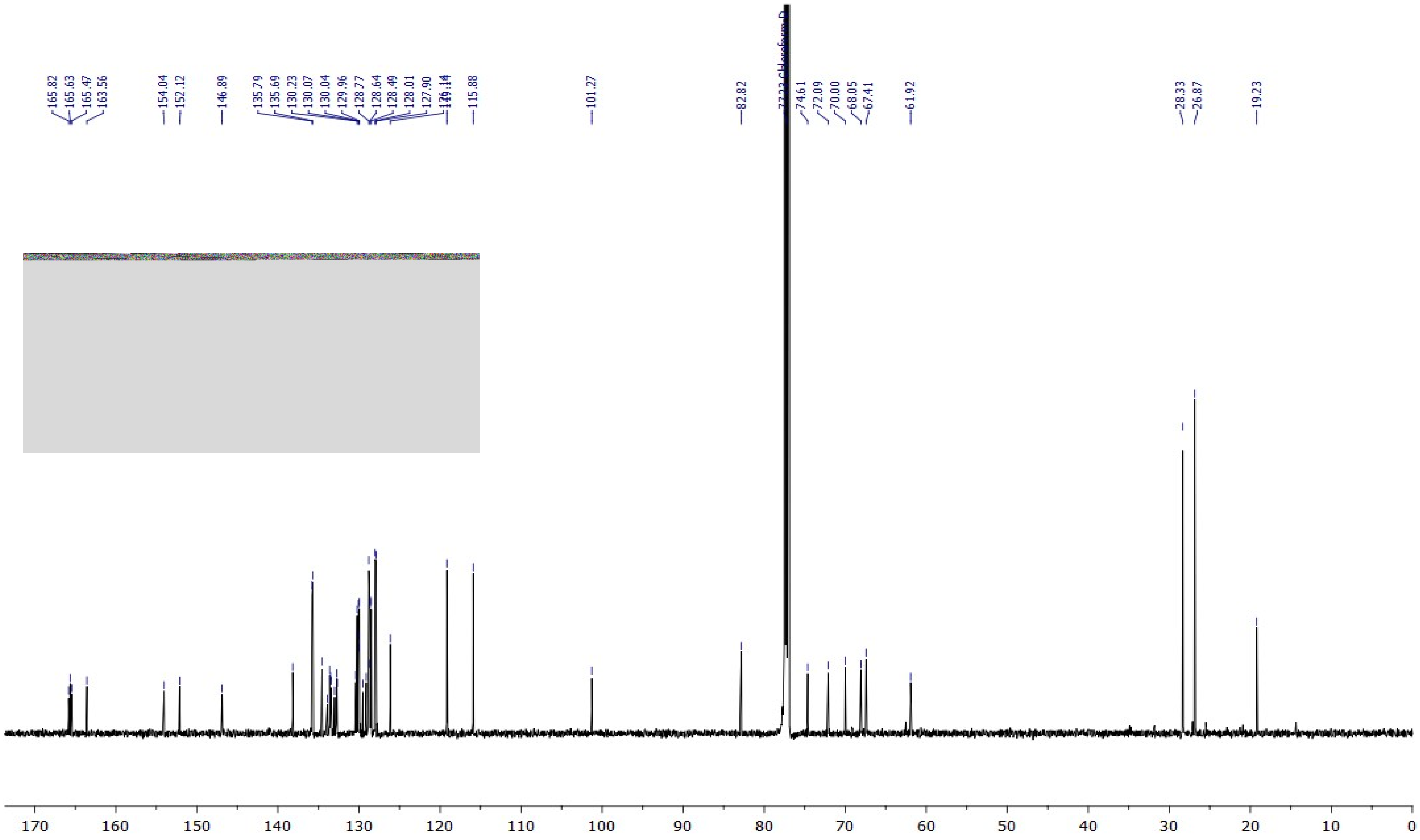

HRMS data of 4-((4-carboxylate-2-nitrobenzyl)oxy)phenyl-2,3,4-tri-*O*-benzoyl-6-*O*-*t*-butyldiphenylsilyl *β*-D-galactopyranoside (6)

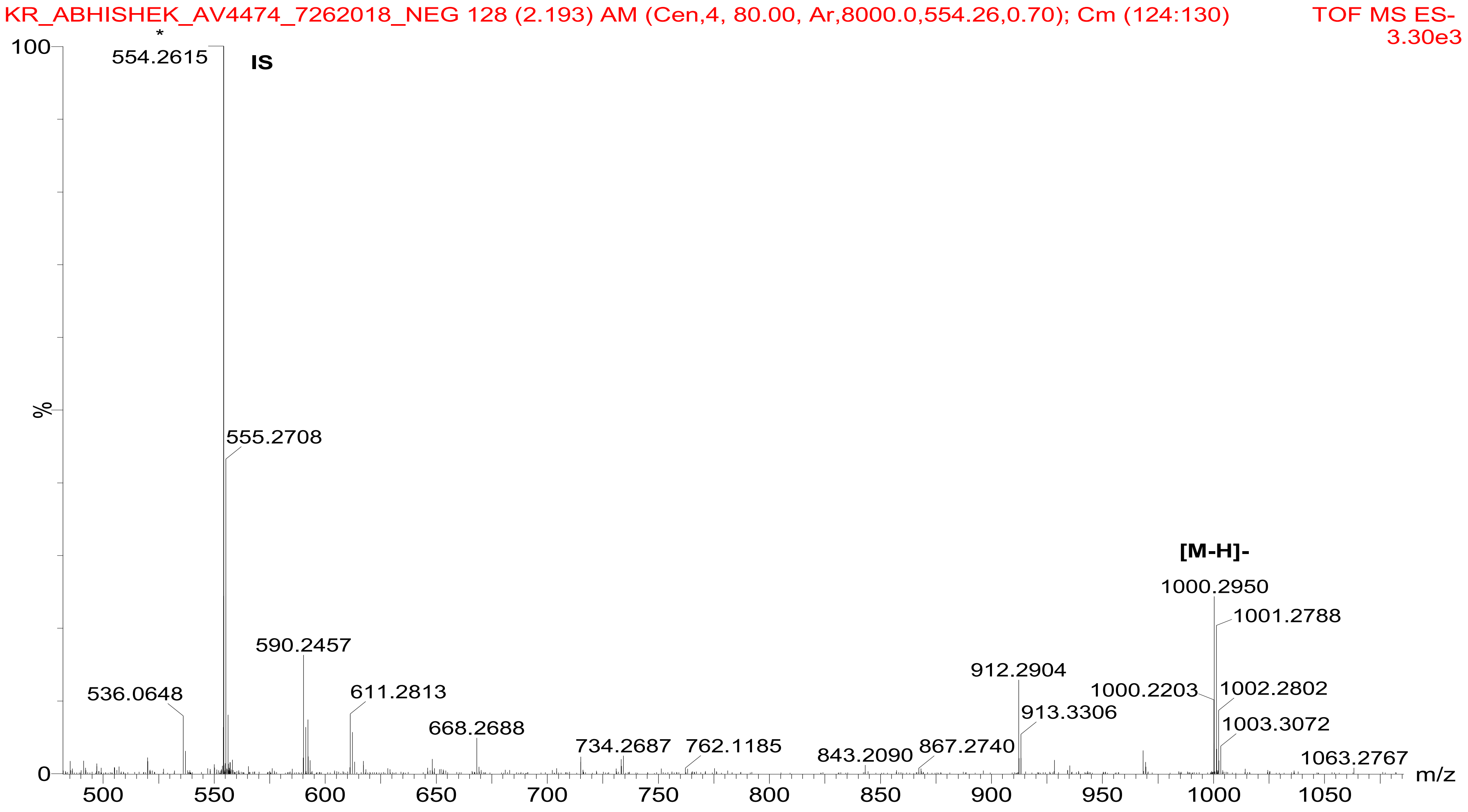

Mass accuracy = ((1000.3000-1000.2950)/1000.3000)* 106 = 5.0 ppm

^{1}H NMR of 4-((4-carboxylate-2-nitrobenzyl)oxy)phenyl-2,3,4-tri-*O*-benzoyl-6-*O*-*t*-butyldiphenylsilyl *β*-D-galactopyranoside (6)

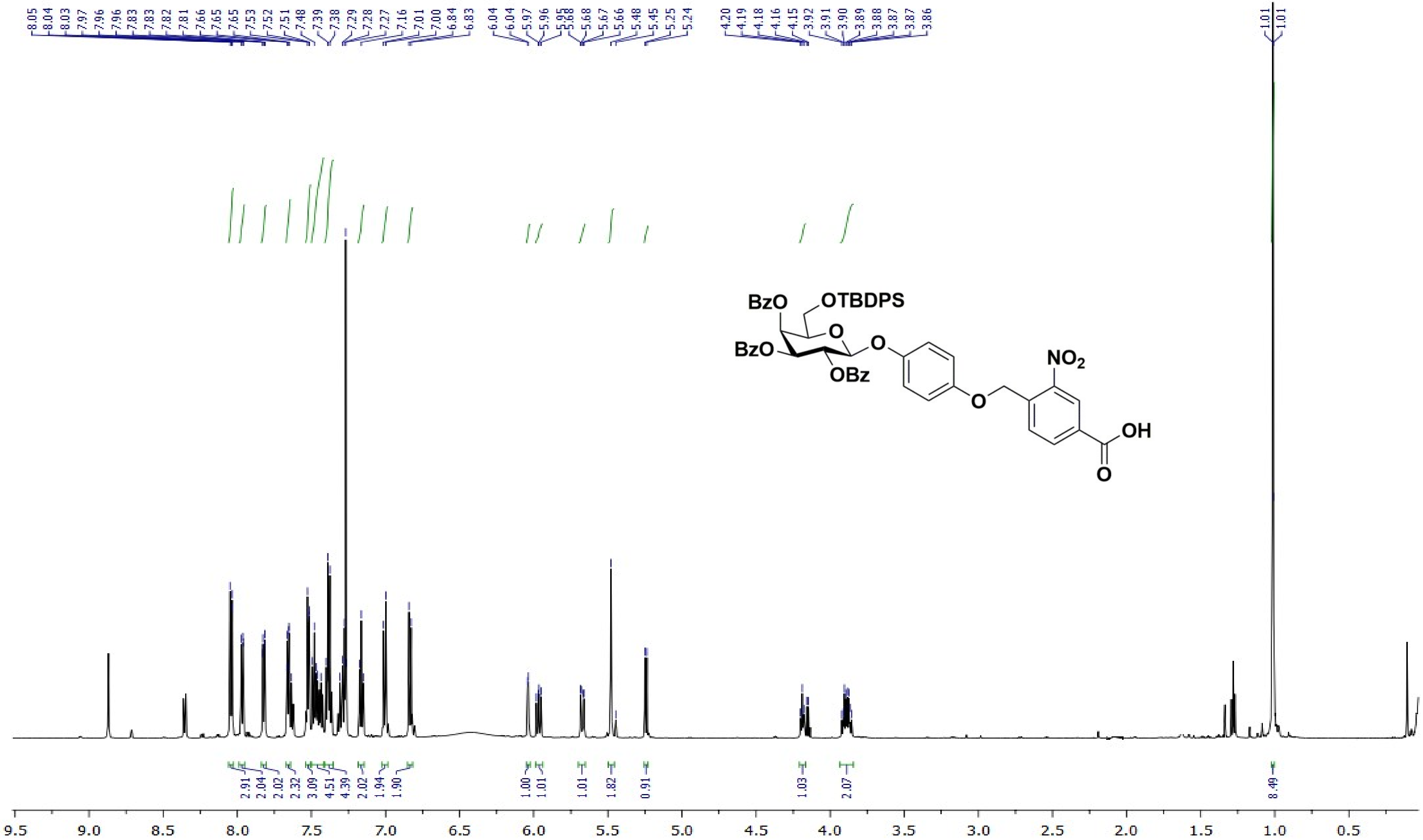

^{13}C NMR of 4-((4-carboxylate-2-nitrobenzyl)oxy)phenyl-2,3,4-tri-*O*-benzoyl-6-*O*-*t*-butyldiphenylsilyl *β*-D-galactopyranoside (6)

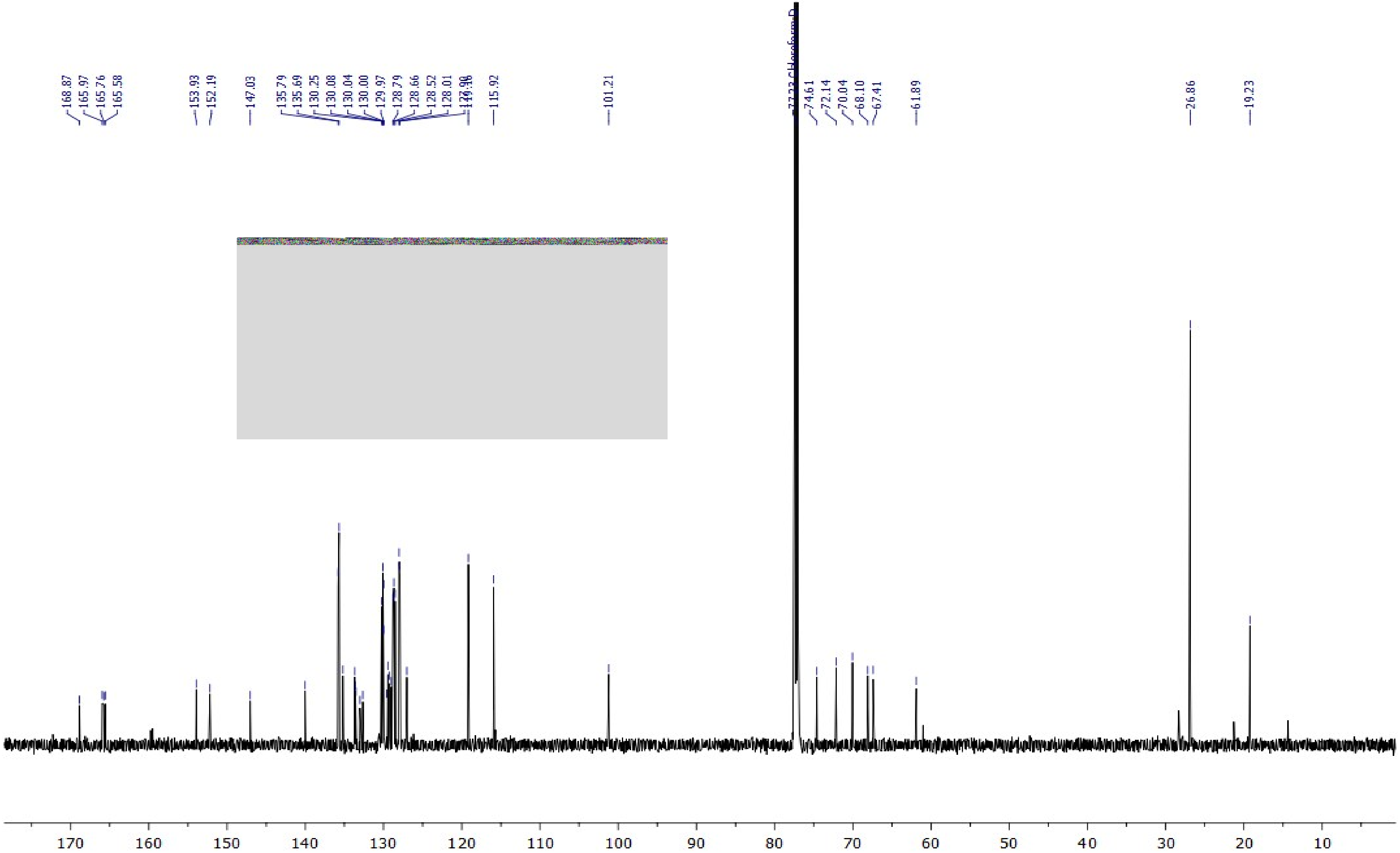

COSY NMR of 4-((4-carboxylate-2-nitrobenzyl)oxy)phenyl-2,3,4-tri-*O*-benzoyl-6-*O*-*t*-butyldiphenylsilyl *β*-D-galactopyranoside (6)

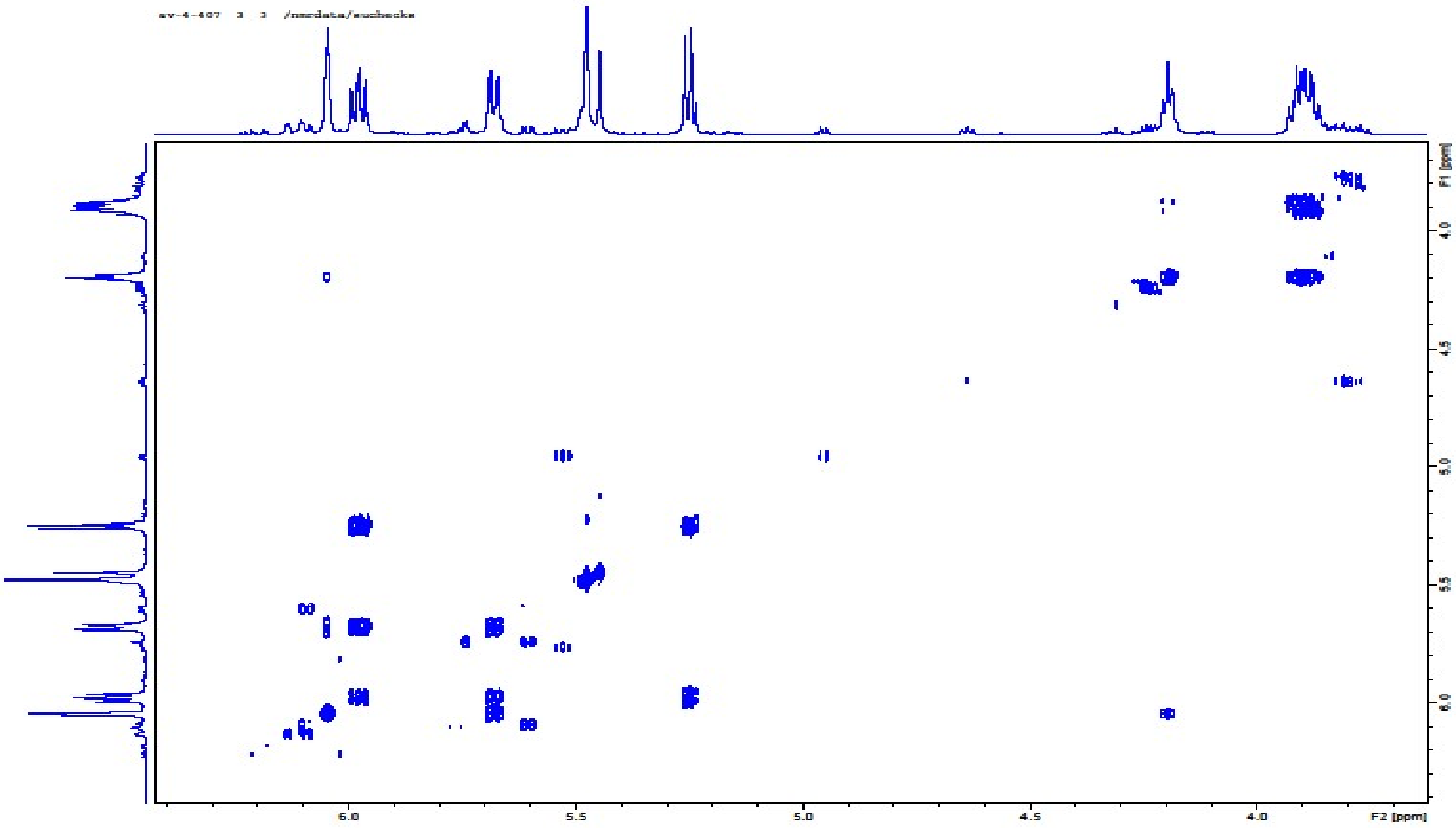

ESI-MS data of 4-hydroxyphenyl-2,3,4-tri-*O*-benzoyl-6-*O*-*t*-butyldiphenylsilyl-β-D-galacopyranoside (7a)

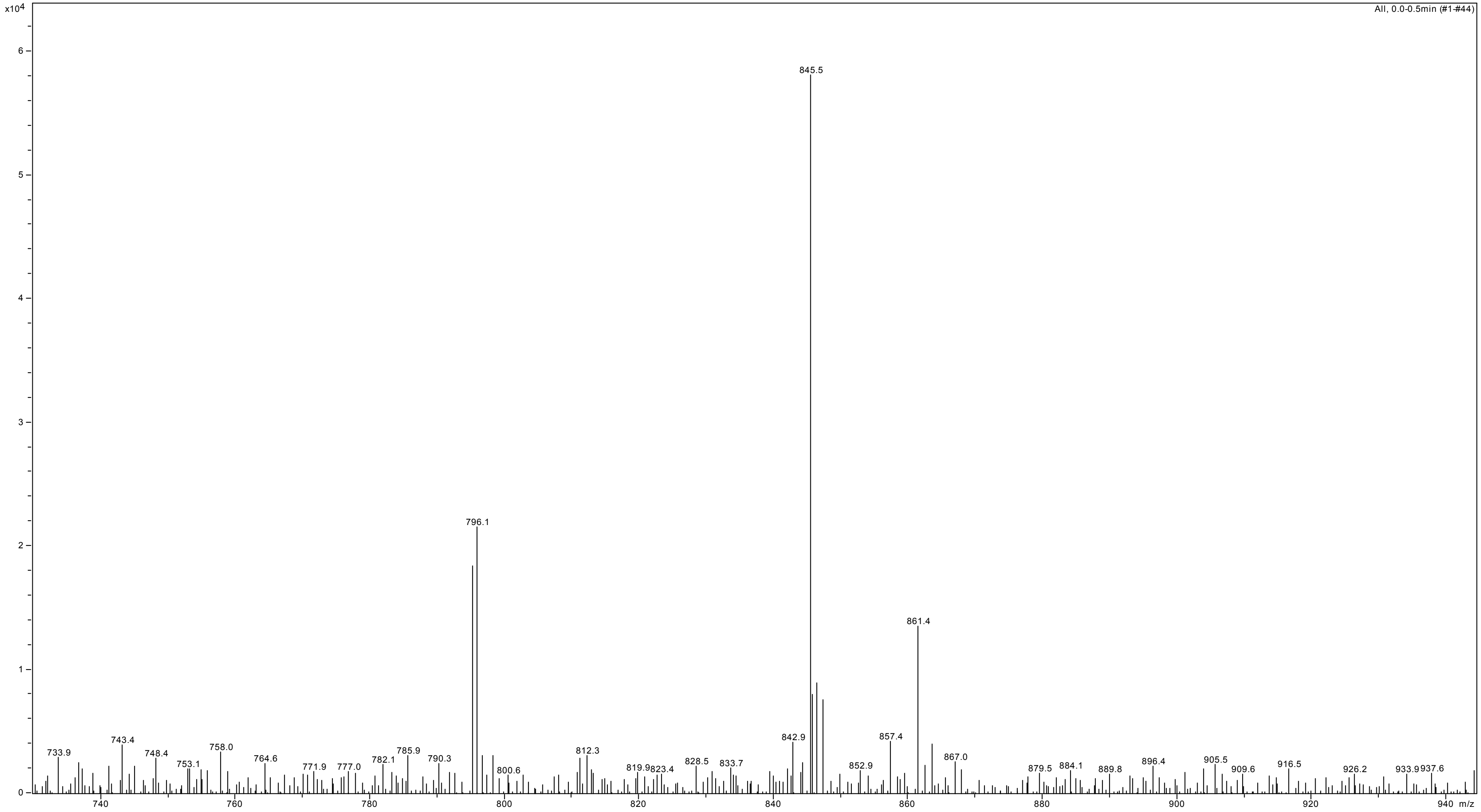

^{1}H NMR of 4-hydroxyphenyl-2,3,4-tri-*O*-benzoyl-6-*O*-*t*-butyldiphenylsilyl-β-D-galactopyranoside (7a)

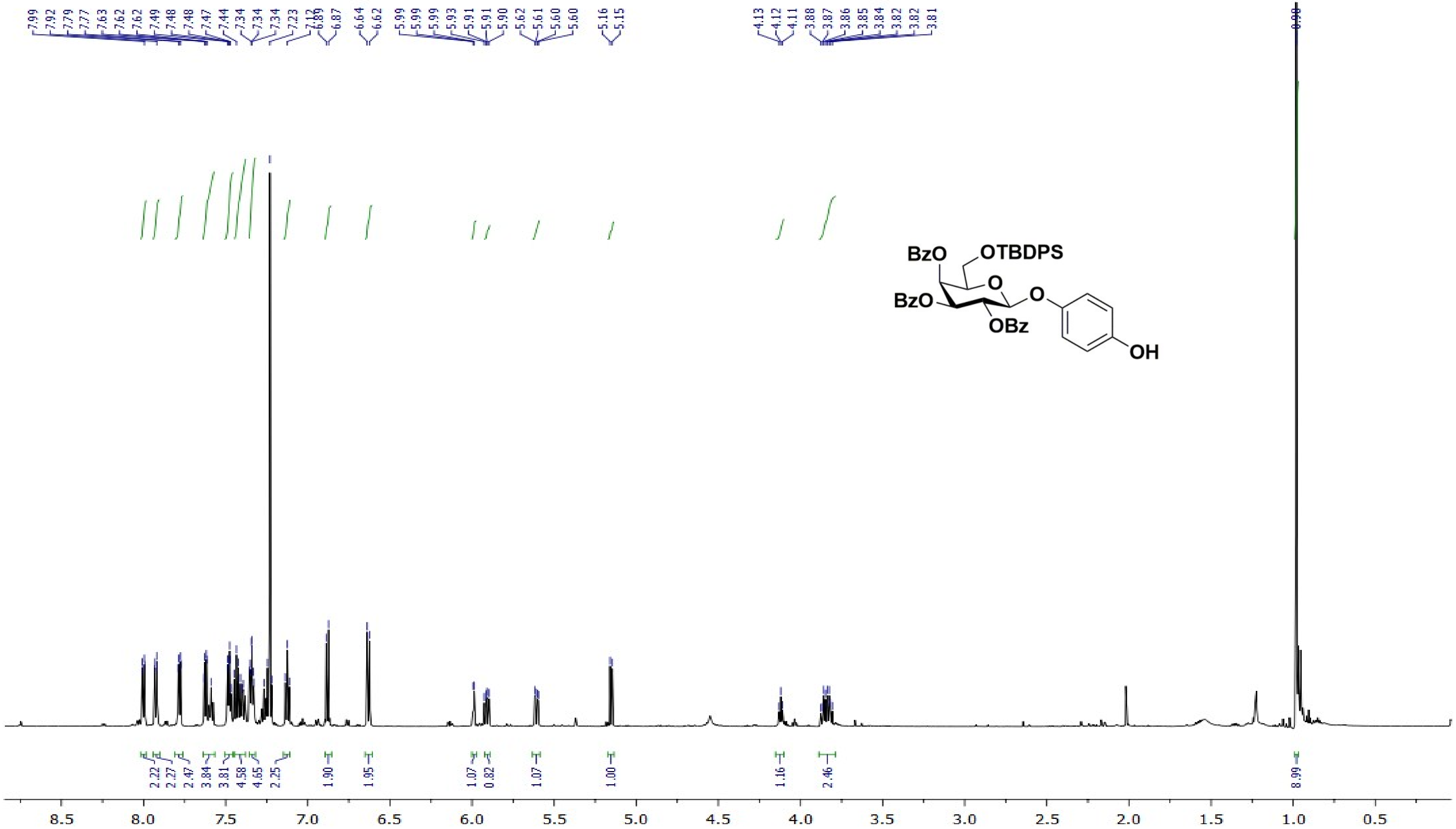

^{13}C NMR of 4-hydroxyphenyl-2,3,4-tri-*O*-benzoyl-6-*O*-*t*-butyldiphenylsilyl-β-D-galactopyranoside (7a)

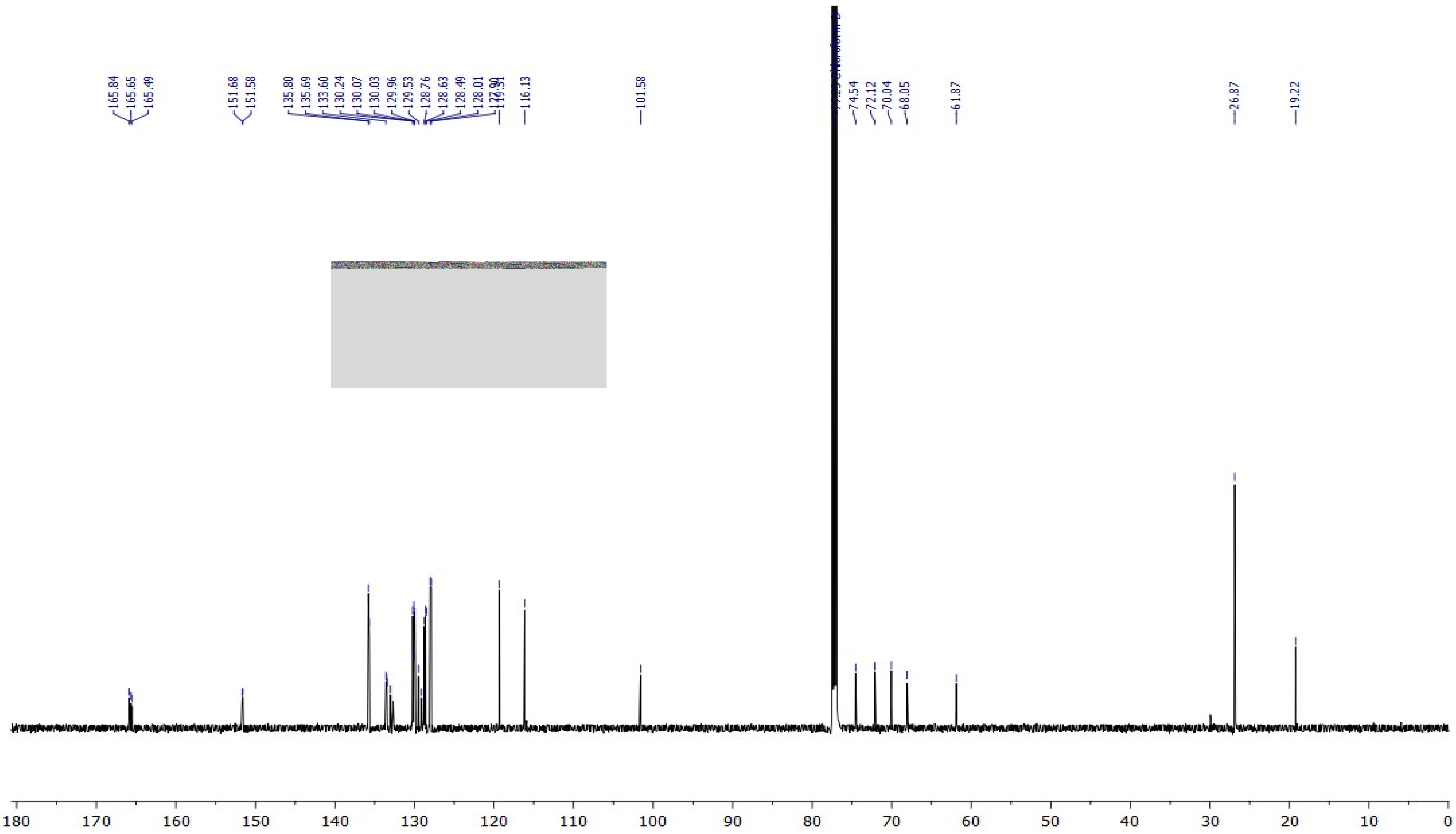

COSY NMR of 4-hydroxyphenyl-2,3,4-tri-*O*-benzoyl-6-*O*-*t*-butyldiphenylsilyl-β-D-galactopyranoside (7a)

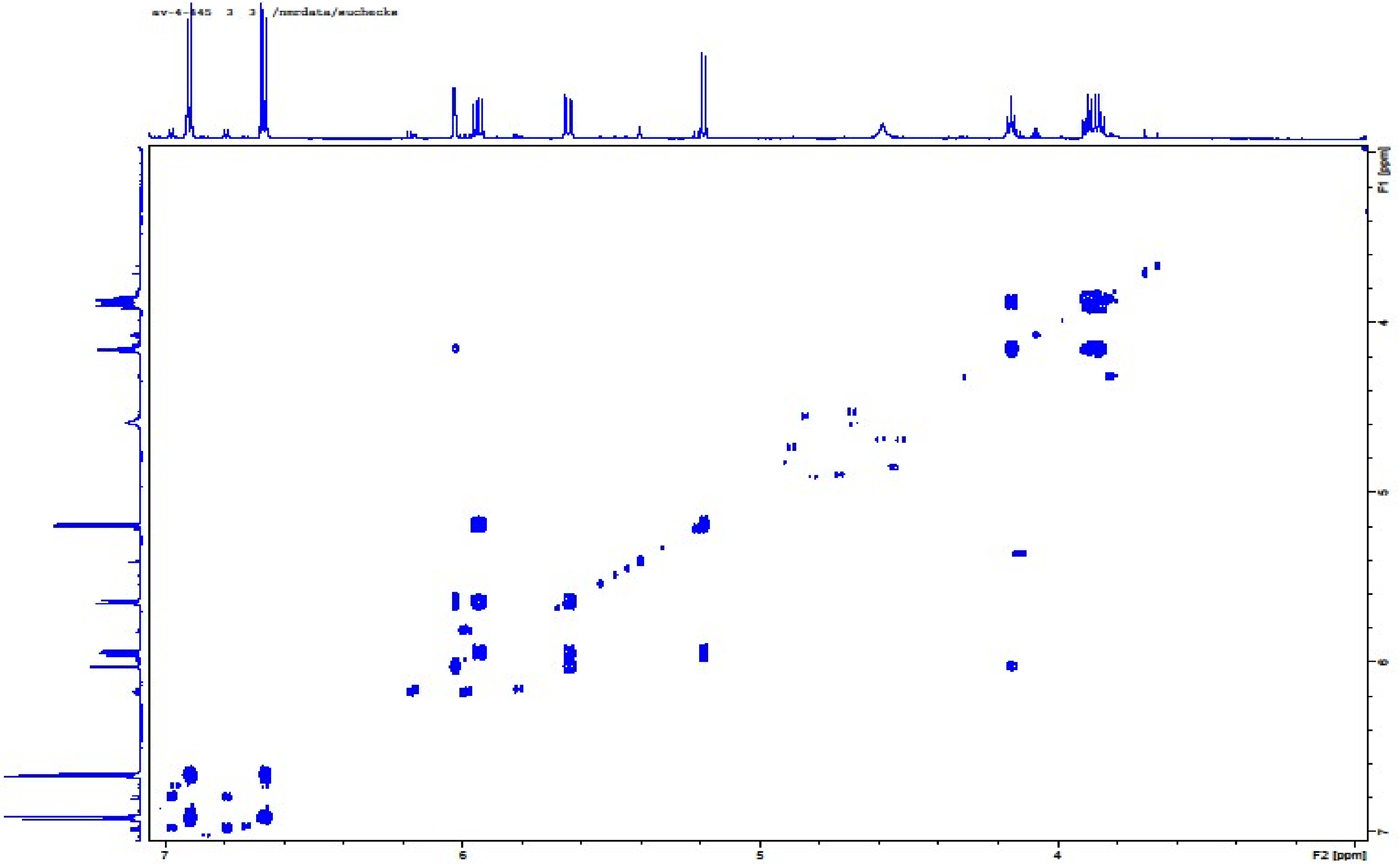

HRMS data of 4-hydroxyphenyl-2,3,4-tri-*O*-benzoyl-6-*O*-*t*-butyldiphenylsilyl-β-D-galactopyranosyl-(1→6)- 2,3,4-tri-*O*-benzyl- β -D-galactopyranoside (9a)

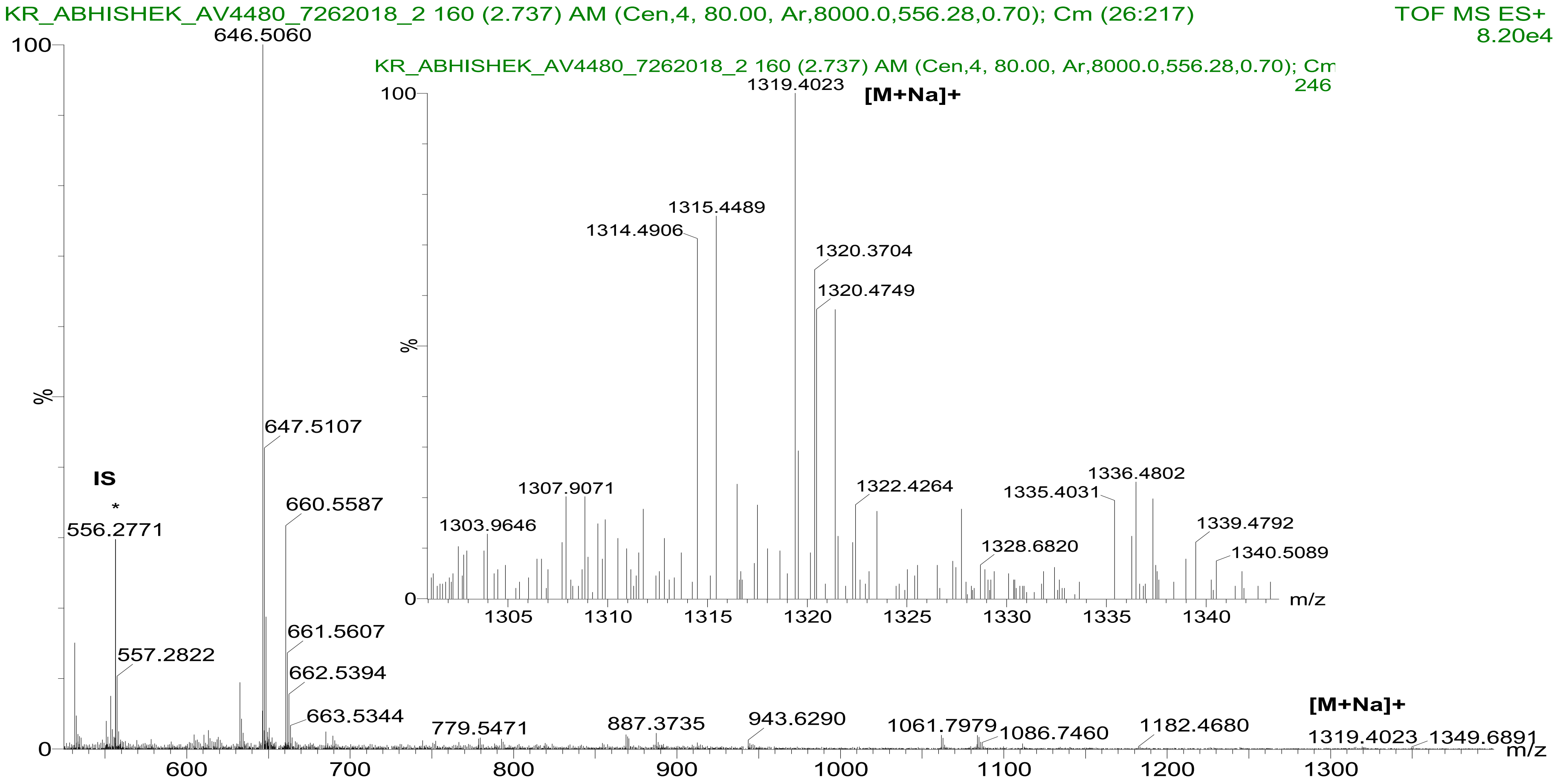

Mass accuracy = ((1319.4072-1319.4023)/1319.4072)* 106 = 3.7 ppm

^{1}H NMR of 4-hydroxyphenyl-2,3,4-tri-*O*-benzoyl-6-*O*-*t*-butyldiphenylsilyl-β-D-galactopyranosyl-(1→6)- 2,3,4-tri-*O*-benzyl- β -D-galactopyranoside (9a)

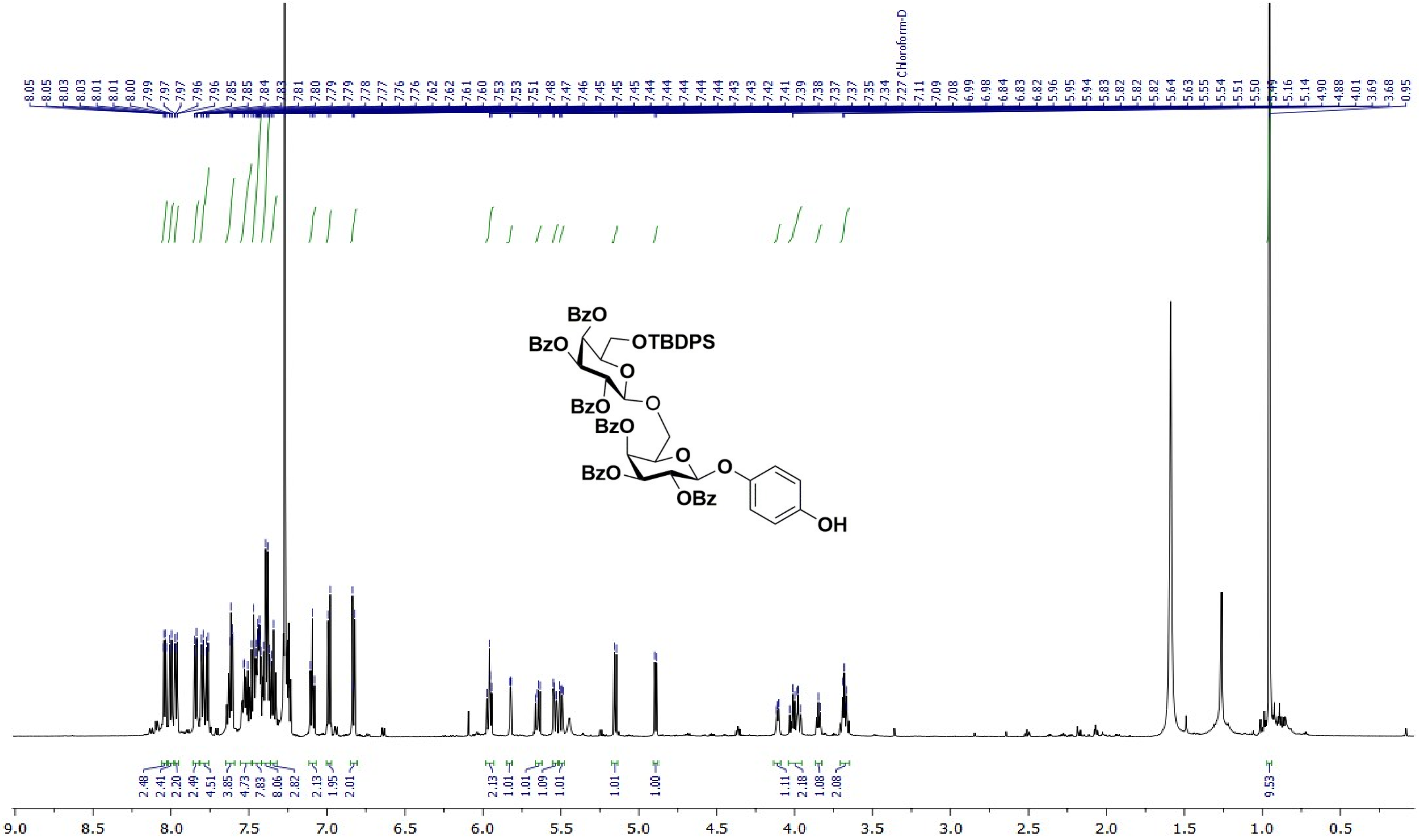

^{1}H NMR of 4-hydroxyphenyl-2,3,4-tri-*O*-benzoyl-6-*O*-*t*-butyldiphenylsilyl-β-D-galactopyranosyl-(1→6)- 2,3,4-tri-*O*-benzyl- β -D-galactopyranoside (9a)

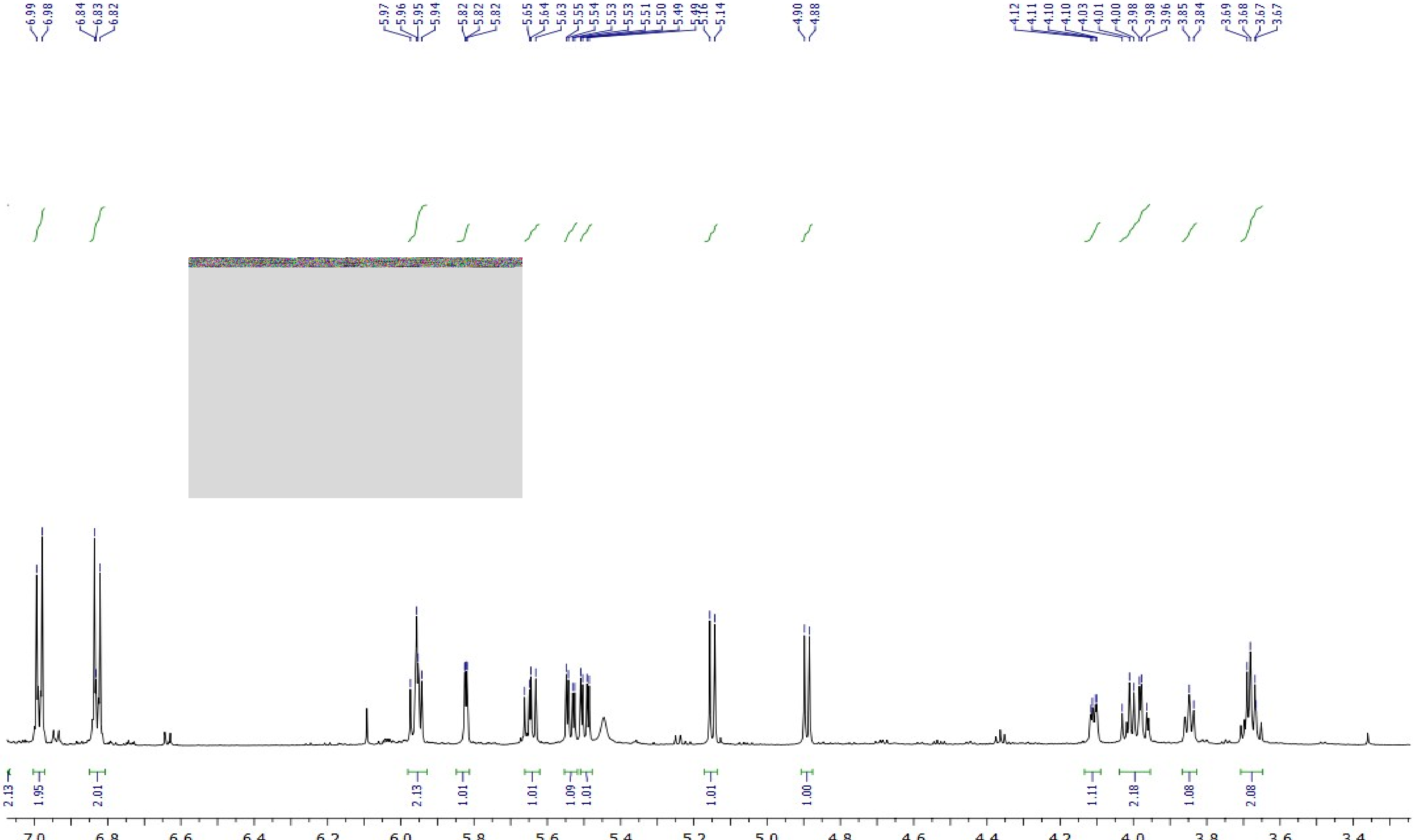

^{13}C NMR of 4-hydroxyphenyl-2,3,4-tri-*O*-benzoyl-6-*O*-*t*-butyldiphenylsilyl-β-D-galactopyranosyl-(1→6)- 2,3,4-tri-*O*-benzyl- β -D-galactopyranoside (9a)

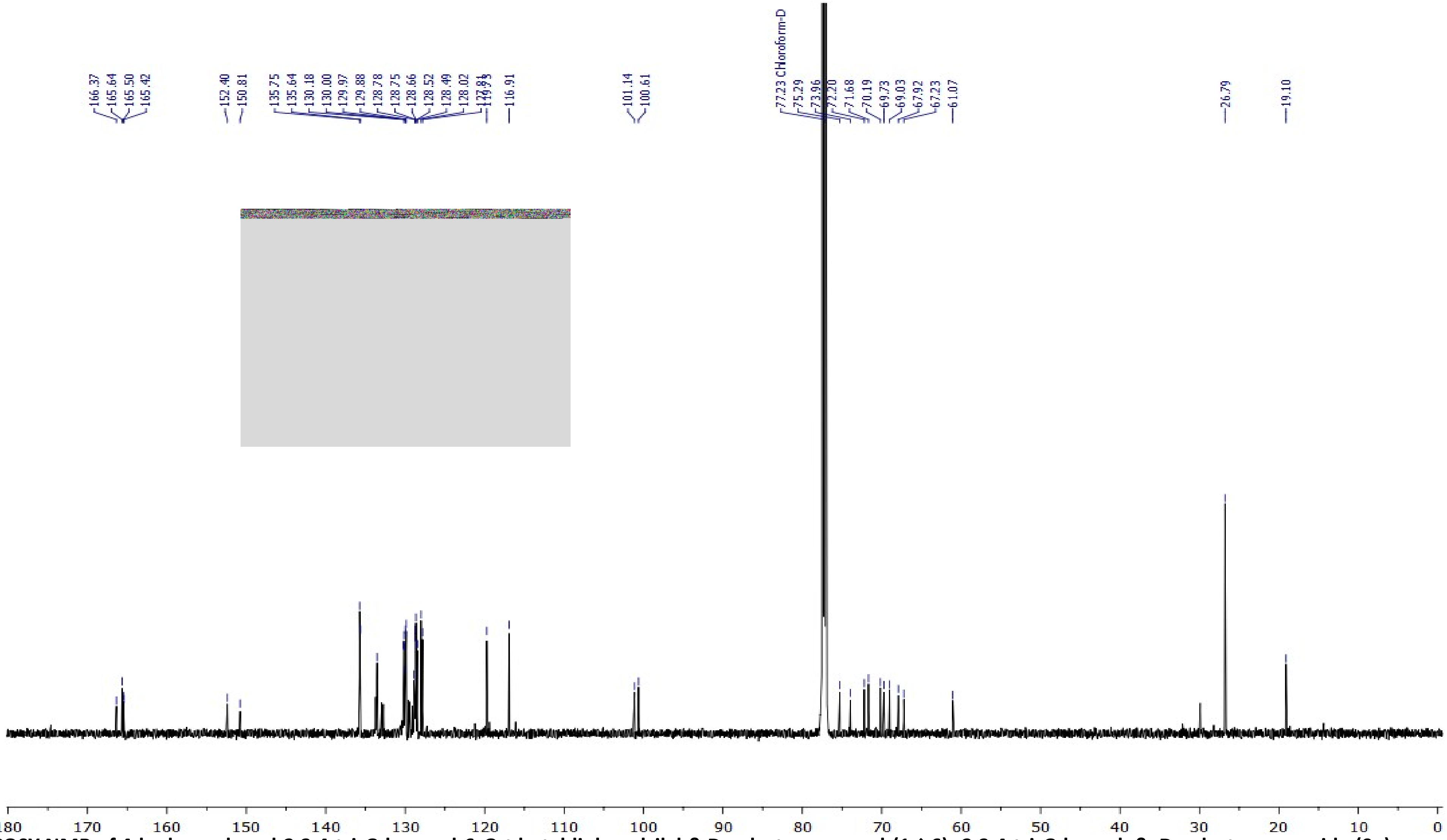

COSY NMR of 4-hydroxyphenyl-2,3,4-tri-*O*-benzoyl-6-*O*-*t*-butyldiphenylsilyl-β-D-galactopyranosyl-(1→6)- 2,3,4-tri-*O*-benzyl- β -D-galactopyranoside (9a)

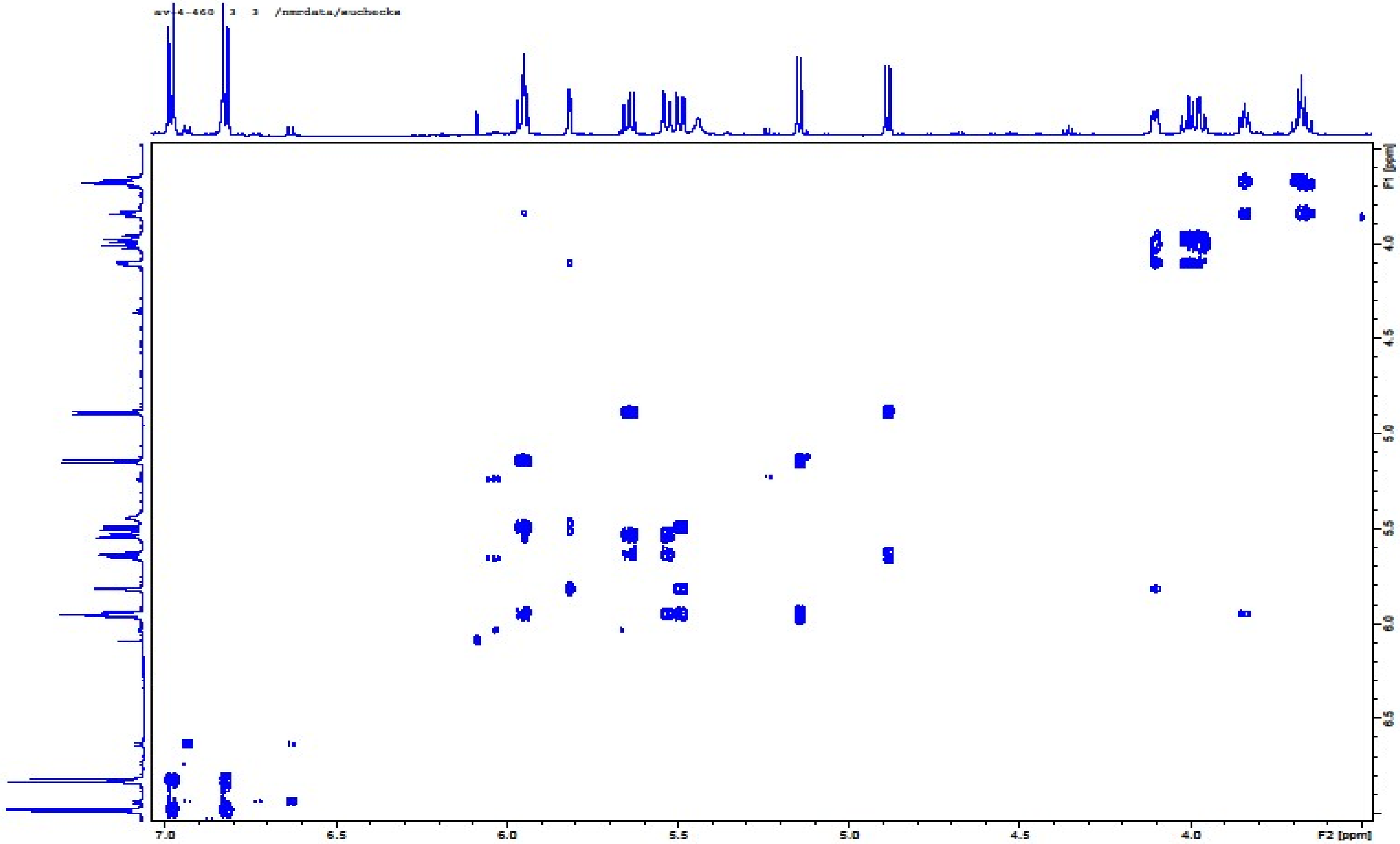
7.0
6.5
6.0
5.5
5.0
4.5
4.0
F2 [ppm]
F1 [ppm]
4.0
4.5
5.0
5.5
6.0
6.5

HRMS data of 4-hydroxyphenyl-2,3,4-tri-*O*-benzoyl-6-*O*-*t*-butyldiphenylsilyl-β-D-galactopyranosyl-(1→6)- 2,3,4-tri-*O*-benzyl- β -D-galactopyranosyl-(1→6)- 2,3,4-tri-*O*-benzyl- β -D-galactopyranoside (11a)

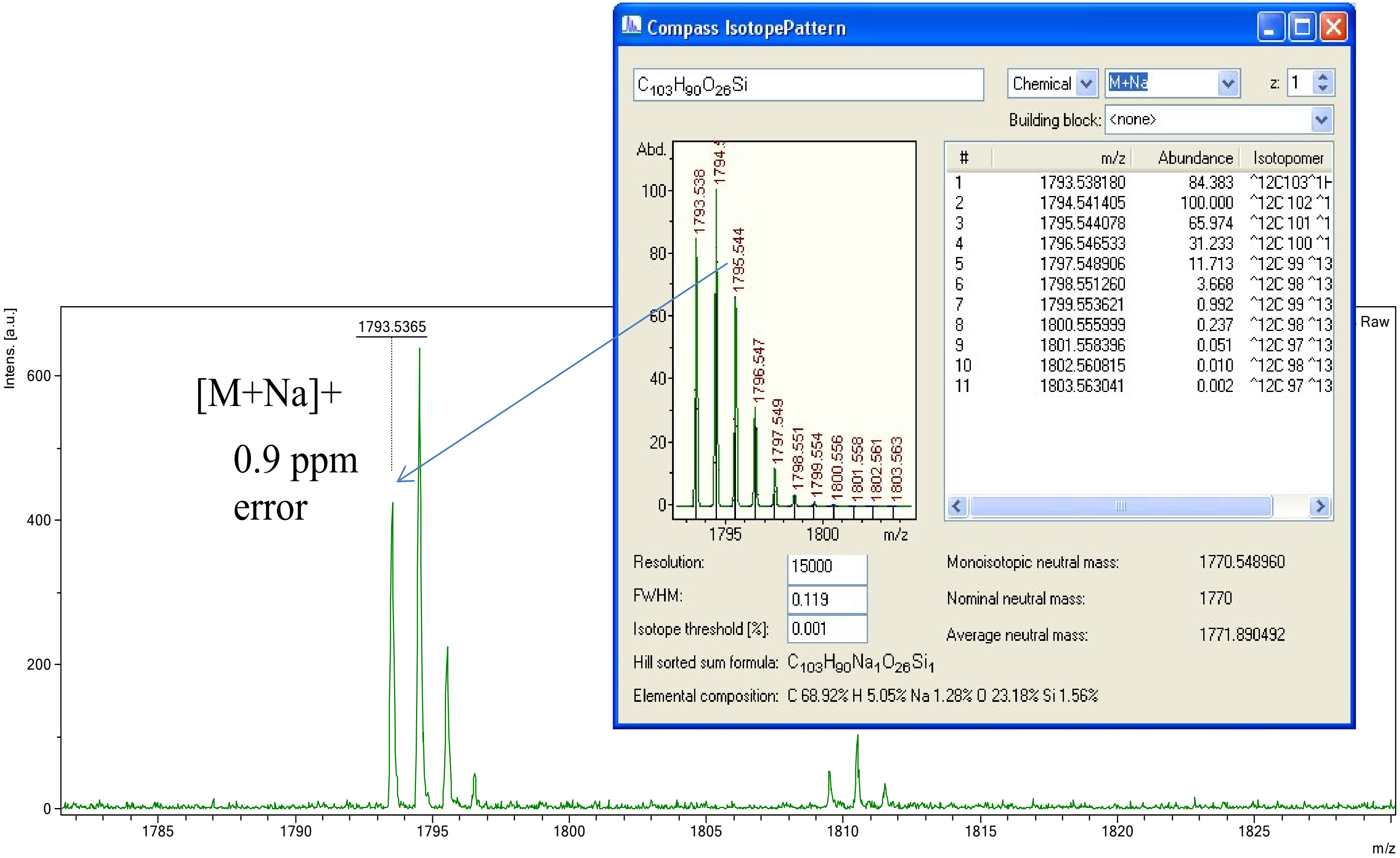

#	m/z	Abundance	Isotopomer
1	1793.538180	84.383	^12C103^1H
2	1794.541405	100.000	^12C 102 ^1
3	1795.544078	65.974	^12C 101 ^1
4	1796.546533	31.233	^12C 100 ^1
5	1797.548906	11.713	^12C 99 ^13
6	1798.551260	3.668	^12C 98 ^13
7	1799.553621	0.992	^12C 99 ^13
8	1800.555999	0.237	^12C 98 ^13
9	1801.558396	0.051	^12C 97 ^13
10	1802.560815	0.010	^12C 98 ^13
11	1803.563041	0.002	^12C 97 ^13

^{1}H NMR of 4-hydroxyphenyl-2,3,4-tri-*O*-benzoyl-6-*O*-*t*-butyldiphenylsilyl-β-D-galactopyranosyl-(1→6)- 2,3,4-tri-*O*-benzyl- β -D-galactopyranosyl-(1→6)- 2,3,4-tri-*O*-benzyl- β -D-galactopyranoside (11a)

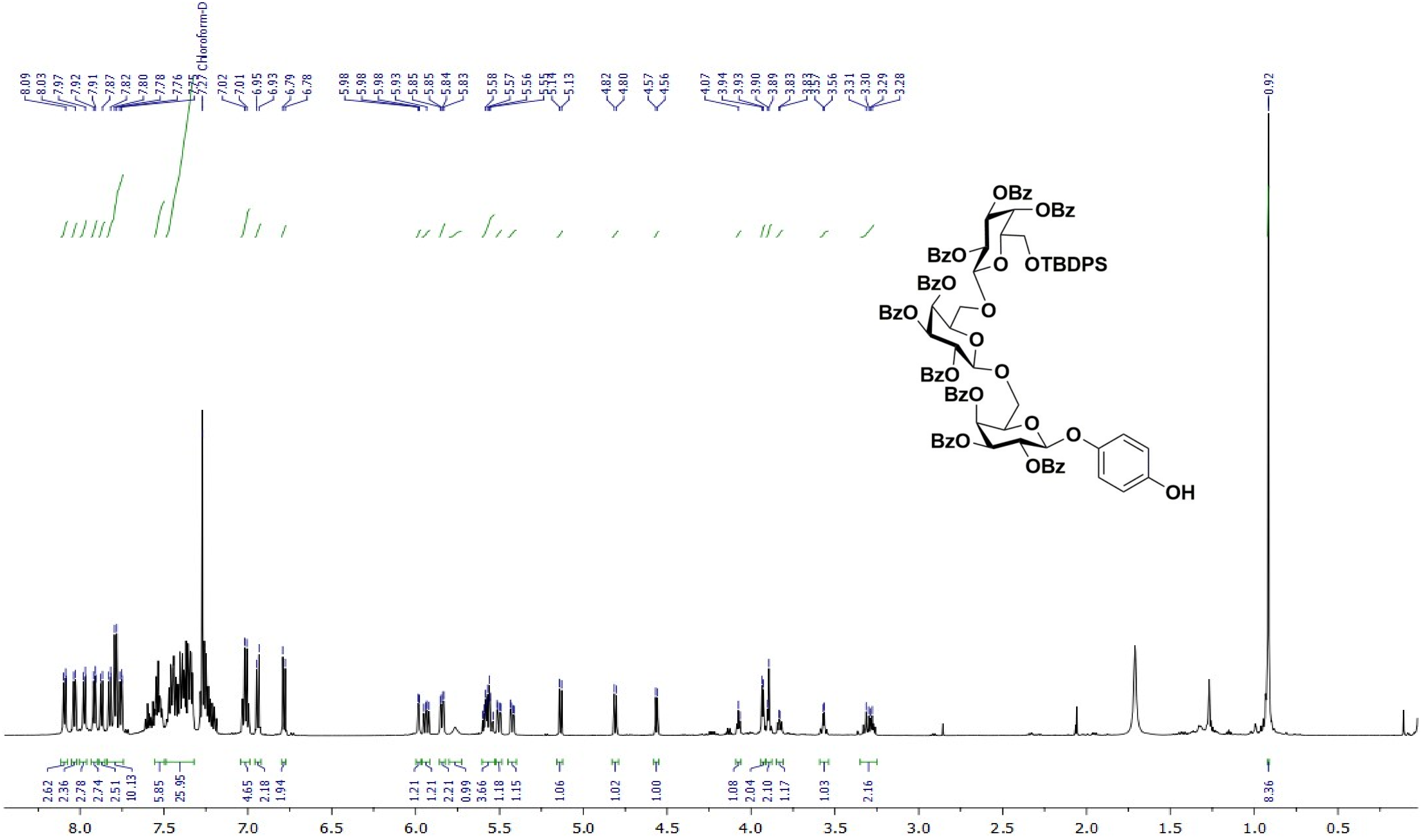

^{1}H NMR of 4-hydroxyphenyl-2,3,4-tri-*O*-benzoyl-6-*O*-*t*-butyldiphenylsilyl-β-D-galactopyranosyl-(1→6)- 2,3,4-tri-*O*-benzyl- β -D-galactopyranosyl-(1→6)-2,3,4-tri-*O*-benzyl- β -D-galactopyranoside (11a)

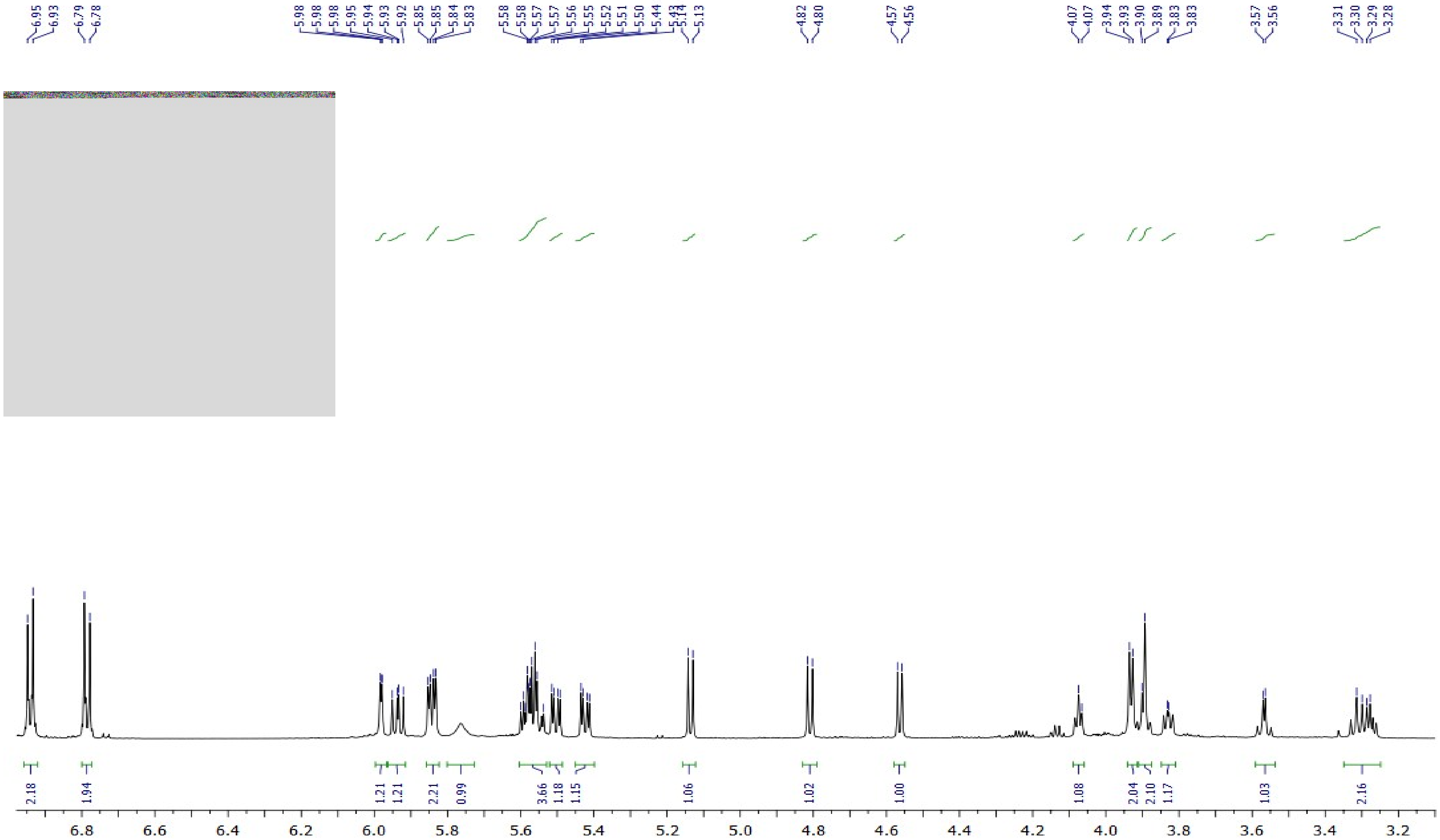

^{13}C NMR of 4-hydroxyphenyl-2,3,4-tri-*O*-benzoyl-6-*O*-*t*-butyldiphenylsilyl-β-D-galactopyranosyl-(1→6)- 2,3,4-tri-*O*-benzyl- β -D-galactopyranosyl-(1→6)-2,3,4-tri-*O*-benzyl- β -D-galactopyranoside (11a)

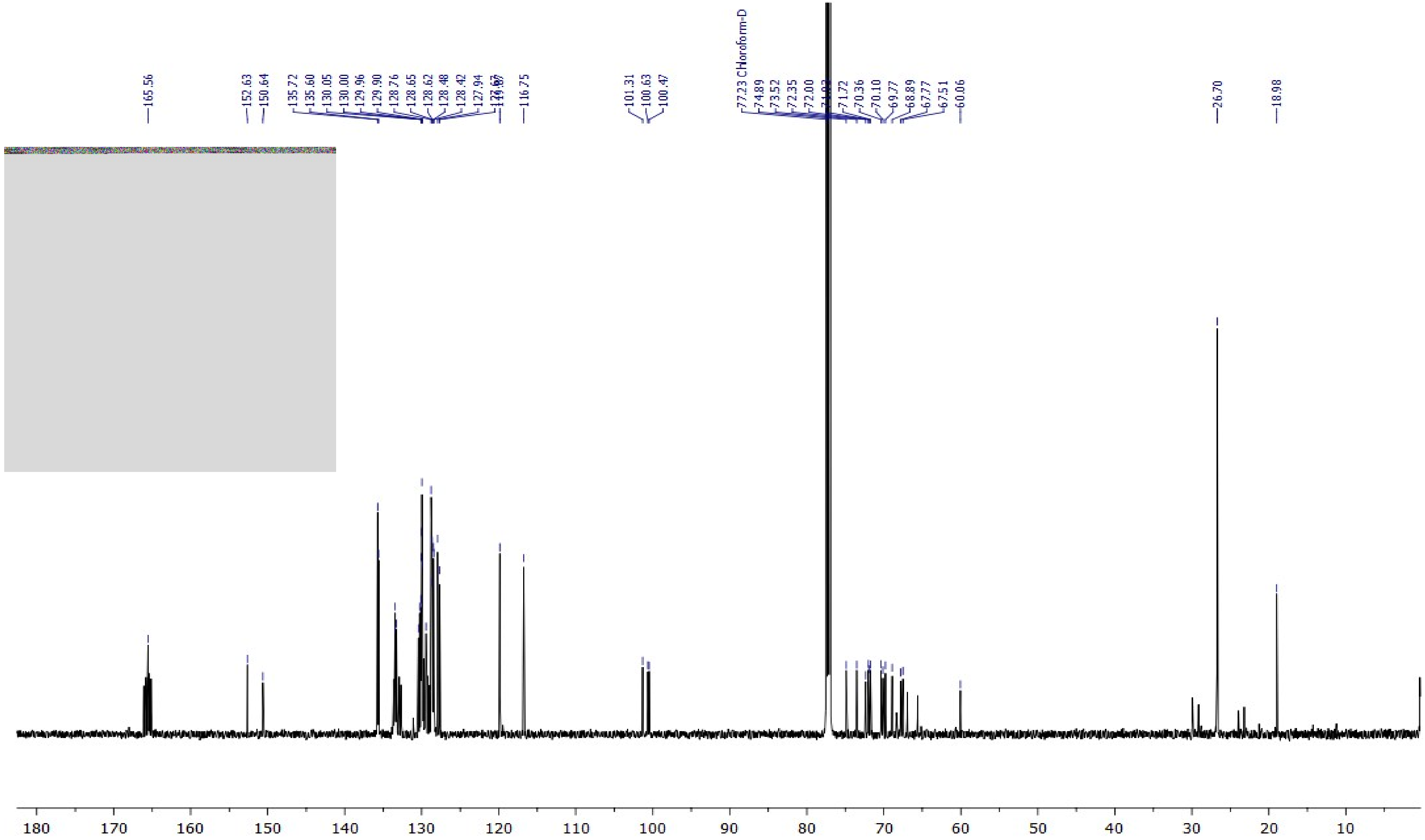

COSY NMR of 4-hydroxyphenyl-2,3,4-tri-*O*-benzoyl-6-*O*-*t*-butyldiphenylsilyl-β-D-galactopyranosyl-(1→6)- 2,3,4-tri-*O*-benzyl- β -D-galactopyranosyl-(1→6)- 2,3,4-tri-*O*-benzyl- β -D-galactopyranoside (11a)

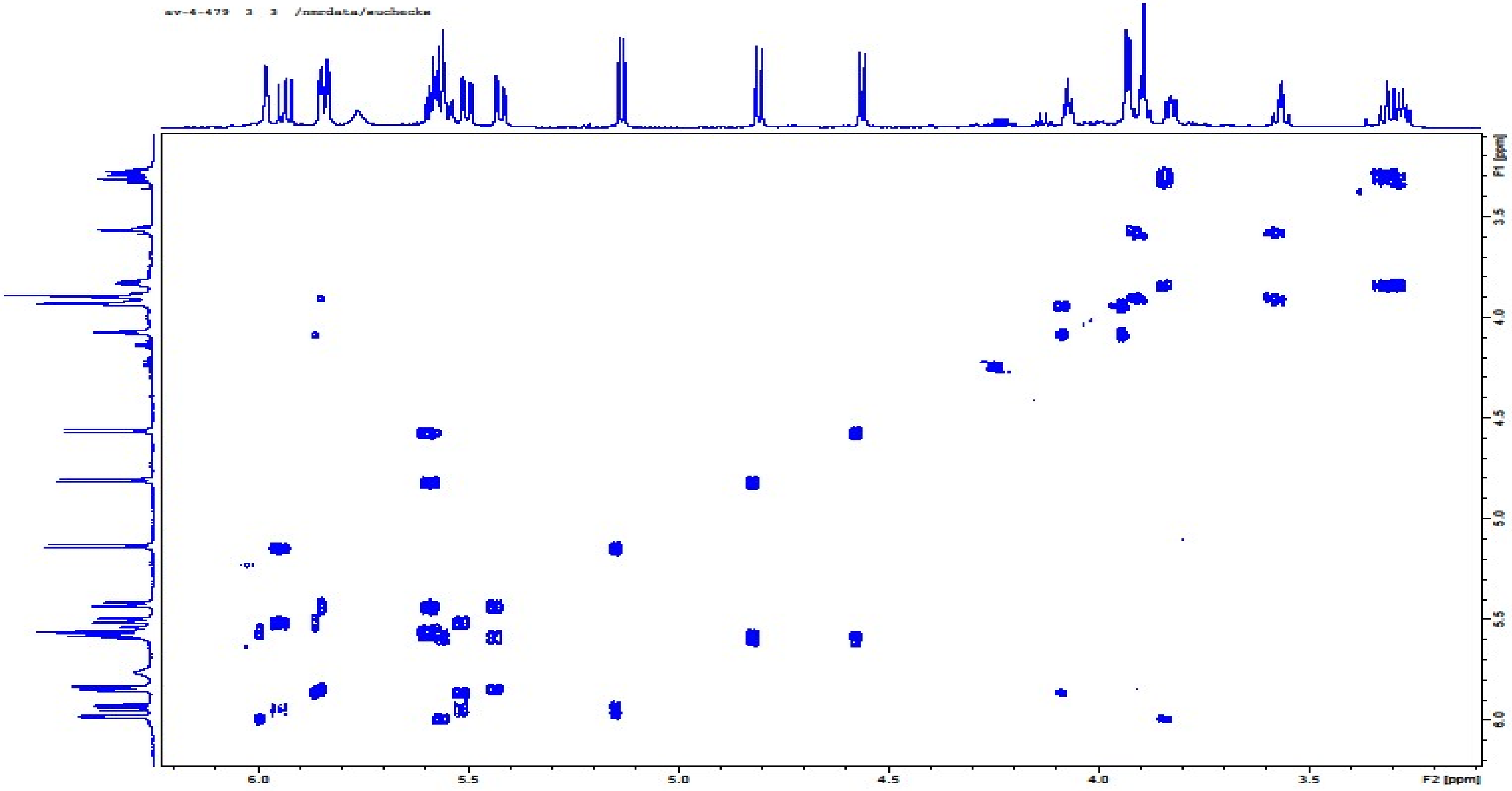

HRMS data of 4-((4-carboxylate-2-nitrobenzyl)oxy)phenyl-2,3,4-tri-*O*-benzoyl-6-*O*-*t*-butyldiphenylsilyl *β*-D-glucopyranoside (12)

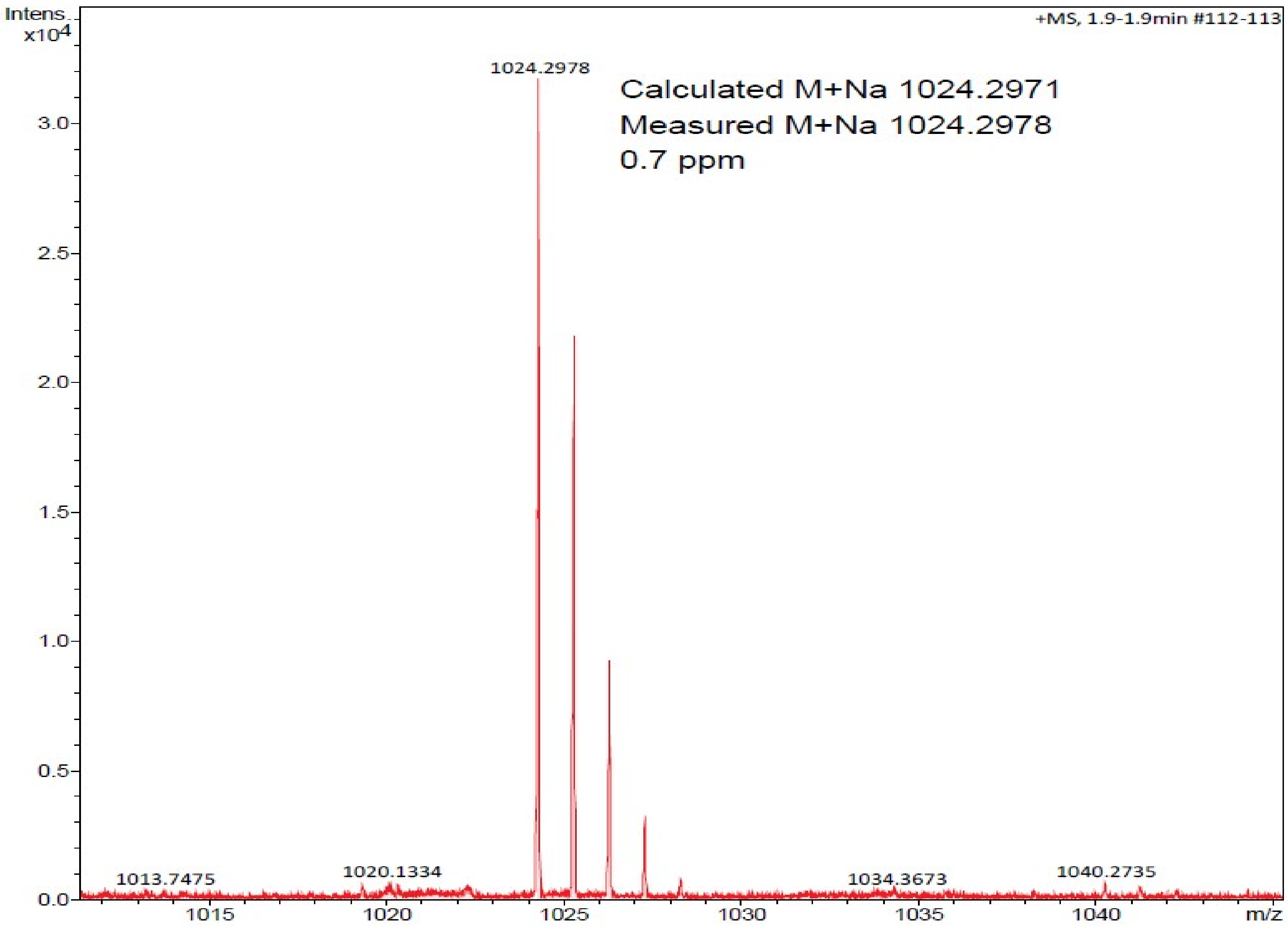

^{1}H NMR of 4-((4-carboxylate-2-nitrobenzyl)oxy)phenyl-2,3,4-tri-*O*-benzoyl-6-*O*-*t*-butyldiphenylsilyl *β*-D-glucopyranoside (12)

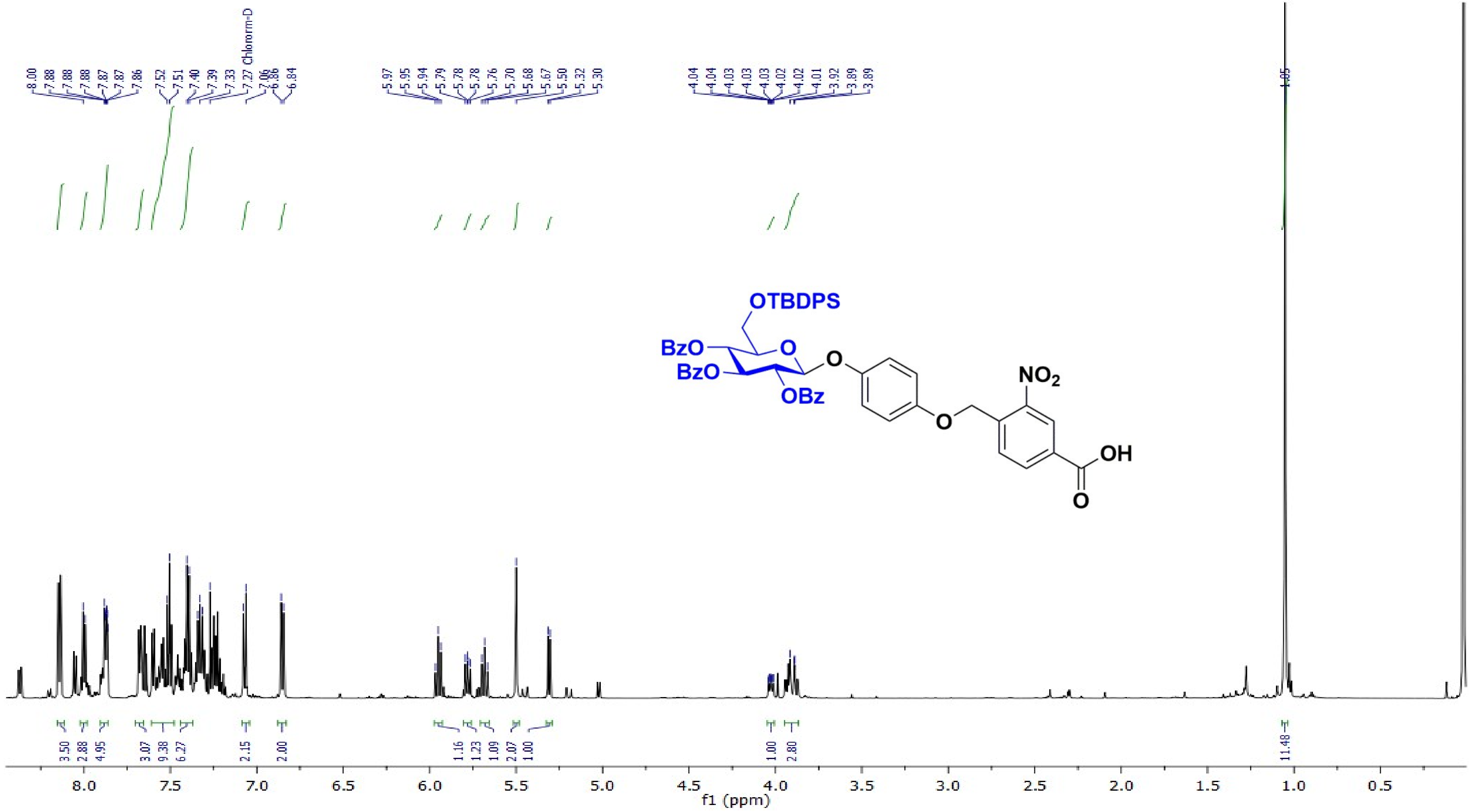

^{13}C NMR of 4-((4-carboxylate-2-nitrobenzyl)oxy)phenyl-2,3,4-tri-*O*-benzoyl-6-*O*-*t*-butyldiphenylsilyl *β*-D-glucopyranoside (12)

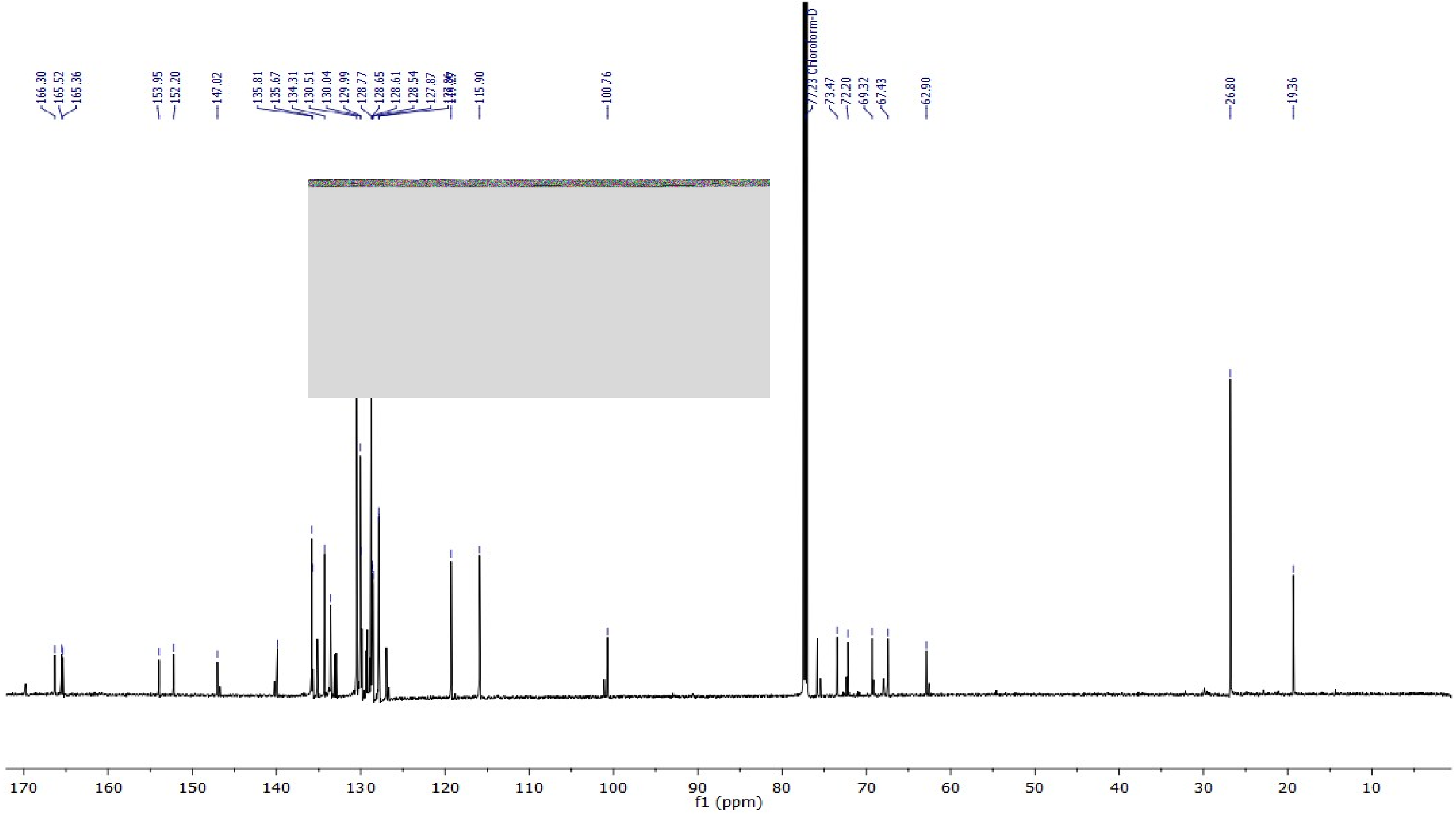

COSY NMR of 4-((4-carboxylate-2-nitrobenzyl)oxy)phenyl-2,3,4-tri-*O*-benzoyl-6-*O*-*t*-butyldiphenylsilyl *β*-D-glucopyranoside (12)

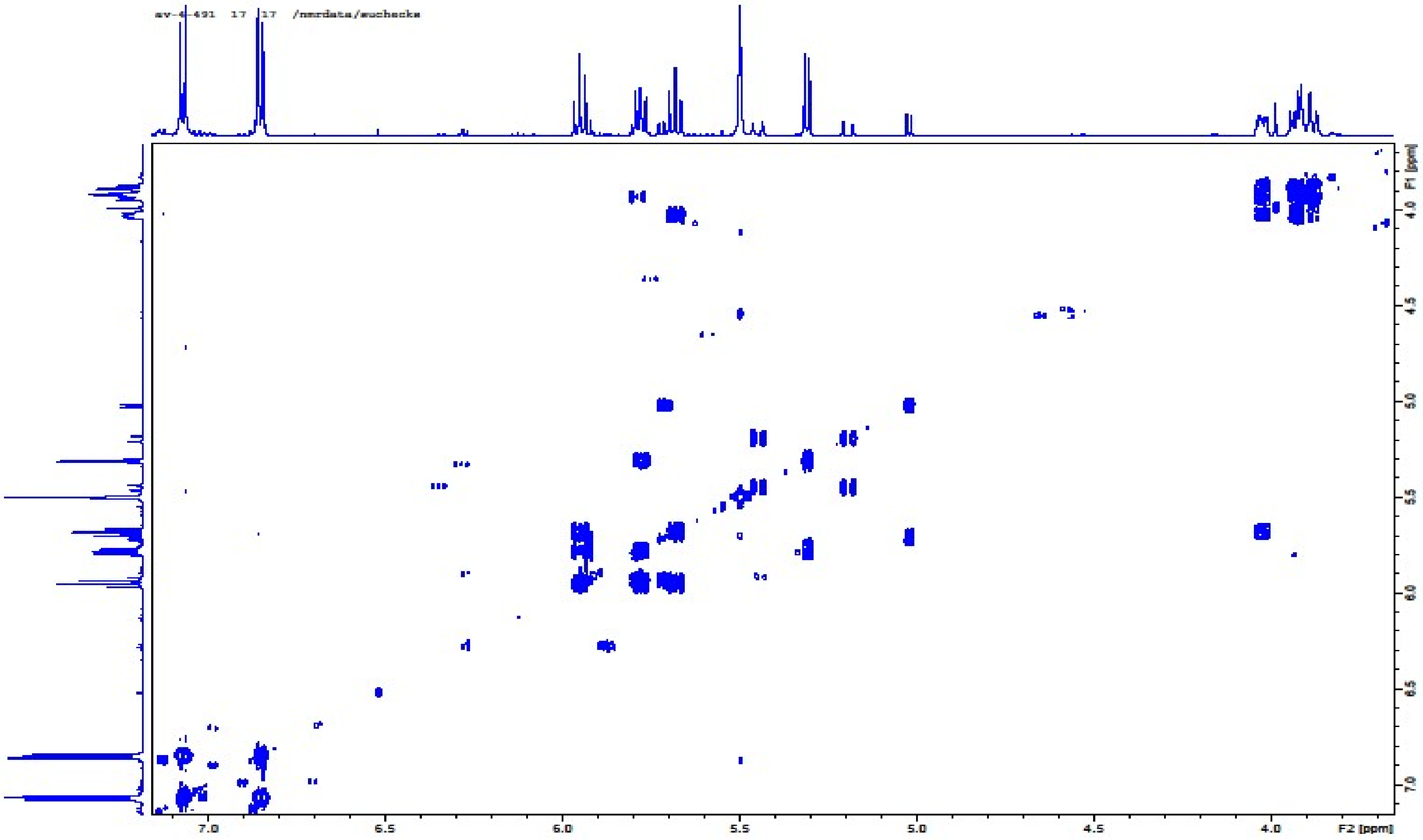

HRMS data of 4-hydroxyphenyl-2-*O*-levulinate-3,4-di-*O*-benzyl-α-L-rhamnopyranosyl-(1→6)-2,3,4-tri-*O*-benzyl-β-D-glucopyranoside (15a)

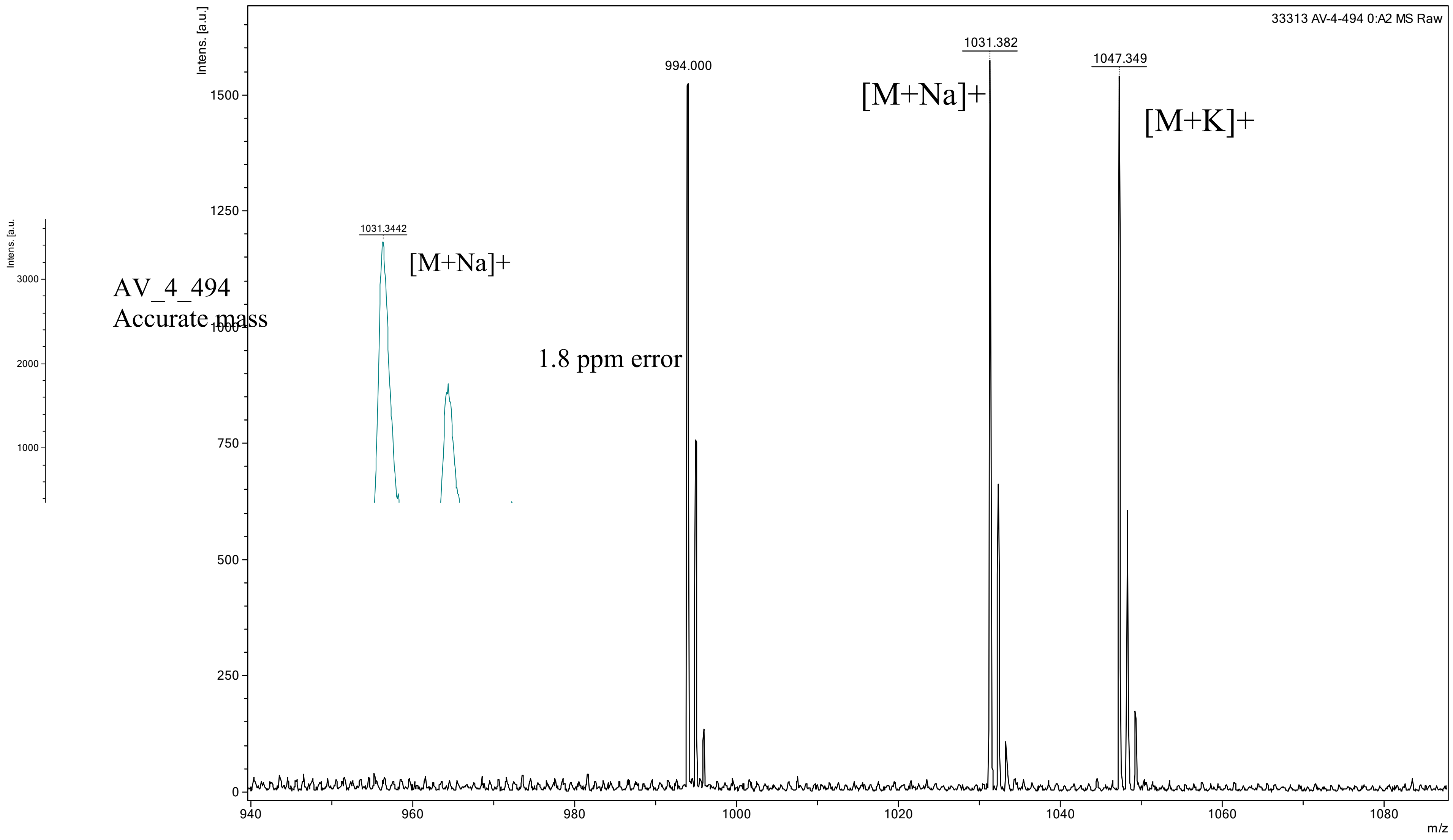

^{1}H NMR of 4-hydroxyphenyl-2-*O*-levulinate-3,4-di-*O*-benzyl-α-L-rhamnopyranosyl-(1→6)-2,3,4-tri-*O*-benzyl-β-D-glucopyranoside (15a)

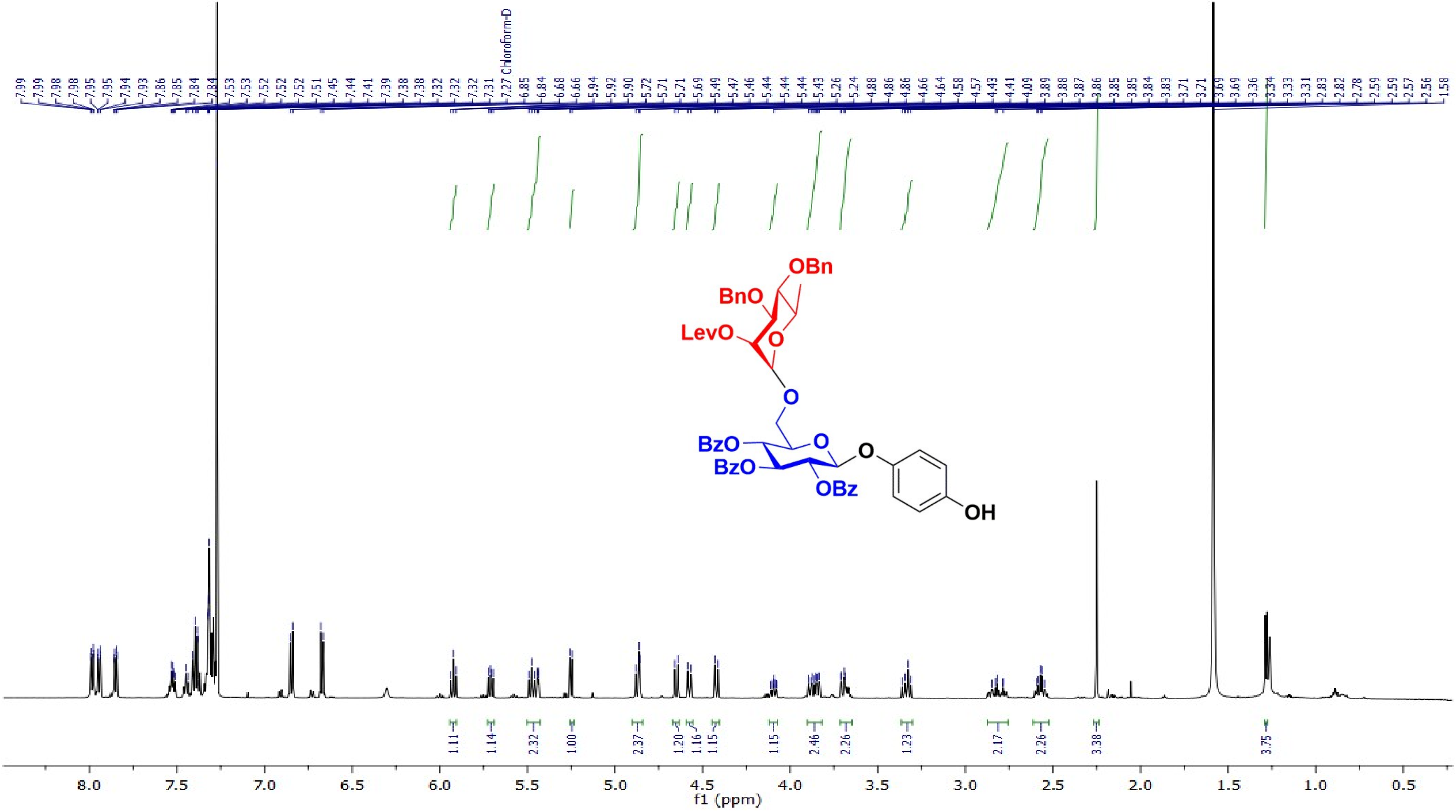

^{13}C NMR of 4-hydroxyphenyl-2-*O*-levulinate-3,4-di-*O*-benzyl-α-L-rhamnopyranosyl-(1→6)-2,3,4-tri-*O*-benzyl-β-D-glucopyranoside (15a)

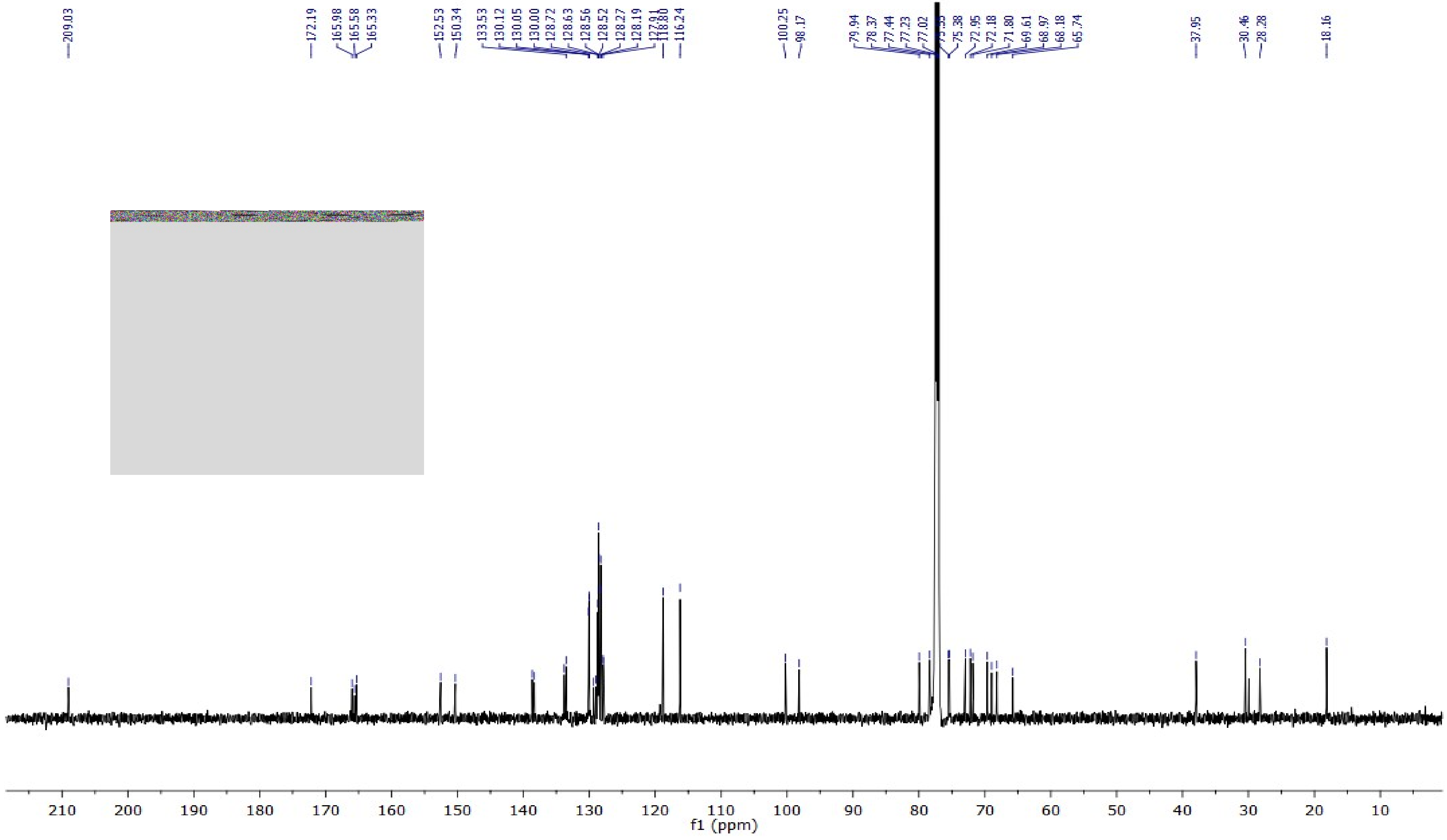

COSY NMR of 4-hydroxyphenyl-2-*O*-levulinate-3,4-di-*O*-benzyl-α-L-rhamnopyranosyl-(1→6)-2,3,4-tri-*O*-benzyl-β-D-glucopyranoside (15a)

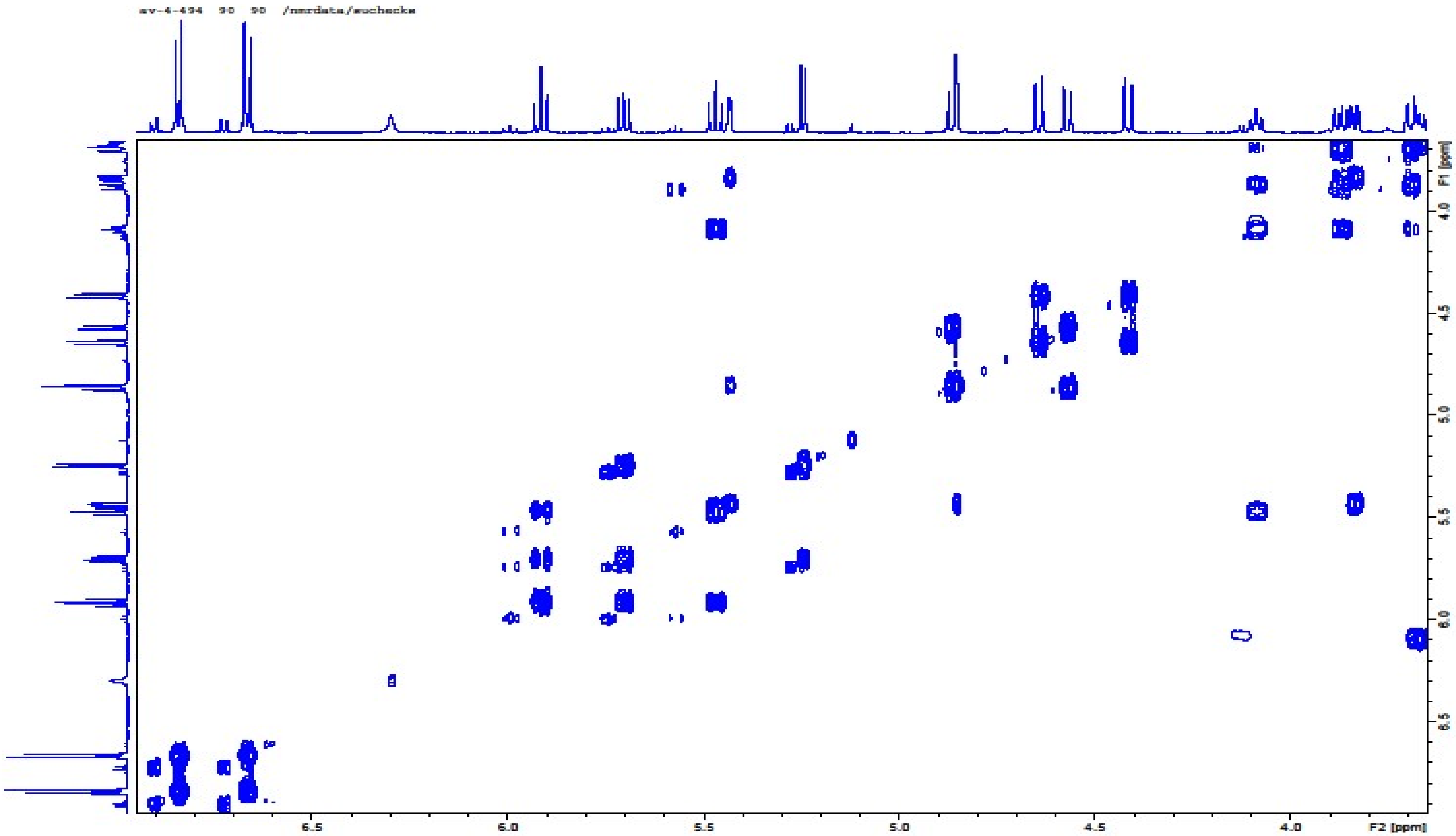

ESI-MS data of 4-hydroxyphenyl-2,3,4-tri-*O*-benzoyl-6-*O*-*t*-butyldiphenylsilyl-β-D-glucopyranosyl-(1→2)-3,4-di-*O*-benzyl-α-L-rhamnopyranosyl-(1→6)-2,3,4-tri-*O*-benzyl-β-D-glucopyranoside (17a)

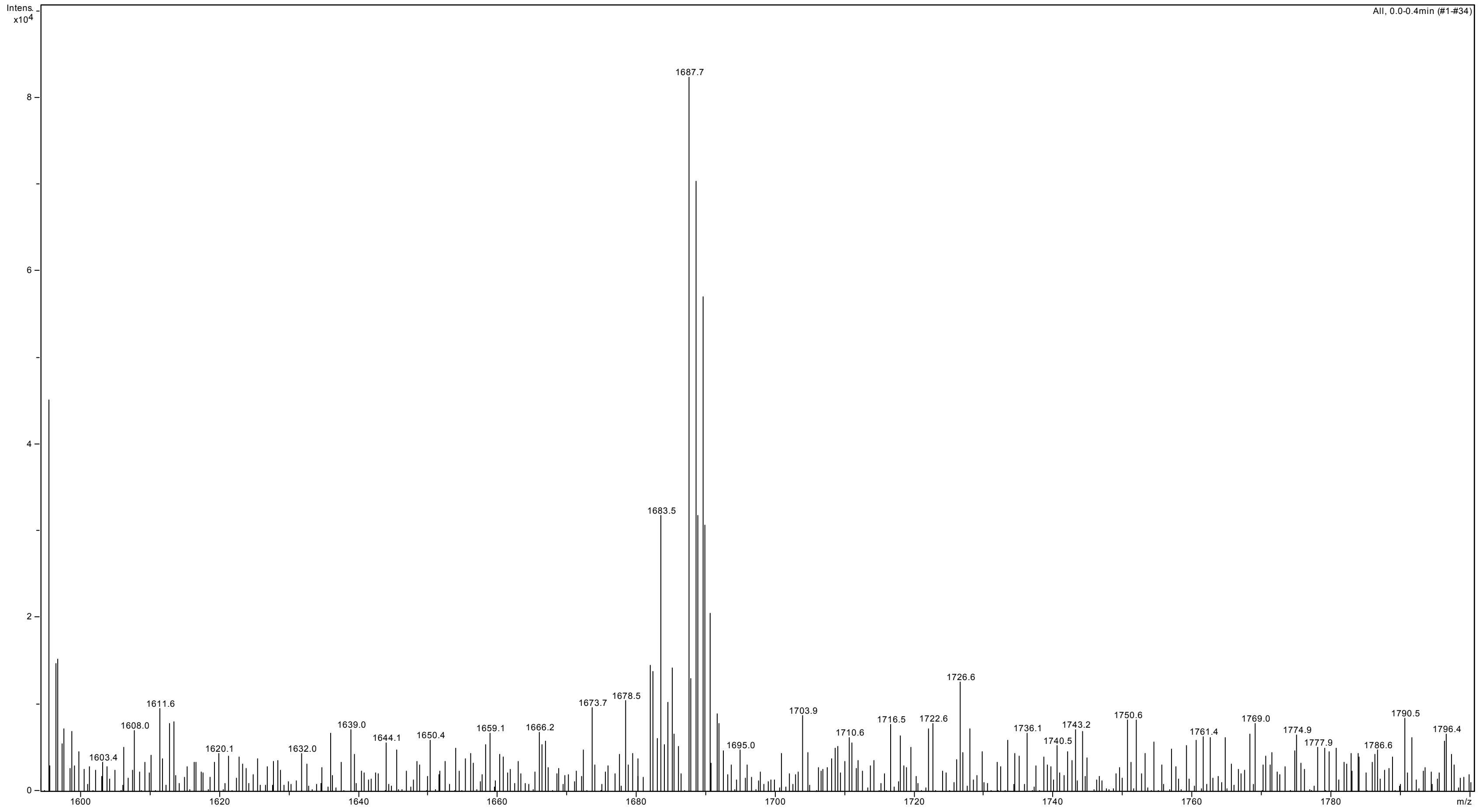

^{1}H NMR of 4-hydroxyphenyl-2,3,4-tri-*O*-benzoyl-6-*O*-*t*-butyldiphenylsilyl-β-D-glucopyranosyl-(1→2)-3,4-di-*O*-benzyl-α-L-rhamnopyranosyl-(1→6)-2,3,4-tri-*O*-benzyl-β-D-glucopyranoside (17a)

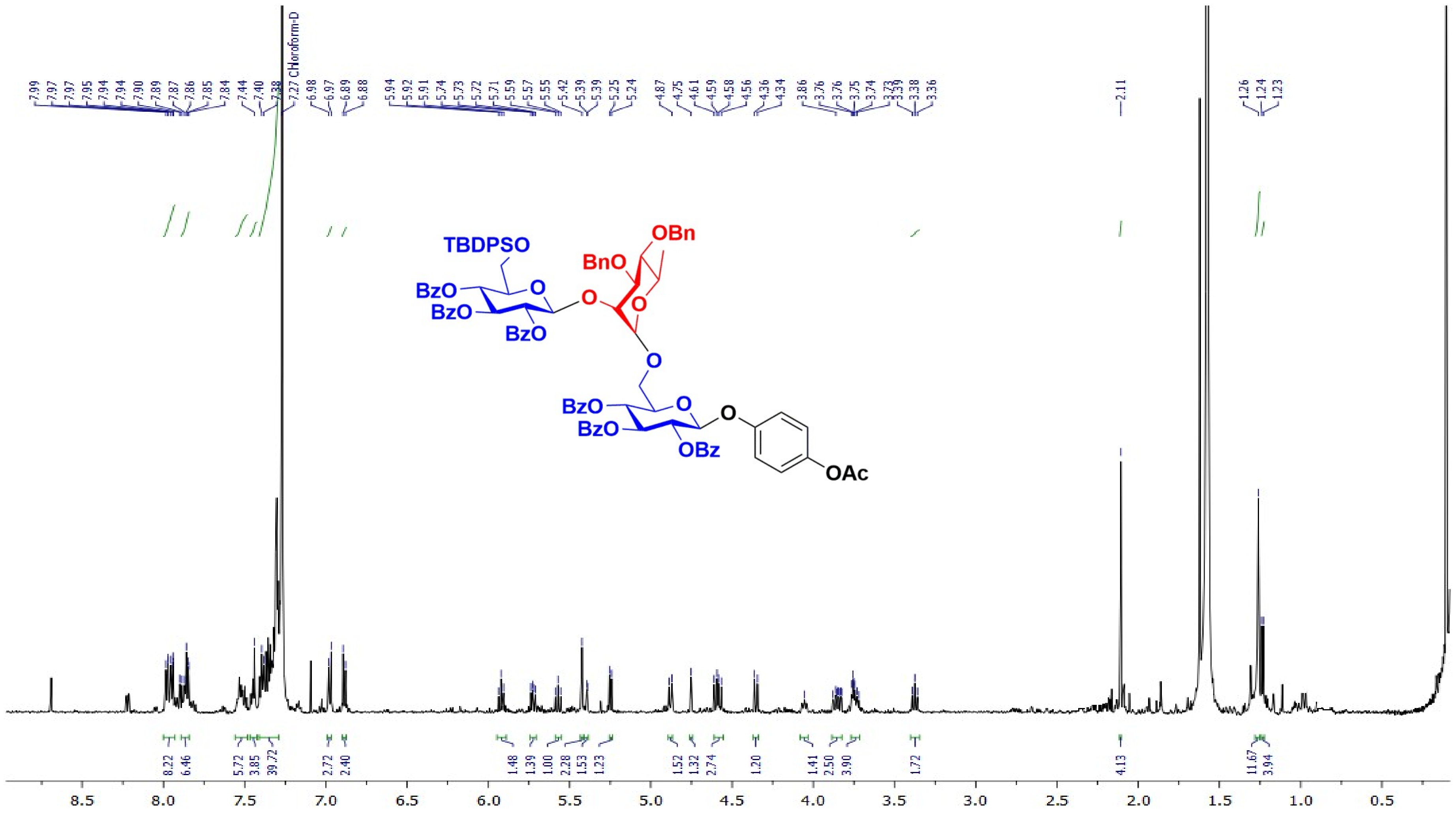

^{1}H NMR of 4-hydroxyphenyl-2,3,4-tri-*O*-benzoyl-6-*O*-*t*-butyldiphenylsilyl-β-D-glucopyranosyl-(1→2)-3,4-di-*O*-benzyl-α-L-rhamnopyranosyl-(1→6)-2,3,4-tri-*O*-benzyl-β-D-glucopyranoside (17a)

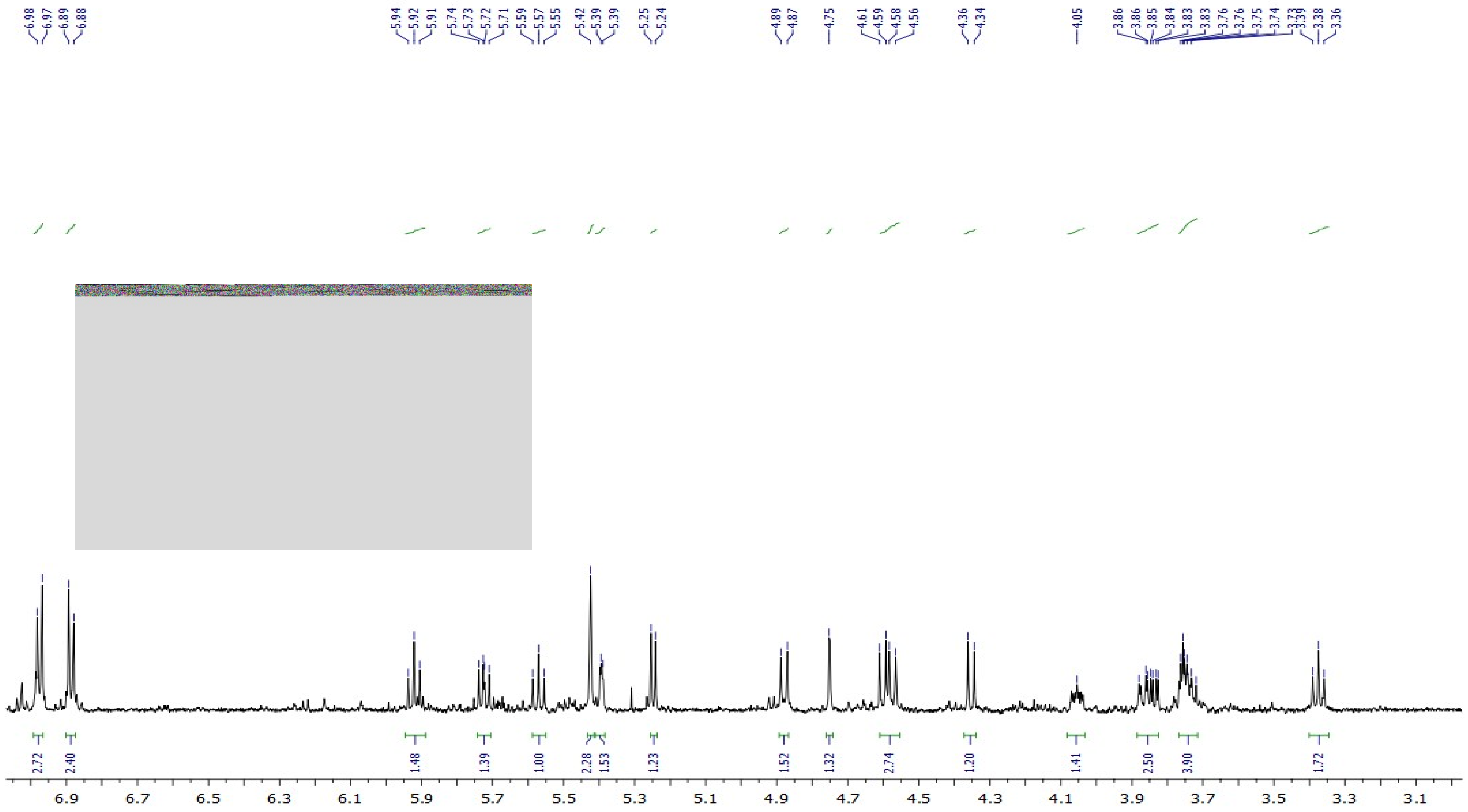

^{13}C NMR of 4-hydroxyphenyl-2,3,4-tri-*O*-benzoyl-6-*O*-*t*-butyldiphenylsilyl-β-D-glucopyranosyl-(1→2)-3,4-di-*O*-benzyl-α-L-rhamnopyranosyl-(1→6)-2,3,4-tri-*O*-benzyl-β-D-glucopyranoside (17a)

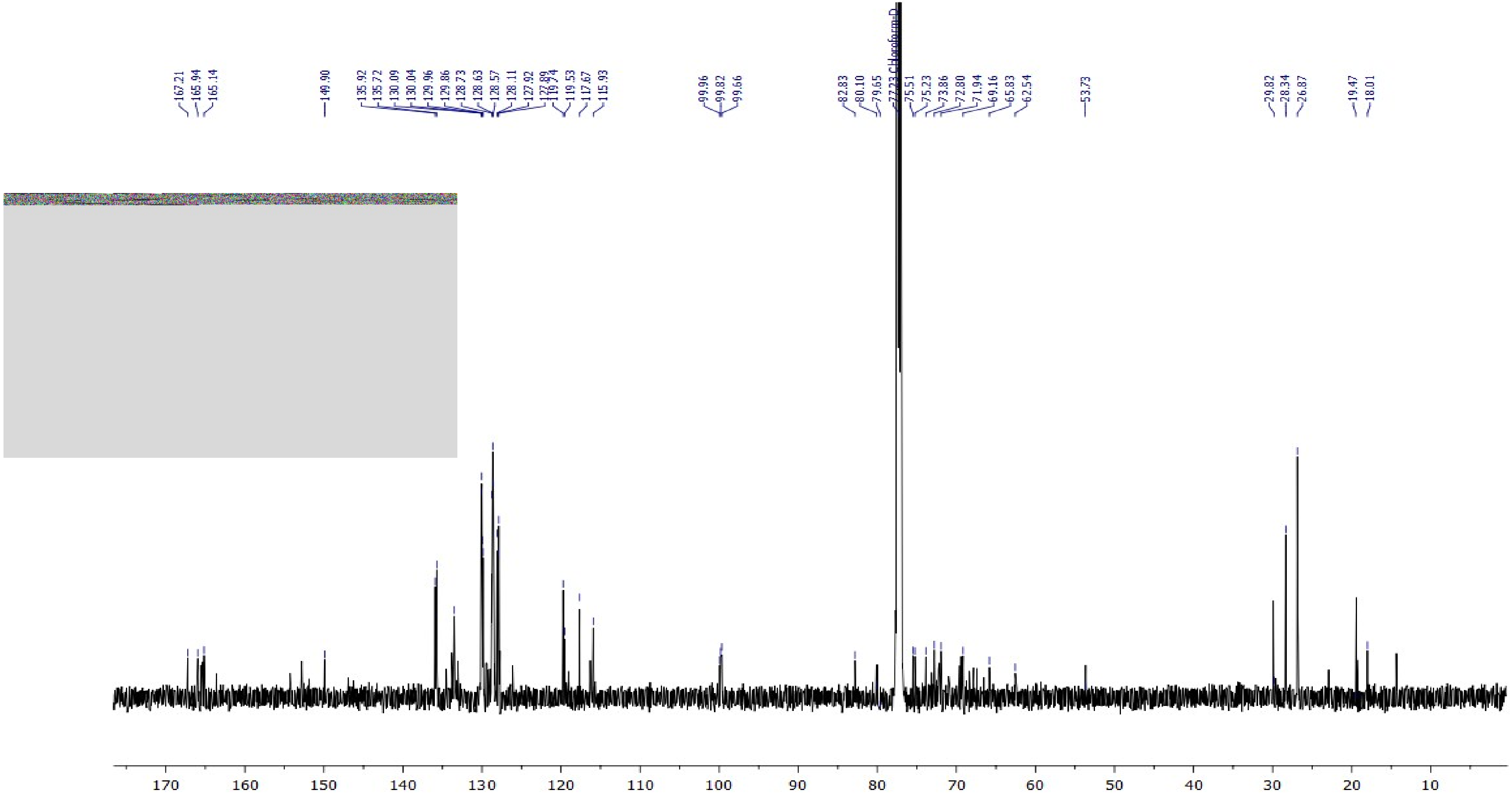

COSY NMR of 4-hydroxyphenyl-2,3,4-tri-*O*-benzoyl-6-*O*-*t*-butyldiphenylsilyl-β-D-glucopyranosyl-(1→2)-3,4-di-*O*-benzyl-α-L-rhamnopyranosyl-(1→6)-2,3,4-tri-*O*-benzyl-β-D-glucopyranoside (17a)

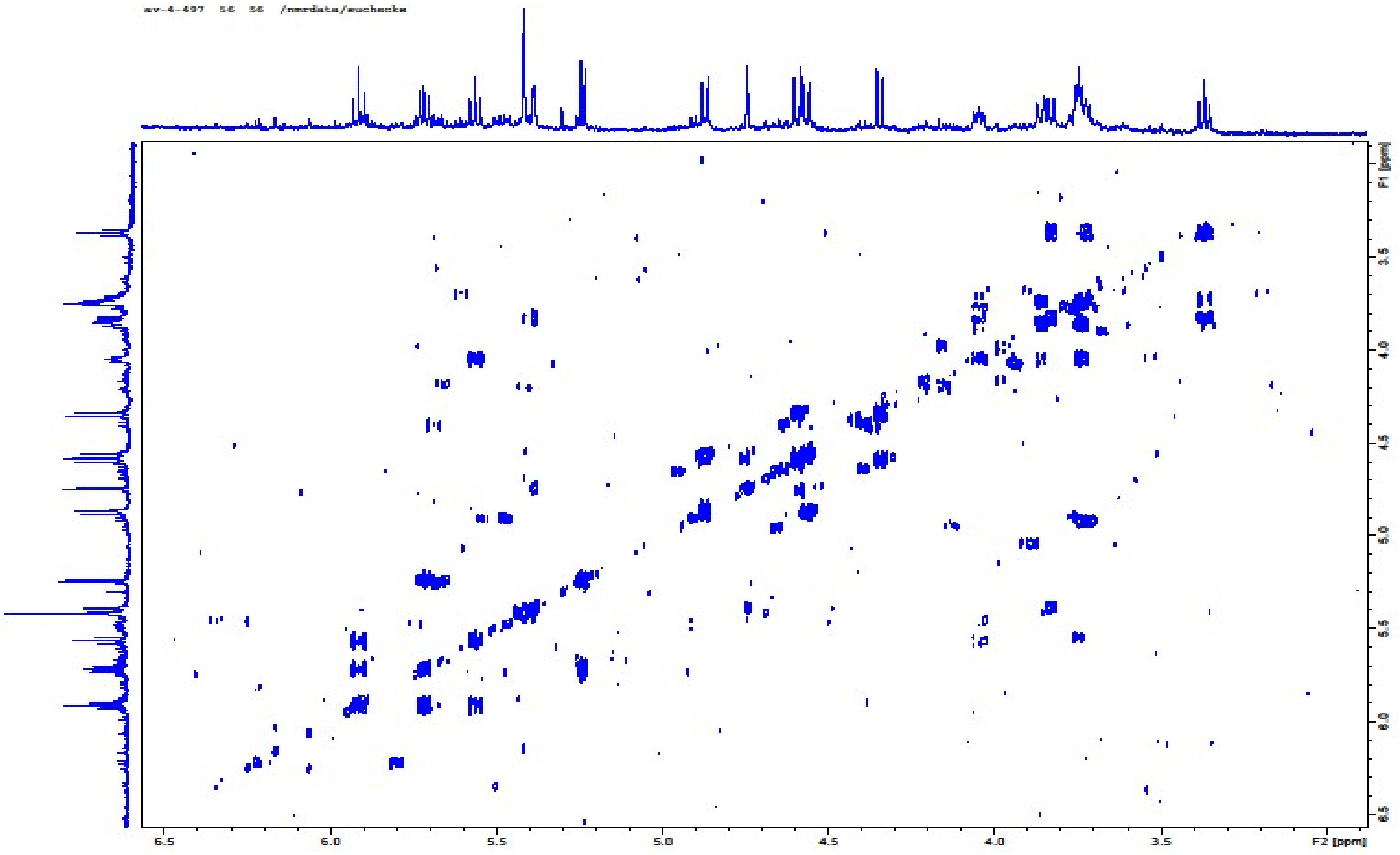

Supplement: RA-008-C8RA08252A-s001 [file RA-008-C8RA08252A-s001.pdf]
